# Safety assessment of ustekinumab in inflammatory bowel disease: a real-world analysis based on the FDA adverse event reporting system (FAERS)

**DOI:** 10.1186/s40001-025-03676-z

**Published:** 2025-12-13

**Authors:** Likang Xu, Zhenkai Huang, Gong Chen, Chao Sun, Yang Yu, Sujun Gao

**Affiliations:** 1 The First School of Clinical Medicine, Faculty of Medicine, Yangzhou University, Yangzhou, Jiangsu China; 2 Department of Internal Medicine, Gaoyou Hospital of Traditional Chinese Medicine, Yangzhou, Jiangsu China; 3 Digestive Department of Northern Jiangsu People’s Hospital, Clinical Medical College of Yangzhou University, Yangzhou, Jiangsu China

**Keywords:** Adverse events, Inflammatory bowel disease, Safety, Ustekinumab, FDA adverse event reporting system (FAERS)

## Abstract

**Background:**

Ustekinumab (UST), a monoclonal antibody that blocks the p40 subunit of both interleukin (IL)-12 and IL-23, is widely used in inflammatory bowel disease (IBD) treatment. As UST use continues to increase in real-world clinical practice, it is becoming increasingly imperative to establish a more comprehensive understanding of its safety profile. The aim was to assess the safety of UST treatment in patients with IBD.

**Methods:**

Data on adverse events experienced by UST-treated patients with IBD were extracted from the U.S. Food and Drug Administration Adverse Event Reporting System (FAERS) for the period of Q4 2009–Q4 2024. Disproportionality was assessed using four statistical measures, including the reporting odds ratio, proportional reporting ratio, multi‑item gamma‑Poisson shrinker method, and Bayesian confidence propagation neural network model. Furthermore, a Weibull distribution analysis was conducted to model the time‑to‑onset of adverse events.

**Results:**

A total of 22687 reports identified UST as the primary suspect drug for IBD and covered 27 system organ classes (SOCs), with injury, poisoning and procedural complications (*n* = 13676), gastrointestinal disorders (*n* = 9563), and infections and infestations (*n* = 8035) being the three with the highest frequency. Positive signals led to the identification of potential adverse events that are not widely documented in the labeling, including urinary tract infections, seizures, hepatic enzyme upregulation, hepatic steatosis, cholelithiasis, cerebrovascular accidents, and transient ischemic attacks. Furthermore, UST‑related adverse events follow an early‑failure model, and their reports gradually decline as treatment duration lengthens.

**Conclusions:**

This study provides comprehensive real-world insights into the safety of UST use in IBD treatment, corroborating known adverse reactions and identifying additional potential risks. Robust pharmacovigilance and long-term monitoring are essential to support personalized UST therapy and facilitate informed risk–benefit assessments.

**Supplementary Information:**

The online version contains supplementary material available at 10.1186/s40001-025-03676-z.

## Introduction

Inflammatory bowel disease (IBD), which includes Crohn’s disease (CD) and ulcerative colitis (UC), is characterized by chronic relapsing inflammation of the intestinal tract, with the typical symptoms being abdominal pain, diarrhea, weight loss, and hematochezia [1, 2]. Over time, IBD can progress, resulting in strictures, fistulas, more frequent infections, or colorectal cancer. Once considered a Western disease, IBD is now being diagnosed with increasing frequency in the Asia–Pacific region and Latin America, thereby shifting the global burden away from Europe and North America [3, 4].

The interleukin (IL)‑12/23-mediated inflammatory cascade is a central driver of IBD pathogenesis [5]. Targeted inhibition of this pathway has become a pivotal therapeutic strategy for IBD. Ustekinumab (UST) is a fully human immunoglobulin G1 kappa (IgG1κ) monoclonal antibody that binds the p40 subunit common to both IL-12 and IL-23, thereby simultaneously blocking IL‑12/23 pathways [6, 7]. Initially approved by the United States (U.S.) Food and Drug Administration (FDA) in 2009 for the treatment of moderate‑to‑severe psoriasis, UST has since been approved for moderate‑to‑severe active CD (in 2016) and UC (in 2019) [8]. The 5‑year IM‑UNITI and 3‑year UNIFI trials demonstrated that UST maintained disease remission with favorable tolerability [9, 10]. Nevertheless, rare or delayed-onset adverse events are sometimes only identified in studies with larger cohorts or after extended follow-up periods, underscoring the need for ongoing real-world surveillance [11].

The U.S. FDA Adverse Event Reporting System (FAERS) is one of the world’s largest post-marketing safety databases. It aggregates spontaneous reports from healthcare professionals, patients, and manufacturers, thereby offering a robust foundation for pharmacovigilance research. Recent studies have identified safety signals for biologics by applying disproportionality methods, such as the reporting odds ratio (ROR), to FAERS data, highlighting the system’s value in the detection of rare or emerging adverse events [12, 13]. To the best of our knowledge, no pharmacovigilance studies have been conducted to evaluate UST safety data in the treatment of IBD.

The aim of this study was to analyze spontaneous reports registered in the FAERS database to detect adverse events associated with UST use in the treatment of patients with IBD. The resulting disproportionality signals offer evidence-based guidance for safer prescribing and long-term monitoring.

## Methods

### Data source and processing

A pharmacovigilance analysis was conducted using data from the FAERS database to evaluate the safety of UST in a post-marketing setting. The FAERS database comprises seven data sets related to demographics (DEMO), drug information (DRUG), adverse events (REAC), outcomes (OUTC), report sources (RPSR), therapy dates (THER), and indications (INDI). UST was approved for CD in 2016, but we started the study period in Q4 2009 to capture possible off-label use in IBD and reports from patients with concomitant psoriasis. All quarterly FAERS files covering the period from Q4 2009 through Q4 2024 were downloaded for analysis.

**Fig. 1:**
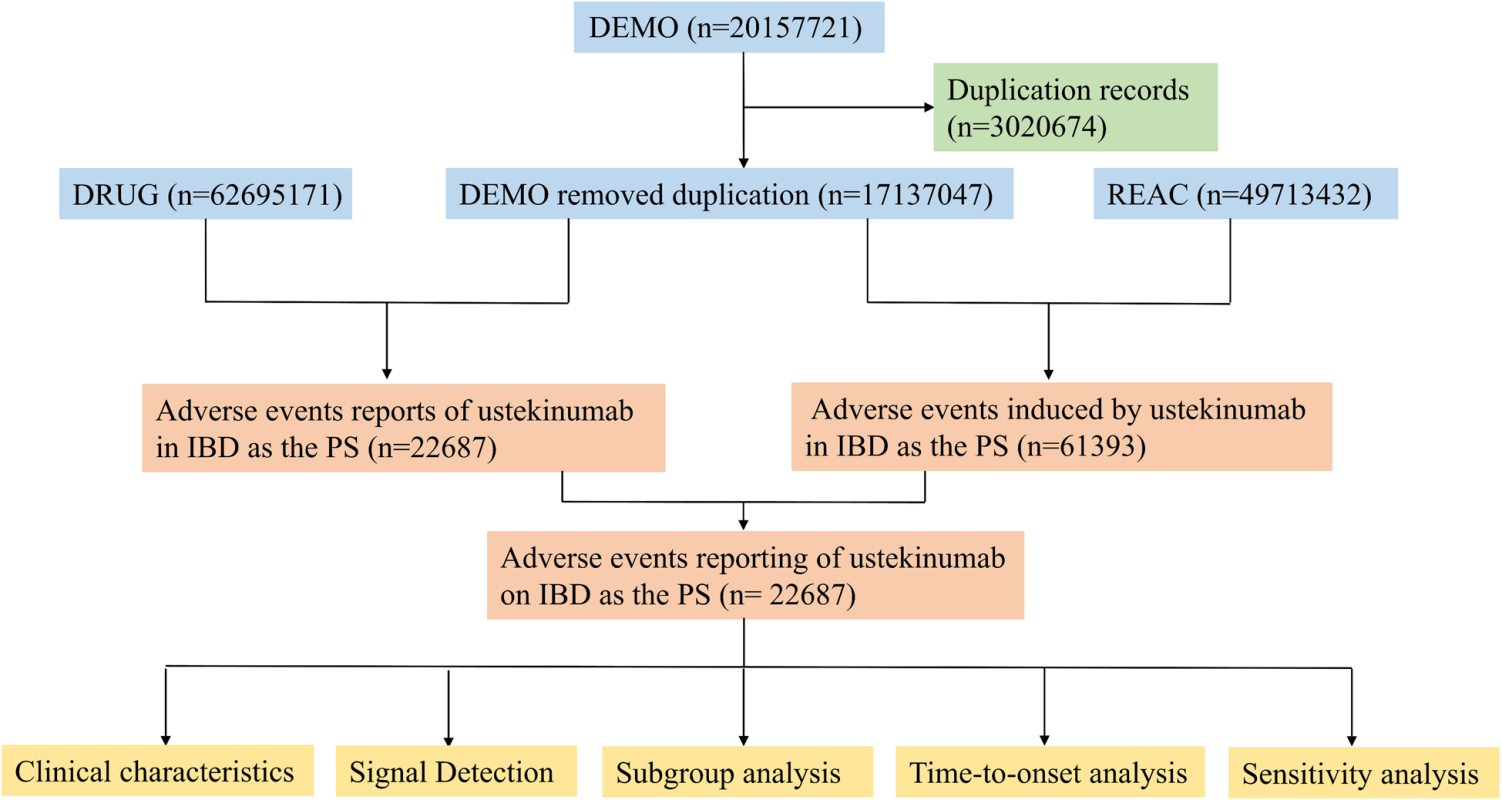
Flowchart for the selection of FAERS reports of ustekinumab-associated adverse events in inflammatory bowel disease. A total of 22687 reports with ustekinumab as the primary suspect drug were included in the analyses. *DEMO* demographics, *DRUG* drug information, *REAC* adverse events, *PS* primary suspect, *IBD* inflammatory bowel disease

The handling of duplicate records was based on the criteria recommended by the U.S. FDA. In situations involving duplicate CASEIDs, the record with the most recent FDA receipt date (FDA_DT) was retained. The data sets were linked via the PRIMARYID field to integrate information across tables. Adverse events were coded in accordance with Medical Dictionary for Regulatory Activities (MedDRA v27.1, RRID:SCR_003751) Preferred Terms (PTs) and aggregated into System Organ Classes (SOCs). Figure 1 shows the flowchart of case selection from the FAERS database. The final cleaned data set contained 17137047 DEMO records, 62695171 DRUG records, and 49713432 REAC records. Based on the ROLE_COD field in the DRUG table, we included only adverse event reports in which UST was coded as the primary suspect (PS) and excluded those in which it appeared only as a secondary suspect or concomitant drug. In total, 22687 reports met these criteria.

All deduplicated reports were retained for analysis even if some demographic or clinical fields were missing. Missing values were not imputed. For analyses that required specific variables, such as age, sex, pregnancy status and outcome seriousness, only reports with non-missing information for the relevant field were included.

### Statistical analysis

Four widely used disproportionality methods were employed to detect UST-associated signals, including the ROR, proportional reporting ratio (PRR), Bayesian confidence propagation neural network (BCPNN) model, and multi‑item gamma–Poisson shrinker (MGPS) method [14]. These four algorithms capture complementary statistical properties. ROR and PRR are rapid and transparent frequentist metrics, whereas BCPNN and MGPS use Bayesian shrinkage to stabilize risk estimates in sparse data. Applying them in parallel improves both the sensitivity and the specificity of signal detection in FAERS [15]. Recent comparative pharmacovigilance studies, therefore, recommend using a multi-algorithm approach rather than relying on any single metric [16]. The 2 × 2 contingency tables underlying each calculation are provided in Supplementary Table 1, and the formulae with pre-specified signaling thresholds are provided in Supplementary Table 2. An event was classified as having a positive signal only if it exceeded the threshold of at least one algorithm.

Subgroup analyses were conducted for four dimensions including age, sex, pregnancy status, and event seriousness to explore the distribution of adverse events across different patient groups. Age was grouped as younger than 18 years, 18–65 years, and older than 65 years. Serious outcomes were defined as events that resulted in death, were life-threatening, required hospitalization, led to disability, or were classified as other medically important serious conditions. Time‑to‑onset (TTO) was defined as the interval from the administration of the first dose of UST to the date of the reported adverse event. The median and interquartile range (IQR) were used to describe the TTO distribution of UST-associated adverse events. A Weibull distribution was applied, with scale (α) and shape (β) parameters, to model changes in the risk of adverse events over time. To evaluate the robustness of the findings, sensitivity analyses were conducted in which medications commonly co-prescribed with UST were excluded. All statistical analyses were performed using R software (version 4.4.1, RRID:SCR_001905).

## Results

### Descriptive characteristics

Table 1 summarizes the characteristics of the adverse‑event reports in which UST was designated as the PS drug for IBD. In total, 22687 adverse‑event reports were collected. Among the 22687 reports, 215 were submitted between 2009 and 2015, and 22472 were submitted from 2016 onwards. With respect to sex, 12849 (56.6%) involved female patients, 8138 (35.9%) involved male patients, and 1700 (7.5%) did not specify the sex of the patient. Nearly half (49.0%) of the reports involved adults aged 18–65 years; this represented the largest proportion among the age categories. Healthcare professionals submitted 57.7 % of the reports. FAERS data indicate that most reports of UST-associated adverse events in IBD involve adult patients treated in North America and Europe. Most of the reports originated in the U.S. (*n* = 12913), followed by Canada (*n* = 4659) and the United Kingdom (*n* = 1666). The number of reports peaked in 2020 at 5607 (representing 24.7% of the data set).

**Table 1:** Clinical characteristics of Ustekinumab adverse event reports from the FAERS database (Q4 2009–Q4 2024)

Characteristics	Case numbers	Case proportion (%)
Number of events	22687	100
Gender		
Female	12849	56.6
Male	8138	35.9
Miss	1700	7.5
Age		
<18	649	2.9
18–65	11117	49.0
>65	2015	8.9
Miss	8906	39.3
Top 3 reported countries		
United States	12913	56.9
Canada	4659	20.5
United Kingdom	1666	7.3
Reporter		
Healthcare professional	13093	57.7
Non-healthcare professional	9278	40.9
Miss	316	1.4
Reporting year		
2010	1	0.0
2011	1	0.0
2012	16	0.1
2013	23	0.1
2014	36	0.2
2015	138	0.6
2016	201	0.9
2017	985	4.3
2018	2199	9.7
2019	2648	11.
2020	5607	24.7
2021	2770	12.2
2022	3059	13.5
2023	3052	13.5
2024	1951	8.6

### Signal detection

**Fig. 2:**
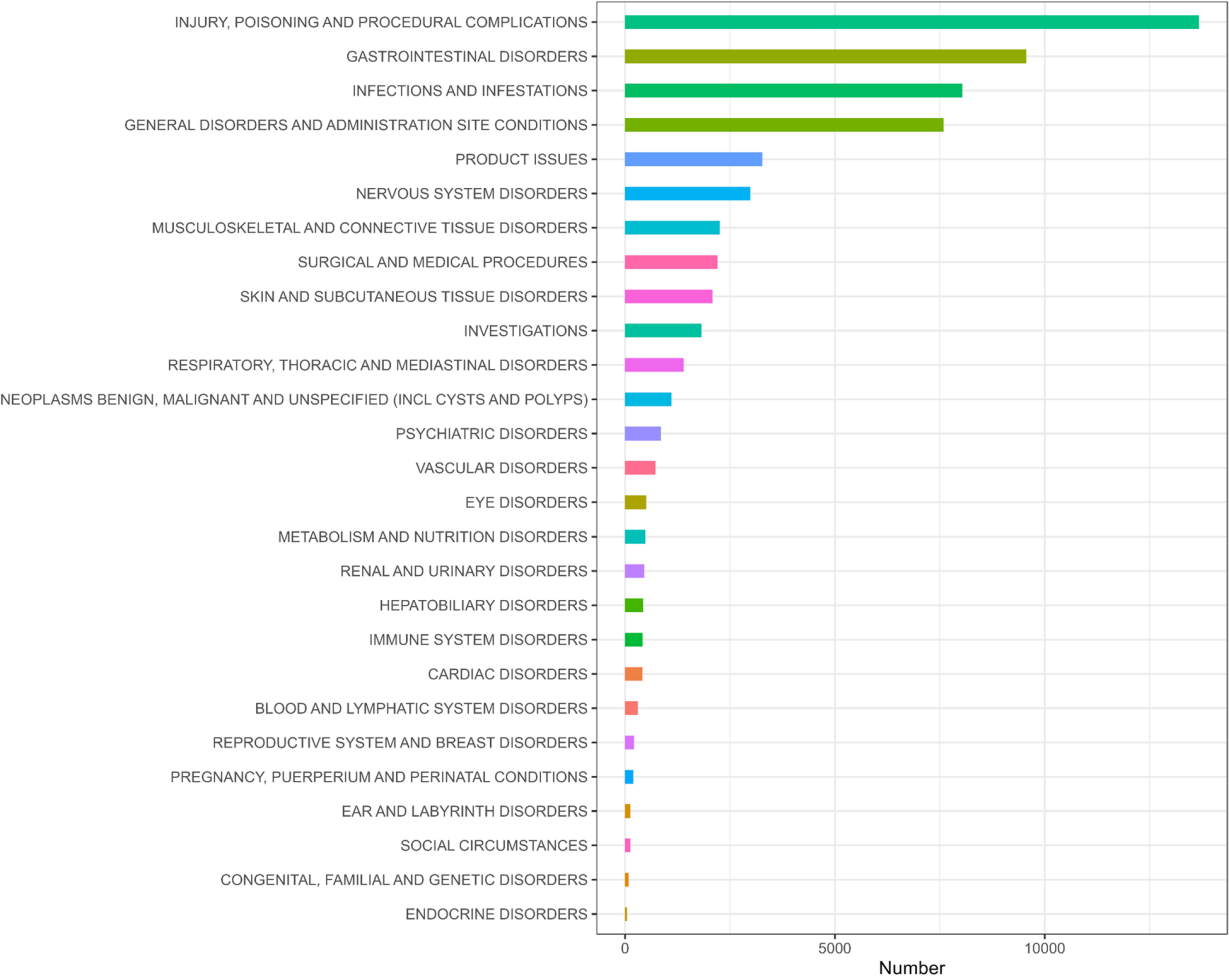
Distribution of ustekinumab-associated adverse events in inflammatory bowel disease by MedDRA SOC. Bars show the number of reports in each SOC

At the SOC level, adverse events were distributed across a wide range of organ systems, and Fig. 2 summarizes the reporting frequency for each SOC. Supplementary Table 3 summarizes the signal strength for each SOC. The three most frequent SOCs were injury, poisoning and procedural complications (*n* = 13676), gastrointestinal disorders (*n* = 9563), and infections and infestations (n = 8035). The following eight SOCs generated positive signals: (1) injury, poisoning and procedural complications; (2) infections and infestations; (3) product issues; (4) nervous system disorders; (5) surgical and medical procedures; (6) neoplasms—benign, malignant and unspecified (including cysts and polyps); (7) hepatobiliary disorders; and (8) congenital, familial, and genetic disorders.

The 100 most frequently reported PTs and the 100 PTs with positive signals are summarized in Supplementary Tables 4 and 5. The analysis also identified infrequently reported or label-unlisted adverse events, including migraines, urinary tract infections, cerebrovascular accidents (CVAs), transient ischemic attacks (TIAs), hepatic injury, cholelithiasis, Clostridium difficile (C. difficile) infection, seizures and visual impairment. Overall, infections account for most PT-level signals. Malignant neoplasm terms form a smaller group of positive signals. Less frequent hepatic, biliary, neurological and thromboembolic events indicate additional, less common safety concerns associated with UST.

### Subgroup analyses

The sex subgroup analysis showed that in male patients, signals mainly involved urticaria, cerebrovascular events, abdominal abscesses, seizures, gastrointestinal infections and visual impairment, whereas in female patients they clustered around respiratory infections, urinary tract infections, sepsis and hypersensitivity reactions. Detailed data are provided for the two sexes in Supplementary Tables 6 and 7.

The subgroup analysis based on age revealed that in pediatric patients, vigilance should be exercised to detect the occurrence of anaphylactic reactions, nephrolithiasis, intestinal abscesses, appendicitis, dysphagia, flushing, and disseminated tuberculosis. Adult patients should be monitored for the occurrence of pneumonia, lower respiratory tract infections, sinusitis, influenza, C. difficile infection, migraines, cellulitis, loss of consciousness, and elevated hepatic enzyme levels. Finally, in elderly patients, the results demonstrated that surveillance is warranted for the detection of pneumonia, lower respiratory tract infections, rashes, C. difficile infection, cellulitis, urinary tract infections, diverticulitis, confusion, malignant neoplasms, visual impairment, urticaria, and cholelithiasis. The signal intensities for these age-related adverse events are provided in Supplementary Tables S8–10.

The subgroup analysis according to outcome severity showed distinct profiles of clinically meaningful adverse events. In the non-serious group, cutaneous and hypersensitivity reactions were most frequent, including rash, urticaria, pruritus, flushing, and alopecia. Migraine, chest discomfort, visual impairment, and increases in hepatic enzyme levels were also reported among non-serious outcomes. It is noteworthy that signals for hepatic enzyme elevation were confined to non-serious reports, suggesting that most hepatic events were mild. In contrast, serious outcomes were mainly characterized by infections involving the respiratory, gastrointestinal, urinary, and soft tissue systems. Intestinal and perianal complications and malignant neoplasms were also observed among serious outcomes. These patterns are detailed in Supplementary Tables 11–12.

In the pregnancy subgroup, the predominant signals involved infectious complications and pregnancy-related adverse events. Statistically significant disproportionality signals were observed for pregnancy-related events, such as abortion spontaneous, premature baby, caesarean section, premature labor, and congenital anomaly. Disproportionality signals were also detected for infectious events, including urinary tract infection, *C. difficile* infection, abscess, gastroenteritis, pyelonephritis, *Escherichia* infection, and upper respiratory tract infection, as detailed in Supplementary Table 13.

### TTO and Weibull distribution analysis of adverse events

**Fig. 3:**
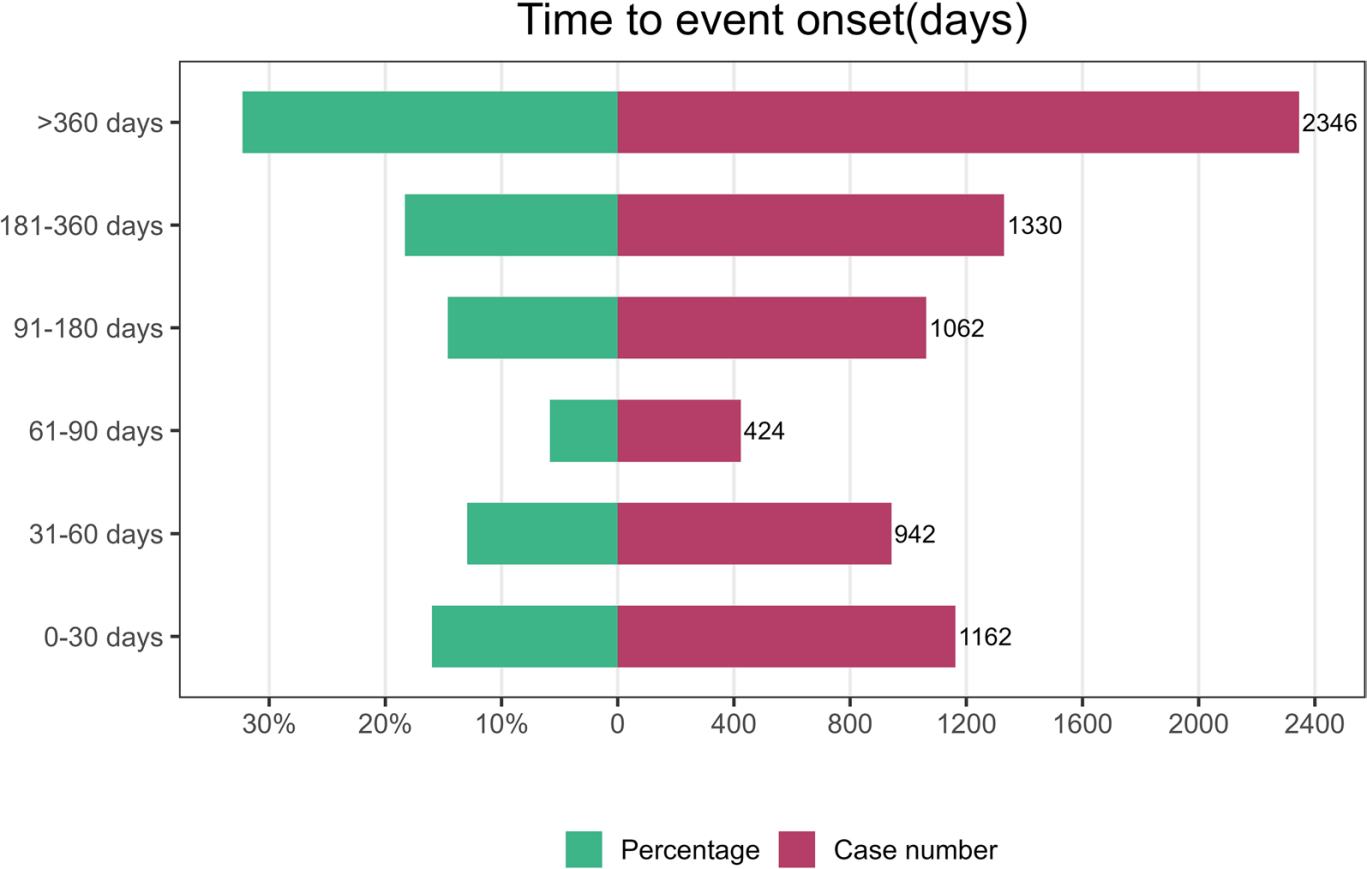
Time to onset of adverse events induced by ustekinumab in patients with inflammatory bowel disease. Bars show the number and percentage of reports in each predefined time interval after treatment initiation

**Fig. 4:**
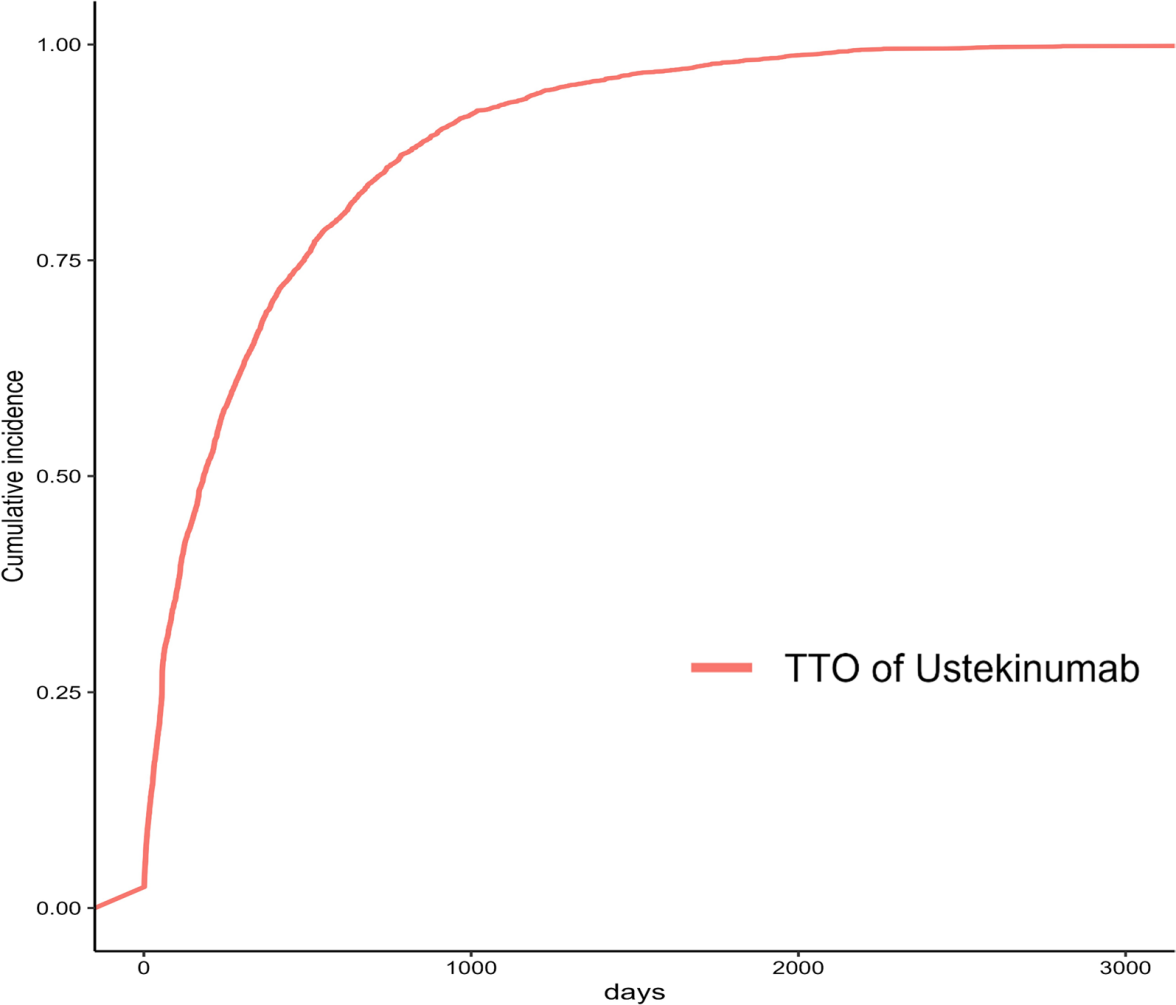
Cumulative incidence of adverse events related to ustekinumab in patients with inflammatory bowel disease over time. Adverse events occurred predominantly in the early phase of ustekinumab therapy, with only a modest increase at later timepoints

A total of 7266 reports documented the TTO of adverse events, the temporal distribution of which is illustrated in Fig. 3. Approximately 16% of the events occurred within the first month after treatment initiation, whereas more than 30% occurred after the first 360 days. Figure 4 depicts the temporal trend in the incidence of adverse events. The curve rises steeply during the early treatment period and then gradually approaches a plateau, suggesting that adverse events accumulated mainly during the early phase of UST therapy. The TTO was modelled using a Weibull distribution, and the resulting shape parameter was indicative of an early failure model, with the hazard of adverse events decreasing over time. More specific parameters are detailed in Table 2.

**Table 2:** Time to onset of Ustekinumab-associated adverse events and Weibull distribution analysis

Drug	TTO (days)		Weibull distribution		
	Case reports	Median (d) (IQR)	Scale parameter: *α* (95% CI)	Shape parameter: *β* (95% CI)	Type
Ustekinumab	7266	183 (56, 490)	298.72 (289.02, 308.41)	0.75 (0.73, 0.76)	Early failure

*TTO* time to onset, *CI* confidence interval, *IQR* interquartile range

### Sensitivity analysis

In IBD therapy, UST is often prescribed in combination with immunomodulators, such as thiopurines or methotrexate, corticosteroids, and anti-inflammatory agents like 5-aminosalicylic acid. After excluding co-medications, the analyses were repeated to identify the 100 most frequent adverse event PTs. The resulting profile was consistent with the findings of the earlier analyses and comprised headaches, pneumonia, infusion‑related reactions, lower respiratory tract infections, urinary tract infections, *C.* *difficile* infection, abscesses, influenza, sinusitis, migraines, cellulitis, sepsis, CVAs, bronchitis, and seizures. The signal strengths from the sensitivity analysis are reported in Supplementary Table 14.

## Discussion

This study analyzed FAERS records from Q4 2009 through Q4 2024 and identified 22,687 adverse event reports involving UST use in patients with IBD.

Infections were among the most common adverse events reported, which is consistent with the immunosuppressive effects of UST. In a long-term follow-up study involving patients with UC, nasopharyngitis, pneumonia, and upper respiratory tract infections were the most frequently reported adverse events [17]. In a separate randomized, double-blind, placebo-controlled maintenance trial involving patients with UC, the incidence of influenza was higher in the UST-treated group than in the placebo group, but the difference did not reach statistical significance [18]. Seasonal influenza vaccination may be a safe and potentially beneficial strategy for this population [19]. UST can also trigger rare non-infectious pneumonitis, such as interstitial, eosinophilic and cryptogenic organizing pneumonia [20, 21]. Respiratory symptoms should, therefore, be monitored carefully. The frequency of gastrointestinal infections in these patients is increased, including diverticulitis, gastroenteritis, viral gastroenteritis and C. difficile infection. C. difficile infection is one of the most frequently reported adverse events in clinical trials [22]. Urinary tract infections are also common during UST treatment for psoriasis but are less frequently reported during IBD treatment. In our FAERS analysis, urinary tract infections, particularly kidney infections and cystitis, were reported more often than expected, and these patterns warrant further clinical validation. Overall, the risk of infection during UST treatment for IBD remains low, and few serious events have been reported [23, 24]. However, we detected a positive signal for sepsis in female patients, which suggests a potentially higher risk of life-threatening systemic infections in women. In pediatric patients, a positive signal was detected for disseminated tuberculosis, which is consistent with the critical role of IL-12 in anti-tuberculosis immunity. There is currently insufficient evidence to confirm that UST meaningfully increases the risk of tuberculosis [25]. Furthermore, the relatively higher number of appendicitis reports among pediatric patients with IBD undergoing treatment with UST warrants further investigation. Consistent with these observations, the severity subgroup analysis showed that serious outcomes were dominated by infections of the respiratory, gastrointestinal, urinary and soft tissue systems. Intestinal and perianal complications and malignant neoplasms were likewise mainly reported in serious cases, although the numbers were relatively small. The data collectively suggest that clinicians should remain highly vigilant for signs of infection during UST treatment for IBD, particularly in patients with a history of recurrent infection or other risk factors. Pretreatment risk assessments for infection and regular monitoring during therapy are recommended. Immediate discontinuation of UST and the initiation of appropriate therapeutic interventions should be implemented in cases in which severe infections are detected.

In the pregnancy subgroup, we observed disproportionality signals for pregnancy-related adverse events, mainly spontaneous abortion, preterm delivery, caesarean section, congenital anomalies and infections such as urinary tract infection, C. difficile infection, gastroenteritis and pyelonephritis. These patterns indicate that use of UST during pregnancy should be accompanied by structured risk assessment and close safety monitoring. For women who plan pregnancy or are already pregnant, clinicians need to balance the benefit of maintaining disease control with UST against the possibility of these pregnancy-related adverse events. Preconception and early pregnancy assessment should review previous pregnancy history, concomitant immunosuppressive therapy, infection history and comorbidities, and clear counselling based on this information can support shared decisions on whether to continue UST, adjust the regimen or switch to an alternative agent. In patients whose remission is strongly dependent on UST, continuation of therapy may be acceptable after an individualized risk–benefit evaluation. In such cases, specialist follow-up and intensified clinical and laboratory monitoring should be maintained throughout pregnancy. When early signs of urinary, gastrointestinal or systemic infection are detected, clinicians should make a prompt diagnosis, start timely anti-infective treatment and consider temporary interruption or modification of UST. For high-risk women, early collaboration between gastroenterologists and obstetric teams, together with planned postnatal follow-up of exposed infants, may help to reduce pregnancy-related adverse events and improve long-term outcomes.

A higher frequency of skin-cancer reports was observed in male patients, possibly owing to the impaired immunosurveillance induced by immunosuppression. Notably, many patients with IBD are concurrently administered thiopurines, which have been shown to increase the risk of non-melanoma skin cancer (NMSC) as well as hematologic malignancies, urinary tract tumors, and cervical cancer, which could have confounded the interpretation of the skin cancer signals observed in this study [26]. Skin and lung malignancies were reported more frequently in older patients, likely owing to age-related immunosenescence and accumulated environmental exposures. Although breast cancer is the most common malignancy among patients with IBD, there is currently no conclusive evidence linking its increased incidence to UST treatment [27]. A small number of case reports have documented melanoma in patients receiving UST. One case report detailed a 42-year-old individual with CD who developed amelanotic malignant melanoma after prolonged treatment [28]. UST therapy may impair immune-mediated tumor surveillance and could promote tumor growth [29]. However, to date, large randomized trials and meta-analyses have not found a significant increase in the risk of malignancies or NMSC in IBD patients treated with UST [30]. Given these uncertainties, regular age and risk appropriate cancer screening is recommended for high-risk patients, with particular attention to skin and lung malignancies. Long term observational studies are still needed to clarify these associations and to refine individualized risk stratification and treatment decisions.

Our analysis identified headaches, migraines, seizures, and vertigo as the main UST-related neurological adverse events. Approximately 50% of patients with IBD experience dizziness or vertigo irrespective of treatment, and a clear causal relationship with UST has not been established [31]. Most reported seizures occurred in male patients. Seizures may be linked to posterior reversible encephalopathy syndrome (PRES). PRES is a rare but potentially severe neurological complication whose typical features include headache, seizures, altered consciousness, visual disturbances, and posterior white matter edema on neuroimaging [32]. To date, three cases of PRES related to UST therapy in patients with IBD have been reported. Mishra and colleagues reported two PRES cases during UST induction for CD, and Jordan described a further case during maintenance therapy, all of which improved after UST withdrawal and appropriate treatment [33, 34]. Clinicians should ask routinely about new neurological symptoms during follow-up. Prompt neurologic assessment and imaging are warranted when events, such as seizures, visual disturbance or severe headache occur.

CVAs and TIAs were the main positive adverse event signals in the cerebrovascular system. Ischemic stroke usually follows thrombotic or embolic occlusion, underscoring thrombogenesis as a plausible mechanism. Additional factors that heighten thromboembolic risk in IBD—corticosteroid therapy, oral contraceptives, acute flares and recent surgery—must be taken into account when interpreting these signals [22]. A recent meta-analysis linked anti-IL-12/23 therapy to a higher incidence of major adverse cardiovascular events, suggesting that IL-12/23 blockade might destabilize atherosclerotic plaques and precipitate stroke [35, 36]. Given these signals, cardiovascular risk factors should be assessed and optimized before UST initiation. Patients with established cardiovascular or cerebrovascular disease should be monitored more closely during treatment. Large prospective studies are still needed to quantify the magnitude of this risk and to clarify the underlying mechanisms.

The signal detection methods also revealed adverse events involving the hepatobiliary system, including liver enzyme upregulation, hepatic steatosis, and cholelithiasis. The elevated liver enzyme levels observed in some patients indicate that UST therapy may disturb hepatic function, although the reported abnormalities have generally been mild (grade 1) and reversible. In a retrospective study involving 44 patients being treated with UST for psoriasis, six experienced grade 1 transaminase elevation [37]. In our severity subgroup analysis, elevations in hepatic enzyme levels occurred almost exclusively in non-serious outcomes. This finding supports the notion that most hepatic events under UST treatment are mild and reversible in clinical practice. Even so, UST should be administered with caution, and liver tests should be closely monitored in individuals with hepatic impairment, cirrhosis, or hepatitis. A positive cholelithiasis signal was also noted in older adults. The mechanism is unclear and requires confirmation in prospective cohorts. Before initiating UST therapy for IBD, clinicians should evaluate baseline hepatic function and gallbladder status. During therapy, liver enzyme levels should be quantified regularly, and gallbladder ultrasonography can be considered when right-upper-quadrant symptoms emerge or in patients with known gallstone risk factors. If an upregulation of transaminase levels is detected, treatment may be adjusted or temporarily discontinued, and further hepatic evaluation or intervention should be considered. If cholelithiasis develops, gallbladder ultrasonography should be used to monitor gallstone formation and guide further management.

Allergic reactions constitute a common class of adverse events, with male and older patients more likely to report urticaria, whereas female patients predominantly report pruritus and flushing. One case series in pediatric and young adult patients with IBD reported a wide spectrum of UST-related hypersensitivity reactions affecting the respiratory, cardiovascular, cutaneous and gastrointestinal systems [38]. To mitigate such risks, clinicians should review the allergy history of each patient before initiating treatment. In patients with a history of severe drug hypersensitivity, a graded-dose challenge may be considered, although routine skin testing for UST is not standard practice, and emergency equipment and medications should be readily available to treat severe reactions. Alternative therapies should be considered if the patient is known to be allergic to UST.

Weibull modeling shows that UST-associated adverse events follow an early failure pattern, with the risk peaking soon after treatment starts and then declining over time in a long-tail distribution. This temporal profile points to a biphasic risk trajectory. Early events are most likely attributable to immediate pharmacologic or immunologic reactions that occur after the first exposure, whereas later sporadic events may reflect cumulative dose, concomitant therapies, or progression of the underlying disease. These observations justify a tiered surveillance strategy. Clinicians should intensify monitoring during the first 1–3 months, with special attention to infections, hypersensitivity reactions, and neurological manifestations. During the maintenance phase, less frequent but still routine assessments remain appropriate. In patients with higher baseline risk—such as older adults or those with cardiovascular comorbidities or a history of infection—regular evaluation should continue throughout therapy. Recognizing this time dependent hazard pattern helps clinicians concentrate intensive monitoring in the early high-risk phase and deescalate surveillance thereafter.

These findings refine how UST can be used within precision medicine and public health surveillance. At the individual level, baseline factors such as age, comorbidities, history of serious infection or malignancy and pregnancy status can be combined with UST specific patterns of adverse events to stratify patients by risk before starting or continuing therapy. On this basis, clinicians can decide whether to initiate UST, whether to continue it and how to choose between UST and alternative treatments. Risk stratification also informs the planned frequency of monitoring and the thresholds for investigation, intervention or dose adjustment during UST therapy. At the population level, spontaneous reports of UST-related adverse events in FAERS can be linked with disease registries, administrative claims and electronic health records. Such integrated data sets can improve early detection of UST safety signals in specific subgroups or regions, support targeted risk minimization programs and provide a stronger evidence base for updating UST prescribing information and IBD treatment guidelines.

Although the findings of this study are important, some limitations must be acknowledged. First, the FAERS database cannot be used to calculate population-level incidence rates of adverse events; this shortcoming impedes attempts to accurately estimate the true event frequency, thereby limiting the generalizability of the results. Second, because most FAERS reports originate from North America and Europe, Asian populations remain under-represented. This regional skew in reporting may limit the extrapolation of our findings to regions with different prescribing patterns and pharmacovigilance practices. Third, as a spontaneous reporting system, the FAERS is susceptible to under-reporting, particularly of mild or well-recognized events, and to missing or duplicate reports, introducing the possibility of bias, affecting the accuracy of the data, and compromising the reliability and completeness of the analyses. In addition, publicity about specific drugs or events and regulatory actions may lead to stimulated reporting, which can distort the apparent strength of some signals. Fourth, because information on dose and dosing interval was severely incomplete and recorded in a very disorganized way, we did not further analyze potential differences between dosing regimens. Finally, although disproportionality analyses are an effective means of signal detection, they cannot establish a causal relationship between UST use and the identified events. Prospective studies are essential to confirm these findings and to deepen the medical community’s understanding of the safety profile of UST.

## Conclusion

This study systematically analyzed FAERS reports on adverse events related to the use of UST in the treatment of IBD from Q4 2009 to Q4 2024. The findings not only corroborated several established safety signals, but also revealed new potential risks associated with real-world UST use in this patient population. Infections were the most frequently reported adverse events, underscoring the need for both vigilant clinical monitoring and further studies to identify the underlying mechanisms and improve prevention strategies. Several uncommon adverse events were also detected that were not explicitly listed in the drug’s labeling, including urinary tract infections, such as kidney infections, seizures, elevated liver enzyme levels, hepatic steatosis, cholelithiasis, CVAs, and TIAs. The TTO analysis revealed an early failure hazard pattern with a persistent long tail, underscoring that UST-related risks change dynamically over the treatment course. These preliminary insights highlight the necessity of persistent pharmacovigilance to monitor and prevent adverse events related to the use of UST for IBD. UST is nevertheless generally regarded as one of the relatively safer biologic and small molecule options for IBD. Our findings refine this overall safety profile by delineating specific patterns of time to onset and highlighting adverse events that may warrant targeted clinical monitoring.

## Supplementary Information

Supplementary material 1

## Data Availability

This study used publicly available data from the FDA Adverse Event Reporting System (FAERS).

## Abbreviations

BCPNN: Bayesian Confidence Propagation Neural Network
CD: Crohn’s disease
CVAs: Cerebrovascular accidents
DEMO: Demographics (FAERS category)
DRUG: Drug information (FAERS category)
FAERS: Food and Drug Administration Adverse Event Reporting System
FDA: Food and Drug Administration
FDA_DT: Food and Drug Administration receipt date
IBD: Inflammatory bowel disease
IgG1κ: Immunoglobulin G1 kappa
IQR: Interquartile range
IL: Interleukin
INDI: Indications (FAERS category)
MedDRA: Medical Dictionary for Regulatory Activities
MGPS: Multi‑item Gamma–Poisson Shrinker
NMSC: non-melanoma skin cancer
OUTC: Outcomes (FAERS category)
PRR: Proportional reporting ratio
PS: primary suspect
PTs: Preferred terms
REAC: Adverse events (FAERS category)
ROR: Reporting odds ratio
RPSR: Report sources (FAERS category)
SOC: System organ class
Th1: T helper 1
Th17: T helper 17
THER: Therapy dates
TIAs: Transient ischemic attacks
TTO: Time-to-onset
UC: Ulcerative colitis
UST: Ustekinumab
