# Supplementary material for: Safety assessment of ustekinumab in inflammatory bowel disease: a real-world analysis based on the FDA adverse event reporting system (FAERS)
Source: Eur J Med Res. 2025 Dec 13;31:93. doi: 10.1186/s40001-025-03676-z (PMC12817861; doi:10.1186/s40001-025-03676-z)
Supplement: Supplementary file 1 — Supplementary material 1 [file 40001_2025_3676_MOESM1_ESM.docx]

Supplementary Material

Supplementary Tables

Supplementary Table 1:

Two-by-two contingency table for disproportionality analyses.

|  | Target AEs | Other AEs | Total |
| --- | --- | --- | --- |
| Ustekinumab | a | b | a+b |
| Other drugs | c | d | c+d |
| Total | a+c | b+d | a+b+c+d |

Abbreviation: AEs, adverse events; a, number of reports containing both the target drug and target adverse drug reaction; b, number of reports containing other adverse drug reaction of the target drug; c, number of reports containing the target adverse drug reaction of other drugs; d, number of reports containing other drugs and other adverse drug reactions.

Supplementary Table 2:

Four major algorithms used for signal detection.

| Algorithms | Equation | Criteria |
| --- | --- | --- |
| ROR | ROR=ad/b/c | lower limit of 95% CI>1, N≥3 |
|  | 95%CI=e^ln(ROR)±1.96(1/a+1/b+1/c+1/d)^0.5^ |  |
| PRR | PRR=a(c+d)/c/(a+b) | PRR≥2, χ^2^≥4, N≥3 |
|  | χ^2^=[(ad-bc)^2](a+b+c+d)/[(a+b)(c+d)(a+c)(b+d)] |  |
| BCPNN | IC=log_2_a(a+b+c+d)(a+c)(a+b) | IC025>0 |
|  | 95%CI= E(IC) ± 2V(IC)^0.5 |  |
| MGPS | EBGM=a(a+b+c+d)/(a+c)/(a+b) | EBGM05>2 |
|  | 95%CI=e^ln(EBGM)±1.96(1/a+1/b+1/c+1/d)^0.5^ |  |

Abbreviation: a, number of reports containing both the target drug and target adverse drug reaction; b, number of reports containing other adverse drug reaction of the target drug; c, number of reports containing the target adverse drug reaction of other drugs; d, number of reports containing other drugs and other adverse drug reactions. 95%CI, 95% confidence interval; N, the number of reports; χ2, chi-squared; IC, information component; IC025, the lower limit of 95% CI of the IC; E(IC), the IC expectations; V(IC), the variance of IC; EBGM, empirical Bayesian geometric mean; EBGM05, the lower limit of 95% CI of EBGM.

Supplementary Table 3:

Signal strength of Ustekinumab AEs across System Organ Classes (SOC) in the FAERS database.

| System Organ Class (SOC) | Case numbers | ROR (95%CI) | PRR (χ^2^) | EBGM(EBGM05) | IC(IC025) |
| --- | --- | --- | --- | --- | --- |
| Injury, poisoning and procedural complications* | 13,676 | 2.23(2.19-2.28) | 1.96(6733.12) | 1.89(1.86) | 0.92(0.89) |
| Gastrointestinal disorders | 9,563 | 0.76(0.74-0.78) | 0.8(593.39) | 0.8(0.79) | -0.32(-0.35) |
| Infections and infestations* | 8,035 | 1.69(1.65-1.73) | 1.6(1831.23) | 1.56(1.53) | 0.64(0.61) |
| General disorders and administration site conditions | 7,592 | 0.62(0.6-0.63) | 0.66(1528.81) | 0.67(0.66) | -0.57(-0.6) |
| Product issues* | 3,270 | 6.99(6.72-7.27) | 6.67(12455.06) | 5.44(5.26) | 2.44(2.39) |
| Nervous system disorders* | 2,989 | 1.06(1.02-1.1) | 1.06(9.58) | 1.06(1.02) | 0.08(0.02) |
| Musculoskeletal and connective tissue disorders | 2,257 | 0.59(0.57-0.62) | 0.61(588.63) | 0.62(0.6) | -0.69(-0.76) |
| Surgical and medical procedures* | 2,201 | 1.77(1.69-1.85) | 1.74(664.32) | 1.69(1.63) | 0.76(0.7) |
| Skin and subcutaneous tissue disorders | 2,086 | 0.76(0.73-0.8) | 0.77(143.35) | 0.78(0.75) | -0.36(-0.43) |
| Investigations | 1,821 | 0.44(0.42-0.47) | 0.46(1199.13) | 0.47(0.45) | -1.08(-1.15) |
| Respiratory, thoracic and mediastinal disorders | 1,396 | 0.62(0.59-0.65) | 0.63(311.66) | 0.64(0.61) | -0.65(-0.73) |
| Neoplasms benign, malignant and unspecified (incl cysts and polyps) * | 1,104 | 1.24(1.17-1.32) | 1.24(47.84) | 1.22(1.16) | 0.29(0.2) |
| Psychiatric disorders | 858 | 0.61(0.57-0.65) | 0.61(208.09) | 0.62(0.59) | -0.68(-0.78) |
| Vascular disorders | 730 | 0.63(0.58-0.68) | 0.63(154.94) | 0.64(0.6) | -0.64(-0.75) |
| Eye disorders | 505 | 0.69(0.63-0.76) | 0.7(66.15) | 0.7(0.65) | -0.51(-0.64) |
| Metabolism and nutrition disorders | 480 | 0.51(0.47-0.56) | 0.51(218.68) | 0.52(0.49) | -0.93(-1.06) |
| Renal and urinary disorders | 454 | 0.76(0.69-0.83) | 0.76(33.59) | 0.77(0.71) | -0.38(-0.52) |
| Hepatobiliary disorders* | 429 | 1.11(1.01-1.22) | 1.11(4.54) | 1.11(1.02) | 0.14(0) |
| Immune system disorders | 420 | 0.83(0.76-0.92) | 0.83(13.53) | 0.84(0.77) | -0.25(-0.4) |
| Cardiac disorders | 414 | 0.7(0.63-0.77) | 0.7(53.21) | 0.71(0.65) | -0.5(-0.65) |
| Blood and lymphatic system disorders | 307 | 0.64(0.57-0.72) | 0.64(59.1) | 0.65(0.59) | -0.61(-0.78) |
| Reproductive system and breast disorders | 214 | 0.68(0.59-0.78) | 0.68(30.93) | 0.69(0.62) | -0.53(-0.73) |
| Pregnancy, puerperium and perinatal conditions | 200 | 0.69(0.6-0.79) | 0.69(27.04) | 0.7(0.62) | -0.52(-0.72) |
| Ear and labyrinth disorders | 133 | 0.69(0.58-0.82) | 0.69(18.4) | 0.7(0.6) | -0.52(-0.78) |
| Social circumstances | 129 | 0.59(0.49-0.7) | 0.59(36.68) | 0.6(0.52) | -0.74(-1) |
| Congenital, familial and genetic disorders* | 82 | 1.59(1.27-1.99) | 1.59(17.02) | 1.56(1.29) | 0.64(0.31) |
| Endocrine disorders | 48 | 0.69(0.52-0.92) | 0.69(6.56) | 0.7(0.55) | -0.52(-0.94) |

Abbreviation: Asterisks (*) indicate statistically significant signals in algorithm; ROR, reporting odds ratio; PRR, proportional reporting ratio; EBGM, empirical Bayesian geometric mean; EBGM05, the lower limit of the 95% CI of EBGM; IC, information component; IC025, the lower limit of the 95% CI of the IC; CI, confidence interval; AEs, adverse events.

Supplementary Table 4:

Top 100 frequency of adverse events at the PT level for Ustekinumab.

| PT | Case numbers | ROR (95%CI) | PRR (χ^2^) | EBGM(EBGM05) | IC(IC025) |
| --- | --- | --- | --- | --- | --- |
| Product dose omission issue* | 2599 | 16.77(15.94-17.63) | 16.1(22138.34) | 10.04(9.63) | 3.33(3.26) |
| Drug ineffective* | 2057 | 1.45(1.39-1.52) | 1.44(264.76) | 1.41(1.36) | 0.5(0.43) |
| Product use issue* | 1734 | 5.06(4.8-5.33) | 4.95(4556.79) | 4.27(4.09) | 2.09(2.02) |
| Inappropriate schedule of product administration* | 1106 | 2.36(2.22-2.51) | 2.34(777.84) | 2.22(2.11) | 1.15(1.06) |
| Diarrhoea | 858 | 0.83(0.78-0.89) | 0.84(27.24) | 0.84(0.79) | -0.25(-0.35) |
| Abdominal pain | 807 | 0.9(0.84-0.97) | 0.9(8.47) | 0.91(0.85) | -0.14(-0.25) |
| Headache* | 737 | 1.17(1.09-1.27) | 1.17(18.1) | 1.16(1.09) | 0.22(0.11) |
| Syringe issue* | 685 | 195.92(156.36-245.49) | 193.75(14507.2) | 22.28(18.45) | 4.48(4.33) |
| Needle issue* | 642 | 34.73(30.78-39.19) | 34.38(8568.05) | 14.74(13.32) | 3.88(3.74) |
| Fatigue | 634 | 0.7(0.64-0.75) | 0.7(81.18) | 0.71(0.66) | -0.5(-0.62) |
| Pneumonia* | 608 | 2.22(2.04-2.41) | 2.21(369.09) | 2.11(1.96) | 1.07(0.95) |
| Infusion related reaction* | 596 | 2.19(2.02-2.39) | 2.18(351.07) | 2.08(1.94) | 1.06(0.94) |
| Arthralgia | 593 | 0.72(0.66-0.78) | 0.72(61.89) | 0.73(0.68) | -0.45(-0.57) |
| Lower respiratory tract infection* | 502 | 7.52(6.8-8.31) | 7.46(2147.35) | 5.93(5.45) | 2.57(2.43) |
| Accidental exposure to product* | 502 | 28.96(25.44-32.98) | 28.74(6125.51) | 13.63(12.23) | 3.77(3.61) |
| Nausea | 464 | 0.66(0.61-0.73) | 0.67(76.3) | 0.68(0.62) | -0.57(-0.7) |
| Device issue* | 461 | 1.81(1.65-1.99) | 1.81(154.73) | 1.75(1.62) | 0.81(0.67) |
| Intestinal resection* | 423 | 3.55(3.2-3.93) | 3.53(671.01) | 3.21(2.94) | 1.68(1.53) |
| Product storage error* | 414 | 20.2(17.72-23.02) | 20.07(4090.6) | 11.39(10.21) | 3.51(3.34) |
| Nasopharyngitis | 368 | 1.03(0.93-1.14) | 1.03(0.27) | 1.03(0.94) | 0.04(-0.12) |
| Exposure during pregnancy* | 361 | 3.29(2.95-3.68) | 3.28(504.47) | 3.01(2.74) | 1.59(1.43) |
| Therapeutic response decreased* | 356 | 6.09(5.42-6.84) | 6.06(1203.06) | 5.04(4.57) | 2.33(2.17) |
| Rash | 349 | 0.97(0.87-1.08) | 0.97(0.33) | 0.97(0.89) | -0.04(-0.2) |
| Infection* | 347 | 1.86(1.67-2.07) | 1.85(127.2) | 1.79(1.64) | 0.84(0.68) |
| Dizziness | 326 | 0.93(0.83-1.04) | 0.93(1.62) | 0.93(0.85) | -0.1(-0.26) |
| Pain | 324 | 0.55(0.49-0.61) | 0.55(117.39) | 0.56(0.51) | -0.84(-1) |
| Clostridium difficile infection* | 313 | 1.97(1.75-2.21) | 1.96(136.59) | 1.89(1.71) | 0.92(0.75) |
| Dyspnoea | 312 | 0.88(0.79-0.99) | 0.89(4.48) | 0.89(0.81) | -0.17(-0.34) |
| Underdose* | 310 | 10.84(9.47-12.39) | 10.79(1901.21) | 7.76(6.93) | 2.96(2.77) |
| Intestinal obstruction | 309 | 0.89(0.8-1) | 0.89(3.78) | 0.9(0.82) | -0.16(-0.32) |
| Haematochezia | 309 | 0.59(0.53-0.66) | 0.59(85.87) | 0.6(0.55) | -0.73(-0.9) |
| Urinary tract infection* | 307 | 1.71(1.52-1.92) | 1.7(83.74) | 1.66(1.5) | 0.73(0.56) |
| Abscess* | 298 | 2.29(2.03-2.58) | 2.29(197.17) | 2.17(1.97) | 1.12(0.95) |
| Surgery* | 277 | 2.43(2.15-2.75) | 2.42(210.45) | 2.29(2.07) | 1.2(1.02) |
| Therapeutic product effect decreased* | 275 | 2.4(2.12-2.72) | 2.39(203.36) | 2.27(2.04) | 1.18(1) |
| Pruritus | 274 | 1.04(0.92-1.18) | 1.04(0.44) | 1.04(0.94) | 0.06(-0.12) |
| Vomiting | 268 | 0.58(0.52-0.66) | 0.59(77.38) | 0.6(0.54) | -0.75(-0.93) |
| Malaise | 263 | 0.44(0.39-0.5) | 0.44(184.12) | 0.45(0.41) | -1.15(-1.33) |
| Covid-19 | 257 | 0.77(0.68-0.87) | 0.77(17.29) | 0.78(0.7) | -0.36(-0.55) |
| Pyrexia | 242 | 0.47(0.42-0.54) | 0.47(139.71) | 0.48(0.44) | -1.05(-1.23) |
| General physical health deterioration* | 237 | 1.68(1.47-1.91) | 1.68(60.46) | 1.63(1.46) | 0.71(0.51) |
| Hospitalisation* | 234 | 1.61(1.41-1.84) | 1.61(50.89) | 1.57(1.41) | 0.65(0.46) |
| Sinusitis* | 228 | 1.19(1.04-1.36) | 1.19(6.77) | 1.18(1.06) | 0.24(0.05) |
| Influenza* | 227 | 1.34(1.17-1.53) | 1.34(18.38) | 1.32(1.18) | 0.4(0.2) |
| Device deployment issue* | 223 | 122.29(88.5-168.99) | 121.85(4405.41) | 20.92(15.96) | 4.39(4.13) |
| Fistula | 219 | 1.06(0.93-1.22) | 1.06(0.78) | 1.06(0.95) | 0.08(-0.11) |
| Frequent bowel movements | 216 | 0.58(0.5-0.66) | 0.58(65.97) | 0.59(0.52) | -0.77(-0.97) |
| Weight decreased | 211 | 0.41(0.36-0.47) | 0.41(175.9) | 0.42(0.38) | -1.25(-1.45) |
| Anal abscess* | 208 | 2.64(2.29-3.05) | 2.63(190.18) | 2.47(2.19) | 1.31(1.1) |
| Wrong technique in product usage process* | 200 | 1.25(1.09-1.44) | 1.25(9.61) | 1.24(1.1) | 0.31(0.1) |
| Condition aggravated | 193 | 0.23(0.2-0.26) | 0.23(505.15) | 0.24(0.21) | -2.09(-2.29) |
| Drug level decreased* | 190 | 1.22(1.06-1.41) | 1.22(7.22) | 1.21(1.07) | 0.27(0.06) |
| Colectomy* | 177 | 1.65(1.42-1.93) | 1.65(42.76) | 1.61(1.42) | 0.69(0.46) |
| Device malfunction* | 177 | 2.63(2.25-3.07) | 2.62(160.11) | 2.46(2.16) | 1.3(1.07) |
| Back pain | 175 | 0.73(0.63-0.85) | 0.73(16.45) | 0.74(0.65) | -0.43(-0.65) |
| Alopecia | 173 | 0.91(0.78-1.05) | 0.91(1.62) | 0.91(0.8) | -0.14(-0.36) |
| Abdominal pain upper | 170 | 0.59(0.51-0.69) | 0.59(46.23) | 0.6(0.53) | -0.73(-0.95) |
| Device defective* | 169 | 48.5(37.33-63.01) | 48.37(2603.45) | 16.73(13.44) | 4.06(3.78) |
| Incorrect dose administered | 168 | 0.4(0.34-0.46) | 0.4(149.25) | 0.41(0.36) | -1.29(-1.51) |
| Urticaria | 161 | 1.11(0.95-1.3) | 1.11(1.66) | 1.1(0.97) | 0.14(-0.09) |
| Migraine* | 161 | 1.45(1.24-1.7) | 1.45(21.14) | 1.42(1.25) | 0.51(0.28) |
| Sepsis* | 160 | 1.65(1.41-1.94) | 1.65(38.29) | 1.61(1.41) | 0.68(0.45) |
| Asthenia | 155 | 0.45(0.38-0.52) | 0.45(104.03) | 0.46(0.4) | -1.13(-1.36) |
| Cellulitis* | 152 | 2.34(1.98-2.77) | 2.34(106.53) | 2.22(1.93) | 1.15(0.91) |
| Constipation | 150 | 0.59(0.51-0.7) | 0.6(40.44) | 0.61(0.53) | -0.72(-0.96) |
| Hypersensitivity | 149 | 1(0.85-1.18) | 1(0) | 1(0.87) | 0(-0.24) |
| Weight increased | 148 | 0.4(0.34-0.47) | 0.4(131.43) | 0.41(0.36) | -1.29(-1.52) |
| Injection site pain | 145 | 0.2(0.17-0.23) | 0.2(467.55) | 0.21(0.18) | -2.28(-2.52) |
| Fall | 144 | 0.73(0.62-0.86) | 0.73(14.01) | 0.74(0.64) | -0.44(-0.68) |
| Anxiety | 141 | 0.7(0.59-0.82) | 0.7(17.97) | 0.71(0.61) | -0.5(-0.75) |
| Cough | 140 | 0.46(0.39-0.55) | 0.46(85.55) | 0.47(0.41) | -1.08(-1.32) |
| Nephrolithiasis | 139 | 1.14(0.96-1.35) | 1.14(2.29) | 1.13(0.98) | 0.18(-0.07) |
| Kidney infection* | 139 | 4.34(3.62-5.2) | 4.33(302.27) | 3.83(3.29) | 1.94(1.67) |
| Herpes zoster | 134 | 1.15(0.97-1.37) | 1.15(2.44) | 1.14(0.99) | 0.19(-0.06) |
| Psoriasis | 133 | 0.9(0.76-1.07) | 0.9(1.38) | 0.91(0.78) | -0.14(-0.4) |
| Chest discomfort | 133 | 1.15(0.97-1.37) | 1.15(2.6) | 1.15(0.99) | 0.2(-0.06) |
| Erythema | 130 | 0.71(0.6-0.85) | 0.72(14.32) | 0.72(0.63) | -0.47(-0.72) |
| Pain in extremity | 126 | 0.49(0.41-0.58) | 0.49(66.44) | 0.5(0.43) | -1(-1.26) |
| Product leakage* | 126 | 60.71(43.75-84.25) | 60.59(2097.94) | 17.93(13.63) | 4.16(3.83) |
| Hypertension | 123 | 0.73(0.61-0.87) | 0.73(12.26) | 0.74(0.63) | -0.44(-0.71) |
| Death | 122 | 0.65(0.54-0.78) | 0.65(22.09) | 0.66(0.57) | -0.6(-0.86) |
| Myalgia | 122 | 0.85(0.71-1.02) | 0.86(2.9) | 0.86(0.74) | -0.22(-0.48) |
| Chest pain | 115 | 0.76(0.63-0.91) | 0.76(8.52) | 0.77(0.66) | -0.38(-0.66) |
| Loss of consciousness | 115 | 1.18(0.98-1.42) | 1.18(2.97) | 1.17(1) | 0.23(-0.05) |
| Cerebrovascular accident* | 111 | 1.92(1.58-2.33) | 1.91(44.95) | 1.85(1.57) | 0.89(0.6) |
| Arthritis | 109 | 0.75(0.62-0.91) | 0.76(8.4) | 0.76(0.65) | -0.39(-0.67) |
| Bronchitis | 109 | 1.2(0.99-1.45) | 1.2(3.41) | 1.19(1.01) | 0.25(-0.03) |
| Intestinal stenosis | 107 | 0.9(0.74-1.09) | 0.9(1.12) | 0.9(0.77) | -0.14(-0.43) |
| Abdominal distension | 106 | 0.47(0.39-0.57) | 0.47(62.03) | 0.48(0.41) | -1.06(-1.34) |
| Insomnia | 105 | 0.61(0.5-0.74) | 0.61(25.29) | 0.62(0.53) | -0.69(-0.97) |
| Flushing* | 104 | 1.25(1.02-1.52) | 1.25(4.87) | 1.24(1.05) | 0.31(0.02) |
| Anaemia | 103 | 0.61(0.5-0.74) | 0.61(24.64) | 0.62(0.53) | -0.68(-0.97) |
| Hepatic enzyme increased* | 103 | 1.31(1.07-1.6) | 1.31(7.18) | 1.29(1.1) | 0.37(0.08) |
| Paraesthesia | 103 | 0.73(0.6-0.89) | 0.73(9.94) | 0.74(0.63) | -0.44(-0.72) |
| Muscle spasms | 102 | 0.59(0.48-0.72) | 0.59(28.75) | 0.6(0.51) | -0.74(-1.03) |
| Feeling abnormal | 101 | 0.62(0.51-0.76) | 0.62(22.83) | 0.63(0.53) | -0.67(-0.95) |
| Therapy non-responder* | 101 | 1.58(1.29-1.93) | 1.58(19.96) | 1.54(1.3) | 0.62(0.33) |
| Oropharyngeal pain | 99 | 0.48(0.4-0.59) | 0.48(53.35) | 0.49(0.42) | -1.01(-1.31) |
| Dehydration | 99 | 0.56(0.45-0.68) | 0.56(34.44) | 0.57(0.48) | -0.82(-1.11) |
| Seizure* | 99 | 1.99(1.62-2.45) | 1.99(45.08) | 1.91(1.61) | 0.94(0.64) |

Abbreviation: Asterisks (*) indicate statistically significant signals in algorithm; ROR, reporting odds ratio; PRR, proportional reporting ratio; EBGM, empirical Bayesian geometric mean; EBGM05, the lower limit of the 95% CI of EBGM; IC, information component; IC025, the lower limit of the 95% CI of the IC; CI, confidence interval; PT, preferred term.

Supplementary Table 5:

Top 100 frequent adverse events meeting the positive signal threshold at the PT level from FAERS data

| PT | Case numbers | ROR (95%CI) | PRR (χ^2^) | EBGM(EBGM05) | IC(IC025) |
| --- | --- | --- | --- | --- | --- |
| Product dose omission issue | 2599 | 16.77(15.94-17.63) | 16.1(22138.34) | 10.04(9.63) | 3.33(3.26) |
| Drug ineffective | 2057 | 1.45(1.39-1.52) | 1.44(264.76) | 1.41(1.36) | 0.5(0.43) |
| Product use issue | 1734 | 5.06(4.8-5.33) | 4.95(4556.79) | 4.27(4.09) | 2.09(2.02) |
| Inappropriate schedule of product administration | 1106 | 2.36(2.22-2.51) | 2.34(777.84) | 2.22(2.11) | 1.15(1.06) |
| Headache | 737 | 1.17(1.09-1.27) | 1.17(18.1) | 1.16(1.09) | 0.22(0.11) |
| Syringe issue | 685 | 195.92(156.36-245.49) | 193.75(14507.2) | 22.28(18.45) | 4.48(4.33) |
| Needle issue | 642 | 34.73(30.78-39.19) | 34.38(8568.05) | 14.74(13.32) | 3.88(3.74) |
| Pneumonia | 608 | 2.22(2.04-2.41) | 2.21(369.09) | 2.11(1.96) | 1.07(0.95) |
| Infusion related reaction | 596 | 2.19(2.02-2.39) | 2.18(351.07) | 2.08(1.94) | 1.06(0.94) |
| Lower respiratory tract infection | 502 | 7.52(6.8-8.31) | 7.46(2147.35) | 5.93(5.45) | 2.57(2.43) |
| Accidental exposure to product | 502 | 28.96(25.44-32.98) | 28.74(6125.51) | 13.63(12.23) | 3.77(3.61) |
| Device issue | 461 | 1.81(1.65-1.99) | 1.81(154.73) | 1.75(1.62) | 0.81(0.67) |
| Intestinal resection | 423 | 3.55(3.2-3.93) | 3.53(671.01) | 3.21(2.94) | 1.68(1.53) |
| Product storage error | 414 | 20.2(17.72-23.02) | 20.07(4090.6) | 11.39(10.21) | 3.51(3.34) |
| Exposure during pregnancy | 361 | 3.29(2.95-3.68) | 3.28(504.47) | 3.01(2.74) | 1.59(1.43) |
| Therapeutic response decreased | 356 | 6.09(5.42-6.84) | 6.06(1203.06) | 5.04(4.57) | 2.33(2.17) |
| Infection | 347 | 1.86(1.67-2.07) | 1.85(127.2) | 1.79(1.64) | 0.84(0.68) |
| Clostridium difficile infection | 313 | 1.97(1.75-2.21) | 1.96(136.59) | 1.89(1.71) | 0.92(0.75) |
| Underdose | 310 | 10.84(9.47-12.39) | 10.79(1901.21) | 7.76(6.93) | 2.96(2.77) |
| Urinary tract infection | 307 | 1.71(1.52-1.92) | 1.7(83.74) | 1.66(1.5) | 0.73(0.56) |
| Abscess | 298 | 2.29(2.03-2.58) | 2.29(197.17) | 2.17(1.97) | 1.12(0.95) |
| Surgery | 277 | 2.43(2.15-2.75) | 2.42(210.45) | 2.29(2.07) | 1.2(1.02) |
| Therapeutic product effect decreased | 275 | 2.4(2.12-2.72) | 2.39(203.36) | 2.27(2.04) | 1.18(1) |
| General physical health deterioration | 237 | 1.68(1.47-1.91) | 1.68(60.46) | 1.63(1.46) | 0.71(0.51) |
| Hospitalisation | 234 | 1.61(1.41-1.84) | 1.61(50.89) | 1.57(1.41) | 0.65(0.46) |
| Sinusitis | 228 | 1.19(1.04-1.36) | 1.19(6.77) | 1.18(1.06) | 0.24(0.05) |
| Influenza | 227 | 1.34(1.17-1.53) | 1.34(18.38) | 1.32(1.18) | 0.4(0.2) |
| Device deployment issue | 223 | 122.29(88.5-168.99) | 121.85(4405.41) | 20.92(15.96) | 4.39(4.13) |
| Anal abscess | 208 | 2.64(2.29-3.05) | 2.63(190.18) | 2.47(2.19) | 1.31(1.1) |
| Wrong technique in product usage process | 200 | 1.25(1.09-1.44) | 1.25(9.61) | 1.24(1.1) | 0.31(0.1) |
| Drug level decreased | 190 | 1.22(1.06-1.41) | 1.22(7.22) | 1.21(1.07) | 0.27(0.06) |
| Colectomy | 177 | 1.65(1.42-1.93) | 1.65(42.76) | 1.61(1.42) | 0.69(0.46) |
| Device malfunction | 177 | 2.63(2.25-3.07) | 2.62(160.11) | 2.46(2.16) | 1.3(1.07) |
| Device defective | 169 | 48.5(37.33-63.01) | 48.37(2603.45) | 16.73(13.44) | 4.06(3.78) |
| Migraine | 161 | 1.45(1.24-1.7) | 1.45(21.14) | 1.42(1.25) | 0.51(0.28) |
| Sepsis | 160 | 1.65(1.41-1.94) | 1.65(38.29) | 1.61(1.41) | 0.68(0.45) |
| Cellulitis | 152 | 2.34(1.98-2.77) | 2.34(106.53) | 2.22(1.93) | 1.15(0.91) |
| Kidney infection | 139 | 4.34(3.62-5.2) | 4.33(302.27) | 3.83(3.29) | 1.94(1.67) |
| Product leakage | 126 | 60.71(43.75-84.25) | 60.59(2097.94) | 17.93(13.63) | 4.16(3.83) |
| Cerebrovascular accident | 111 | 1.92(1.58-2.33) | 1.91(44.95) | 1.85(1.57) | 0.89(0.6) |
| Flushing | 104 | 1.25(1.02-1.52) | 1.25(4.87) | 1.24(1.05) | 0.31(0.02) |
| Hepatic enzyme increased | 103 | 1.31(1.07-1.6) | 1.31(7.18) | 1.29(1.1) | 0.37(0.08) |
| Therapy non-responder | 101 | 1.58(1.29-1.93) | 1.58(19.96) | 1.54(1.3) | 0.62(0.33) |
| Seizure | 99 | 1.99(1.62-2.45) | 1.99(45.08) | 1.91(1.61) | 0.94(0.64) |
| Liquid product physical issue | 98 | 12.69(9.93-16.2) | 12.67(689.85) | 8.64(7.04) | 3.11(2.77) |
| Abdominal abscess | 96 | 2.53(2.05-3.12) | 2.53(80.28) | 2.38(2) | 1.25(0.95) |
| Anaphylactic reaction | 96 | 2.52(2.04-3.11) | 2.51(79.31) | 2.37(1.99) | 1.25(0.94) |
| Drug delivery system malfunction | 95 | 31.33(23.09-42.52) | 31.29(1210.43) | 14.16(10.97) | 3.82(3.45) |
| Adverse event | 92 | 1.69(1.37-2.09) | 1.69(24.08) | 1.64(1.38) | 0.72(0.41) |
| Visual impairment | 90 | 1.33(1.08-1.65) | 1.33(6.99) | 1.31(1.1) | 0.39(0.08) |
| Syncope | 89 | 1.41(1.14-1.75) | 1.41(10.05) | 1.39(1.16) | 0.47(0.16) |
| Upper respiratory tract infection | 87 | 1.47(1.18-1.82) | 1.47(12.19) | 1.44(1.2) | 0.53(0.21) |
| Tooth abscess | 86 | 4.03(3.2-5.06) | 4.02(167.39) | 3.59(2.96) | 1.84(1.51) |
| Skin cancer | 86 | 1.86(1.49-2.31) | 1.85(31.45) | 1.79(1.49) | 0.84(0.52) |
| Device leakage | 84 | 7.99(6.24-10.23) | 7.98(385.24) | 6.24(5.08) | 2.64(2.29) |
| Gastrointestinal infection | 83 | 1.81(1.45-2.26) | 1.81(27.87) | 1.75(1.45) | 0.81(0.48) |
| Cholelithiasis | 80 | 1.95(1.55-2.45) | 1.95(34.31) | 1.88(1.55) | 0.91(0.58) |
| Therapy cessation | 80 | 4.06(3.2-5.15) | 4.06(157.77) | 3.62(2.97) | 1.85(1.51) |
| Postoperative wound infection | 79 | 3.03(2.4-3.83) | 3.03(95.41) | 2.8(2.3) | 1.49(1.15) |
| Ear infection | 79 | 1.27(1.02-1.6) | 1.27(4.42) | 1.26(1.04) | 0.33(0) |
| Respiratory tract infection | 78 | 3.35(2.64-4.25) | 3.35(112.83) | 3.06(2.51) | 1.61(1.27) |
| Post procedural infection | 78 | 2.42(1.92-3.06) | 2.42(59.24) | 2.29(1.89) | 1.2(0.86) |
| Rectal abscess | 77 | 2.57(2.03-3.26) | 2.57(66.82) | 2.42(1.99) | 1.27(0.93) |
| Ileostomy | 76 | 1.61(1.28-2.04) | 1.61(16.65) | 1.58(1.3) | 0.66(0.32) |
| Product quality issue | 76 | 3.97(3.11-5.06) | 3.96(144.62) | 3.54(2.89) | 1.83(1.47) |
| Abscess intestinal | 75 | 1.95(1.54-2.47) | 1.95(32.1) | 1.88(1.54) | 0.91(0.57) |
| Neoplasm malignant | 74 | 1.57(1.24-1.98) | 1.56(14.17) | 1.53(1.26) | 0.61(0.27) |
| Product packaging issue | 74 | 28.27(20.2-39.57) | 28.24(894.2) | 13.53(10.21) | 3.76(3.34) |
| Product label issue | 73 | 54.91(36.24-83.21) | 54.85(1176.18) | 17.41(12.3) | 4.12(3.68) |
| Foetal exposure during pregnancy | 71 | 2(1.57-2.55) | 2(32.74) | 1.92(1.57) | 0.94(0.59) |
| Diverticulitis | 63 | 1.67(1.3-2.16) | 1.67(15.98) | 1.63(1.32) | 0.7(0.33) |
| Cystitis | 63 | 1.36(1.05-1.75) | 1.36(5.64) | 1.34(1.08) | 0.42(0.05) |
| Product complaint | 62 | 57.39(36.3-90.73) | 57.33(1013.94) | 17.64(12.03) | 4.14(3.67) |
| Breast cancer | 58 | 1.96(1.5-2.56) | 1.96(25.17) | 1.89(1.51) | 0.92(0.53) |
| Small intestinal resection | 57 | 2.59(1.97-3.4) | 2.59(50.06) | 2.43(1.93) | 1.28(0.88) |
| Therapy interrupted | 57 | 1.7(1.3-2.22) | 1.7(15.22) | 1.65(1.32) | 0.72(0.33) |
| Wound infection | 55 | 2.23(1.7-2.95) | 2.23(34.3) | 2.13(1.69) | 1.09(0.69) |
| Gastroenteritis | 53 | 2.1(1.59-2.78) | 2.1(28.07) | 2.01(1.59) | 1.01(0.6) |
| Intestinal operation | 53 | 2.55(1.92-3.39) | 2.55(45.3) | 2.4(1.9) | 1.27(0.85) |
| Vertigo | 53 | 1.39(1.05-1.83) | 1.39(5.37) | 1.36(1.08) | 0.45(0.04) |
| Localised infection | 52 | 1.56(1.18-2.06) | 1.56(9.79) | 1.52(1.21) | 0.61(0.2) |
| Skin infection | 47 | 1.91(1.42-2.57) | 1.91(18.95) | 1.84(1.44) | 0.88(0.45) |
| Treatment noncompliance | 47 | 1.76(1.31-2.36) | 1.76(14.3) | 1.71(1.33) | 0.77(0.34) |
| Incorrect route of product administration | 47 | 7.29(5.26-10.11) | 7.29(195.76) | 5.83(4.43) | 2.54(2.08) |
| Gastroenteritis viral | 45 | 1.52(1.12-2.05) | 1.52(7.47) | 1.49(1.16) | 0.57(0.13) |
| Malignant melanoma | 42 | 1.55(1.13-2.11) | 1.55(7.62) | 1.51(1.17) | 0.6(0.14) |
| Device related infection | 41 | 2.69(1.95-3.72) | 2.69(39.26) | 2.52(1.93) | 1.33(0.87) |
| Transient ischaemic attack | 38 | 2.32(1.66-3.24) | 2.32(26) | 2.2(1.67) | 1.14(0.66) |
| Poor quality product administered | 38 | 10.39(7.1-15.2) | 10.38(225.02) | 7.55(5.49) | 2.92(2.39) |
| Abscess limb | 37 | 2.7(1.92-3.79) | 2.7(35.49) | 2.52(1.9) | 1.34(0.84) |
| Infected fistula | 37 | 3.57(2.53-5.05) | 3.57(59.68) | 3.24(2.43) | 1.7(1.2) |
| Product prescribing error | 37 | 2.97(2.11-4.17) | 2.97(42.91) | 2.75(2.07) | 1.46(0.97) |
| Squamous cell carcinoma | 36 | 1.84(1.31-2.58) | 1.84(12.77) | 1.78(1.34) | 0.83(0.34) |
| Tonsillitis | 36 | 2.05(1.46-2.88) | 2.05(17.87) | 1.97(1.48) | 0.98(0.48) |
| Subcutaneous abscess | 35 | 1.69(1.2-2.39) | 1.69(9.28) | 1.65(1.24) | 0.72(0.22) |
| Product administration error | 35 | 2.58(1.82-3.66) | 2.58(30.62) | 2.43(1.81) | 1.28(0.78) |
| Septic shock | 33 | 1.53(1.08-2.18) | 1.53(5.78) | 1.5(1.12) | 0.59(0.08) |
| Hepatic steatosis | 33 | 1.63(1.15-2.32) | 1.63(7.57) | 1.59(1.19) | 0.67(0.16) |
| Medication error | 32 | 2.21(1.54-3.18) | 2.21(19.44) | 2.11(1.56) | 1.08(0.55) |
| Injection related reaction | 32 | 24.83(15.15-40.69) | 24.82(359.93) | 12.72(8.41) | 3.67(3.05) |

Abbreviation: ROR, reporting odds ratio; PRR, proportional reporting ratio; EBGM, empirical Bayesian geometric mean; EBGM05, the lower limit of the 95% CI of EBGM; IC, information component; IC025, the lower limit of the 95% CI of the IC; CI, confidence interval; PT,preferred term.

Supplementary Table 6:

Top 100 most frequent adverse events for ustekinumab at the preferred term (PT) level in males from FAERS data

| PT | Case numbers | ROR (95%CI) | PRR (χ^2^) | EBGM(EBGM05) | IC(IC025) |
| --- | --- | --- | --- | --- | --- |
| Product dose omission issue* | 978 | 15.82(14.58-17.16) | 15.12(7875.41) | 9.58(8.95) | 3.26(3.15) |
| Drug ineffective* | 742 | 1.35(1.25-1.45) | 1.33(60.59) | 1.32(1.24) | 0.4(0.29) |
| Product use issue* | 631 | 4.6(4.22-5.01) | 4.49(1447.88) | 3.93(3.66) | 1.97(1.85) |
| Inappropriate schedule of product administration* | 416 | 2.84(2.57-3.15) | 2.81(435.48) | 2.61(2.4) | 1.39(1.24) |
| Diarrhoea | 285 | 0.76(0.67-0.85) | 0.76(20.84) | 0.77(0.7) | -0.38(-0.55) |
| Abdominal pain | 272 | 0.83(0.74-0.94) | 0.84(8.49) | 0.84(0.76) | -0.25(-0.43) |
| Syringe issue* | 259 | 175.49(123.27-249.85) | 173.32(5285.61) | 21.51(16.01) | 4.43(4.18) |
| Needle issue* | 244 | 29.05(24.08-35.05) | 28.72(2934.86) | 13.45(11.5) | 3.75(3.52) |
| Headache* | 203 | 1.19(1.03-1.37) | 1.18(5.58) | 1.18(1.04) | 0.23(0.03) |
| Pneumonia* | 197 | 1.94(1.68-2.25) | 1.93(82.16) | 1.86(1.65) | 0.9(0.68) |
| Accidental exposure to product* | 181 | 31.91(25.51-39.91) | 31.64(2285.78) | 14.03(11.64) | 3.81(3.54) |
| Infusion related reaction* | 175 | 2.01(1.72-2.34) | 2(80.9) | 1.92(1.69) | 0.94(0.72) |
| Fatigue | 167 | 0.58(0.5-0.68) | 0.58(48.93) | 0.59(0.52) | -0.75(-0.98) |
| Device issue* | 159 | 1.47(1.25-1.72) | 1.46(21.93) | 1.43(1.25) | 0.52(0.28) |
| Intestinal resection* | 157 | 3.01(2.55-3.55) | 2.99(185.14) | 2.77(2.41) | 1.47(1.23) |
| Product storage error* | 157 | 19.7(15.93-24.36) | 19.56(1507.68) | 11.11(9.3) | 3.47(3.2) |
| Lower respiratory tract infection* | 144 | 7.71(6.39-9.31) | 7.67(629.52) | 6.02(5.14) | 2.59(2.32) |
| Arthralgia | 141 | 0.61(0.51-0.72) | 0.61(35.13) | 0.62(0.54) | -0.69(-0.94) |
| Haematochezia | 126 | 0.58(0.48-0.69) | 0.58(37.95) | 0.59(0.51) | -0.76(-1.02) |
| Abscess* | 125 | 2.5(2.08-3.01) | 2.49(101.15) | 2.35(2.01) | 1.23(0.96) |
| Intestinal obstruction | 122 | 0.83(0.69-0.99) | 0.83(4.32) | 0.83(0.72) | -0.26(-0.53) |
| Therapeutic response decreased* | 117 | 5.29(4.33-6.47) | 5.27(330.84) | 4.49(3.79) | 2.17(1.88) |
| Clostridium difficile infection* | 114 | 1.79(1.48-2.17) | 1.79(36.93) | 1.73(1.48) | 0.79(0.51) |
| Nausea | 112 | 0.6(0.5-0.73) | 0.61(28.3) | 0.62(0.53) | -0.7(-0.98) |
| Infection* | 109 | 1.81(1.49-2.2) | 1.81(36.56) | 1.75(1.49) | 0.81(0.52) |
| Surgery* | 109 | 2.37(1.95-2.89) | 2.36(78.09) | 2.24(1.9) | 1.16(0.87) |
| Dyspnoea | 108 | 0.96(0.79-1.16) | 0.96(0.21) | 0.96(0.81) | -0.06(-0.35) |
| Covid-19 | 108 | 1.03(0.85-1.25) | 1.03(0.08) | 1.03(0.87) | 0.04(-0.24) |
| Underdose* | 107 | 15.94(12.47-20.38) | 15.86(888.8) | 9.86(8.03) | 3.3(2.97) |
| Pain | 104 | 0.58(0.48-0.7) | 0.58(30.91) | 0.59(0.5) | -0.76(-1.04) |
| Rash | 104 | 0.94(0.78-1.15) | 0.94(0.33) | 0.95(0.8) | -0.08(-0.37) |
| Dizziness | 102 | 0.93(0.76-1.14) | 0.93(0.5) | 0.93(0.79) | -0.1(-0.39) |
| Anal abscess* | 99 | 2.81(2.28-3.46) | 2.8(102.29) | 2.61(2.19) | 1.38(1.08) |
| Therapeutic product effect decreased* | 94 | 2.17(1.75-2.68) | 2.16(53.77) | 2.06(1.73) | 1.04(0.74) |
| Nasopharyngitis | 92 | 0.88(0.72-1.09) | 0.88(1.42) | 0.89(0.74) | -0.17(-0.48) |
| Malaise | 87 | 0.46(0.37-0.57) | 0.46(54.82) | 0.47(0.39) | -1.09(-1.4) |
| Wrong technique in product usage process* | 86 | 1.31(1.05-1.62) | 1.31(5.84) | 1.29(1.07) | 0.37(0.05) |
| Fistula | 81 | 0.98(0.78-1.22) | 0.98(0.05) | 0.98(0.81) | -0.03(-0.36) |
| Pyrexia | 81 | 0.43(0.34-0.53) | 0.43(60.64) | 0.44(0.37) | -1.18(-1.51) |
| Weight decreased | 80 | 0.38(0.31-0.48) | 0.38(78.78) | 0.39(0.33) | -1.35(-1.67) |
| General physical health deterioration* | 77 | 1.51(1.2-1.9) | 1.5(12.26) | 1.47(1.21) | 0.56(0.22) |
| Hospitalisation | 76 | 1.25(0.99-1.58) | 1.25(3.6) | 1.24(1.02) | 0.31(-0.03) |
| Drug level decreased* | 75 | 1.27(1.01-1.61) | 1.27(4.12) | 1.26(1.03) | 0.33(-0.01) |
| Vomiting | 73 | 0.54(0.42-0.68) | 0.54(28.61) | 0.55(0.45) | -0.87(-1.21) |
| Pruritus | 73 | 1(0.79-1.27) | 1(0) | 1(0.82) | 0(-0.34) |
| Colectomy* | 69 | 1.48(1.16-1.88) | 1.47(9.94) | 1.45(1.18) | 0.53(0.18) |
| Device malfunction* | 68 | 2.66(2.07-3.43) | 2.66(63.29) | 2.49(2.02) | 1.32(0.95) |
| Condition aggravated | 67 | 0.27(0.22-0.35) | 0.28(127.1) | 0.28(0.23) | -1.81(-2.17) |
| Frequent bowel movements | 67 | 0.41(0.32-0.52) | 0.41(55.43) | 0.42(0.35) | -1.24(-1.6) |
| Influenza | 67 | 1.19(0.93-1.52) | 1.19(1.97) | 1.18(0.96) | 0.24(-0.12) |
| Device deployment issue* | 65 | 72.72(44.45-118.96) | 72.5(1119.32) | 18.46(12.23) | 4.21(3.73) |
| Back pain | 64 | 0.82(0.64-1.06) | 0.82(2.36) | 0.83(0.67) | -0.27(-0.63) |
| Death | 61 | 0.66(0.51-0.85) | 0.66(10.4) | 0.67(0.54) | -0.58(-0.95) |
| Incorrect dose administered | 58 | 0.37(0.28-0.48) | 0.37(61.31) | 0.38(0.31) | -1.39(-1.77) |
| Device defective* | 58 | 35.85(23.81-53.98) | 35.75(775.61) | 14.75(10.48) | 3.88(3.41) |
| Asthenia | 57 | 0.45(0.35-0.59) | 0.45(37.29) | 0.46(0.37) | -1.11(-1.49) |
| Sinusitis | 57 | 1.2(0.92-1.57) | 1.2(1.8) | 1.19(0.95) | 0.25(-0.14) |
| Sepsis | 55 | 1.32(1-1.73) | 1.32(3.96) | 1.3(1.03) | 0.38(-0.02) |
| Nephrolithiasis | 54 | 0.95(0.72-1.25) | 0.95(0.14) | 0.95(0.76) | -0.07(-0.47) |
| Abdominal pain upper | 54 | 0.57(0.43-0.75) | 0.57(17.07) | 0.58(0.46) | -0.78(-1.18) |
| Urinary tract infection* | 53 | 1.67(1.26-2.21) | 1.67(13.27) | 1.62(1.29) | 0.7(0.29) |
| Cellulitis* | 51 | 2.19(1.64-2.92) | 2.19(30.11) | 2.09(1.64) | 1.06(0.64) |
| Cough | 50 | 0.53(0.4-0.7) | 0.53(20.42) | 0.54(0.43) | -0.89(-1.3) |
| Fall | 49 | 0.77(0.58-1.03) | 0.77(3.16) | 0.78(0.62) | -0.36(-0.77) |
| Constipation | 49 | 0.65(0.49-0.87) | 0.66(8.67) | 0.66(0.52) | -0.59(-1) |
| Urticaria* | 49 | 1.38(1.04-1.85) | 1.38(4.91) | 1.36(1.07) | 0.45(0.03) |
| Anxiety | 49 | 0.83(0.63-1.11) | 0.83(1.59) | 0.84(0.66) | -0.25(-0.67) |
| Cerebrovascular accident* | 47 | 1.84(1.37-2.48) | 1.84(16.66) | 1.78(1.39) | 0.83(0.4) |
| Intestinal stenosis | 46 | 0.94(0.7-1.27) | 0.94(0.15) | 0.94(0.74) | -0.08(-0.51) |
| Inflammation | 46 | 0.99(0.73-1.32) | 0.99(0.01) | 0.99(0.77) | -0.02(-0.45) |
| Drug level below therapeutic* | 45 | 1.49(1.1-2.01) | 1.49(6.8) | 1.46(1.13) | 0.55(0.11) |
| Chest discomfort | 44 | 1.29(0.95-1.74) | 1.29(2.67) | 1.27(0.99) | 0.35(-0.1) |
| Liquid product physical issue* | 44 | 13.24(9.15-19.16) | 13.21(317.62) | 8.81(6.46) | 3.14(2.64) |
| Abdominal abscess* | 43 | 2.52(1.84-3.45) | 2.52(35.56) | 2.37(1.82) | 1.25(0.79) |
| Weight increased | 43 | 0.37(0.27-0.5) | 0.37(45.99) | 0.38(0.29) | -1.4(-1.84) |
| Product leakage* | 43 | 67.28(37.37-121.12) | 67.14(724.67) | 18.11(11.07) | 4.18(3.6) |
| Therapy non-responder* | 42 | 1.82(1.33-2.49) | 1.81(14.28) | 1.76(1.35) | 0.81(0.36) |
| Injection site pain | 41 | 0.18(0.13-0.24) | 0.18(157.86) | 0.18(0.14) | -2.45(-2.9) |
| Syncope* | 40 | 1.76(1.28-2.43) | 1.76(12.17) | 1.7(1.3) | 0.77(0.3) |
| Small intestinal obstruction | 39 | 0.79(0.57-1.09) | 0.79(2.08) | 0.8(0.61) | -0.32(-0.79) |
| Anal fistula | 39 | 0.83(0.61-1.15) | 0.83(1.24) | 0.84(0.64) | -0.25(-0.72) |
| Loss of consciousness | 39 | 0.97(0.71-1.34) | 0.97(0.03) | 0.97(0.74) | -0.04(-0.5) |
| Chest pain | 39 | 0.74(0.54-1.02) | 0.74(3.51) | 0.75(0.57) | -0.42(-0.89) |
| Pain in extremity | 38 | 0.51(0.37-0.7) | 0.51(17.38) | 0.52(0.4) | -0.94(-1.4) |
| Anaemia | 38 | 0.63(0.45-0.87) | 0.63(8.2) | 0.64(0.49) | -0.65(-1.12) |
| Hypertension | 37 | 0.76(0.55-1.06) | 0.76(2.68) | 0.77(0.58) | -0.38(-0.85) |
| Rectal haemorrhage | 36 | 0.41(0.29-0.57) | 0.41(30.32) | 0.42(0.32) | -1.25(-1.73) |
| Skin cancer* | 36 | 1.62(1.16-2.28) | 1.62(8.03) | 1.58(1.19) | 0.66(0.17) |
| Seizure* | 35 | 1.63(1.16-2.3) | 1.63(7.97) | 1.59(1.19) | 0.67(0.17) |
| Post procedural infection* | 35 | 2.86(2.01-4.06) | 2.86(37.66) | 2.65(1.98) | 1.41(0.9) |
| Erythema | 35 | 0.69(0.49-0.96) | 0.69(4.81) | 0.7(0.53) | -0.52(-1.01) |
| Gastrointestinal infection* | 35 | 1.86(1.32-2.62) | 1.85(12.78) | 1.79(1.34) | 0.84(0.34) |
| Drug delivery system malfunction* | 35 | 41.06(23.7-71.13) | 40.99(496.57) | 15.54(9.81) | 3.96(3.34) |
| Product packaging issue* | 34 | 41.98(23.94-73.61) | 41.91(486.82) | 15.67(9.79) | 3.97(3.34) |
| Visual impairment* | 34 | 1.64(1.16-2.32) | 1.64(7.85) | 1.59(1.19) | 0.67(0.17) |
| Hypersensitivity | 34 | 1.06(0.75-1.5) | 1.06(0.13) | 1.06(0.8) | 0.09(-0.41) |
| Dehydration | 33 | 0.51(0.36-0.73) | 0.51(14.83) | 0.53(0.39) | -0.93(-1.43) |
| Insomnia | 33 | 0.54(0.38-0.76) | 0.54(12.79) | 0.55(0.41) | -0.87(-1.37) |
| Myalgia | 33 | 0.72(0.51-1.02) | 0.72(3.4) | 0.73(0.55) | -0.45(-0.95) |
| Postoperative wound infection* | 32 | 3.51(2.42-5.08) | 3.5(49.79) | 3.18(2.33) | 1.67(1.13) |

Abbreviation: Asterisks (*) indicate statistically significant signals in algorithm; ROR, reporting odds ratio; PRR, proportional reporting ratio; EBGM, empirical Bayesian geometric mean; EBGM05, the lower limit of the 95% CI of EBGM; IC, information component; IC025, the lower limit of the 95% CI of the IC; CI, confidence interval; PT, preferred term; AEs, adverse events.

Supplementary Table 7:

Top 100 most frequent adverse events for ustekinumab at the PT level in females from FAERS data

| PT | Case numbers | ROR (95%CI) | PRR (χ^2^) | EBGM(EBGM05) | IC(IC025) |
| --- | --- | --- | --- | --- | --- |
| Product dose omission issue* | 1463 | 17.43(16.27-18.67) | 16.77(12334.89) | 9.93(9.37) | 3.31(3.22) |
| Drug ineffective* | 1128 | 1.44(1.36-1.53) | 1.43(139.51) | 1.4(1.33) | 0.49(0.4) |
| Product use issue* | 977 | 5.27(4.91-5.66) | 5.16(2666.25) | 4.36(4.11) | 2.13(2.02) |
| Inappropriate schedule of product administration* | 617 | 3.6(3.3-3.92) | 3.55(978.67) | 3.2(2.97) | 1.68(1.55) |
| Diarrhoea | 549 | 0.82(0.76-0.9) | 0.83(19.34) | 0.83(0.78) | -0.26(-0.39) |
| Abdominal pain | 504 | 0.89(0.81-0.97) | 0.89(7.11) | 0.89(0.83) | -0.17(-0.3) |
| Headache* | 479 | 1.14(1.04-1.25) | 1.14(7.94) | 1.13(1.05) | 0.18(0.04) |
| Fatigue | 429 | 0.74(0.67-0.82) | 0.75(36.27) | 0.75(0.7) | -0.41(-0.55) |
| Syringe issue* | 377 | 181.35(133.51-246.35) | 179.48(7280.39) | 20.41(15.8) | 4.35(4.15) |
| Arthralgia | 374 | 0.76(0.69-0.84) | 0.76(26.89) | 0.77(0.71) | -0.37(-0.53) |
| Infusion related reaction* | 371 | 2.18(1.96-2.42) | 2.16(212.68) | 2.06(1.88) | 1.04(0.89) |
| Pneumonia* | 360 | 2.27(2.04-2.53) | 2.26(229.89) | 2.14(1.95) | 1.1(0.94) |
| Exposure during pregnancy* | 352 | 3.09(2.76-3.46) | 3.07(433.02) | 2.82(2.57) | 1.49(1.33) |
| Needle issue* | 351 | 33.3(28.21-39.31) | 32.99(4347.26) | 13.76(11.98) | 3.78(3.59) |
| Lower respiratory tract infection* | 347 | 8.09(7.15-9.16) | 8.02(1564.02) | 6.14(5.54) | 2.62(2.44) |
| Nausea | 325 | 0.67(0.6-0.75) | 0.67(51.26) | 0.68(0.62) | -0.55(-0.72) |
| Accidental exposure to product* | 304 | 28.2(23.79-33.43) | 27.97(3474.21) | 12.84(11.14) | 3.68(3.48) |
| Device issue* | 268 | 1.69(1.49-1.92) | 1.69(69.86) | 1.64(1.48) | 0.71(0.53) |
| Nasopharyngitis* | 263 | 1.2(1.06-1.35) | 1.19(7.91) | 1.18(1.07) | 0.24(0.06) |
| Product storage error* | 252 | 20.42(17.2-24.25) | 20.29(2400.42) | 11.01(9.54) | 3.46(3.24) |
| Intestinal resection* | 238 | 3.52(3.07-4.04) | 3.51(368.51) | 3.16(2.82) | 1.66(1.46) |
| Urinary tract infection* | 230 | 1.62(1.41-1.85) | 1.61(50.09) | 1.57(1.4) | 0.65(0.45) |
| Pain | 213 | 0.55(0.48-0.63) | 0.55(75.25) | 0.57(0.5) | -0.82(-1.02) |
| Dizziness | 208 | 0.95(0.83-1.09) | 0.95(0.49) | 0.95(0.85) | -0.07(-0.27) |
| Rash | 202 | 0.89(0.78-1.03) | 0.89(2.48) | 0.9(0.8) | -0.16(-0.36) |
| Infection* | 189 | 1.77(1.53-2.06) | 1.77(58.8) | 1.71(1.51) | 0.78(0.56) |
| Dyspnoea | 187 | 0.87(0.75-1.01) | 0.87(3.55) | 0.87(0.77) | -0.19(-0.41) |
| Pruritus* | 184 | 1.17(1.01-1.36) | 1.17(4.4) | 1.16(1.03) | 0.22(0) |
| Vomiting | 182 | 0.58(0.5-0.67) | 0.58(53.01) | 0.59(0.53) | -0.75(-0.97) |
| Underdose* | 180 | 12.65(10.53-15.2) | 12.59(1220.51) | 8.36(7.17) | 3.06(2.81) |
| Haematochezia | 174 | 0.6(0.52-0.7) | 0.6(44.6) | 0.61(0.54) | -0.7(-0.93) |
| Intestinal obstruction | 172 | 0.85(0.73-0.99) | 0.85(4.25) | 0.86(0.76) | -0.22(-0.44) |
| Therapeutic response decreased* | 170 | 6.22(5.24-7.38) | 6.19(577.79) | 5.05(4.38) | 2.34(2.09) |
| Malaise | 169 | 0.48(0.41-0.56) | 0.48(91.89) | 0.5(0.44) | -1.01(-1.24) |
| Clostridium difficile infection* | 163 | 1.91(1.63-2.24) | 1.9(64.51) | 1.83(1.6) | 0.87(0.64) |
| Therapeutic product effect decreased* | 161 | 2.53(2.15-2.98) | 2.53(133.29) | 2.37(2.07) | 1.24(1.01) |
| Sinusitis | 160 | 1.16(0.99-1.36) | 1.16(3.48) | 1.15(1.01) | 0.21(-0.03) |
| General physical health deterioration* | 159 | 1.81(1.54-2.13) | 1.81(53.45) | 1.75(1.53) | 0.81(0.57) |
| Abscess* | 155 | 2.44(2.06-2.88) | 2.43(117.8) | 2.29(1.99) | 1.19(0.95) |
| Influenza* | 150 | 1.54(1.3-1.82) | 1.54(26.27) | 1.5(1.31) | 0.59(0.34) |
| Pyrexia | 143 | 0.48(0.41-0.57) | 0.49(76.38) | 0.5(0.43) | -1.01(-1.25) |
| Alopecia | 143 | 0.86(0.73-1.02) | 0.86(3.08) | 0.87(0.75) | -0.21(-0.45) |
| Frequent bowel movements | 142 | 0.66(0.56-0.79) | 0.67(23.31) | 0.68(0.59) | -0.57(-0.81) |
| Surgery* | 139 | 2.11(1.77-2.51) | 2.1(73.3) | 2(1.73) | 1(0.75) |
| Covid-19 | 138 | 0.8(0.67-0.95) | 0.8(6.77) | 0.81(0.7) | -0.31(-0.56) |
| Migraine* | 135 | 1.57(1.32-1.87) | 1.57(25.89) | 1.53(1.32) | 0.61(0.36) |
| Hospitalisation* | 129 | 1.51(1.26-1.81) | 1.51(20.78) | 1.48(1.27) | 0.56(0.3) |
| Condition aggravated | 124 | 0.39(0.33-0.47) | 0.39(114.14) | 0.41(0.35) | -1.3(-1.56) |
| Fistula | 123 | 1.15(0.96-1.38) | 1.15(2.33) | 1.14(0.98) | 0.19(-0.07) |
| Weight decreased | 122 | 0.49(0.41-0.58) | 0.49(64.06) | 0.5(0.43) | -1(-1.26) |
| Abdominal pain upper | 112 | 0.6(0.5-0.72) | 0.6(29.04) | 0.61(0.52) | -0.71(-0.98) |
| Kidney infection* | 112 | 4.41(3.6-5.41) | 4.4(245.51) | 3.83(3.23) | 1.94(1.64) |
| Device deployment issue* | 105 | 121.37(74.45-197.88) | 121.03(1915.4) | 19.39(12.88) | 4.28(3.9) |
| Weight increased | 102 | 0.55(0.45-0.67) | 0.55(36.3) | 0.56(0.48) | -0.83(-1.12) |
| Back pain | 102 | 0.68(0.56-0.83) | 0.68(14.41) | 0.69(0.59) | -0.53(-0.82) |
| Urticaria | 100 | 1.02(0.83-1.24) | 1.02(0.03) | 1.02(0.86) | 0.02(-0.27) |
| Hypersensitivity | 99 | 0.96(0.79-1.18) | 0.96(0.14) | 0.96(0.81) | -0.05(-0.35) |
| Device defective* | 99 | 50.56(35.34-72.33) | 50.42(1452.45) | 15.97(11.83) | 4(3.62) |
| Device malfunction* | 98 | 2.2(1.78-2.7) | 2.19(57.84) | 2.08(1.75) | 1.06(0.76) |
| Drug level decreased* | 98 | 1.63(1.33-2) | 1.63(22.16) | 1.58(1.33) | 0.66(0.36) |
| Wrong technique in product usage process | 97 | 0.96(0.79-1.18) | 0.96(0.13) | 0.96(0.81) | -0.05(-0.35) |
| Constipation | 97 | 0.57(0.46-0.7) | 0.57(30.87) | 0.58(0.49) | -0.78(-1.08) |
| Cellulitis* | 95 | 2.53(2.05-3.13) | 2.53(78.74) | 2.37(1.98) | 1.24(0.93) |
| Fall | 95 | 0.72(0.59-0.89) | 0.72(9.63) | 0.73(0.62) | -0.45(-0.75) |
| Anal abscess* | 92 | 2.57(2.07-3.19) | 2.57(78.83) | 2.4(2) | 1.26(0.95) |
| Injection site pain | 92 | 0.17(0.14-0.21) | 0.17(366.91) | 0.18(0.15) | -2.48(-2.78) |
| Incorrect dose administered | 91 | 0.38(0.31-0.47) | 0.39(88.4) | 0.4(0.33) | -1.34(-1.64) |
| Colectomy* | 90 | 1.69(1.36-2.1) | 1.69(23.52) | 1.64(1.37) | 0.71(0.4) |
| Asthenia | 90 | 0.44(0.36-0.54) | 0.44(62.7) | 0.45(0.38) | -1.14(-1.45) |
| Erythema | 90 | 0.85(0.68-1.04) | 0.85(2.44) | 0.85(0.71) | -0.23(-0.54) |
| Pain in extremity | 86 | 0.49(0.4-0.61) | 0.49(44.05) | 0.5(0.42) | -0.99(-1.3) |
| Anxiety | 86 | 0.64(0.52-0.8) | 0.65(16.33) | 0.66(0.55) | -0.61(-0.92) |
| Bronchitis* | 85 | 1.3(1.05-1.62) | 1.3(5.59) | 1.28(1.07) | 0.36(0.04) |
| Cough | 85 | 0.48(0.39-0.6) | 0.48(45.93) | 0.5(0.41) | -1.01(-1.33) |
| Sepsis* | 84 | 1.58(1.27-1.97) | 1.58(16.73) | 1.54(1.28) | 0.62(0.3) |
| Hypertension* | 84 | 0.83(0.67-1.04) | 0.83(2.68) | 0.84(0.7) | -0.25(-0.57) |
| Chest discomfort | 84 | 1.24(1-1.55) | 1.24(3.71) | 1.23(1.02) | 0.3(-0.03) |
| Psoriasis | 82 | 0.83(0.66-1.03) | 0.83(2.79) | 0.84(0.69) | -0.26(-0.58) |
| Nephrolithiasis | 79 | 1.32(1.05-1.65) | 1.32(5.65) | 1.3(1.07) | 0.38(0.04) |
| Herpes zoster | 77 | 1.15(0.92-1.45) | 1.15(1.48) | 1.15(0.95) | 0.2(-0.14) |
| Arthritis | 76 | 0.77(0.61-0.97) | 0.77(5.1) | 0.78(0.64) | -0.36(-0.7) |
| Oropharyngeal pain | 74 | 0.59(0.47-0.75) | 0.59(20.31) | 0.6(0.5) | -0.73(-1.07) |
| Myalgia | 74 | 0.84(0.67-1.06) | 0.84(2.1) | 0.85(0.7) | -0.24(-0.58) |
| Muscle spasms | 74 | 0.68(0.54-0.86) | 0.68(10.69) | 0.69(0.57) | -0.53(-0.87) |
| Paraesthesia | 73 | 0.78(0.62-0.99) | 0.79(4.15) | 0.79(0.65) | -0.34(-0.68) |
| Loss of consciousness | 73 | 1.27(1-1.6) | 1.26(3.83) | 1.25(1.03) | 0.32(-0.02) |
| Product leakage* | 73 | 69.65(43.58-111.3) | 69.51(1181.09) | 17.41(11.76) | 4.12(3.68) |
| Feeling abnormal | 73 | 0.72(0.57-0.91) | 0.72(7.88) | 0.73(0.6) | -0.46(-0.8) |
| Chest pain | 72 | 0.79(0.63-1) | 0.79(3.76) | 0.8(0.66) | -0.32(-0.67) |
| Stress | 71 | 0.68(0.54-0.86) | 0.68(10.17) | 0.69(0.57) | -0.53(-0.88) |
| Abdominal distension | 71 | 0.49(0.39-0.62) | 0.49(37.1) | 0.5(0.41) | -1(-1.34) |
| Insomnia | 68 | 0.65(0.51-0.83) | 0.65(12.35) | 0.66(0.54) | -0.6(-0.95) |
| Hepatic enzyme increased | 67 | 1.26(0.99-1.61) | 1.26(3.42) | 1.25(1.01) | 0.32(-0.04) |
| Flushing* | 67 | 1.46(1.14-1.87) | 1.46(8.97) | 1.43(1.16) | 0.51(0.15) |
| Abdominal discomfort | 67 | 0.65(0.51-0.83) | 0.65(12.13) | 0.66(0.54) | -0.6(-0.95) |
| Abortion spontaneous | 66 | 1(0.78-1.28) | 1(0) | 1(0.81) | 0(-0.36) |
| Dehydration | 61 | 0.53(0.41-0.69) | 0.53(24.33) | 0.54(0.44) | -0.88(-1.25) |
| Peripheral swelling | 60 | 0.59(0.46-0.77) | 0.59(16.37) | 0.6(0.49) | -0.73(-1.1) |
| Haemorrhage | 60 | 0.69(0.54-0.9) | 0.7(7.79) | 0.7(0.57) | -0.51(-0.88) |
| Anaphylactic reaction* | 59 | 2.6(1.98-3.4) | 2.59(51.73) | 2.43(1.94) | 1.28(0.89) |

Abbreviation: Asterisks (*) indicate statistically significant signals in algorithm; ROR, reporting odds ratio; PRR, proportional reporting ratio; EBGM, empirical Bayesian geometric mean; EBGM05, the lower limit of the 95% CI of EBGM; IC, information component; IC025, the lower limit of the 95% CI of the IC; CI, confidence interval; PT, preferred term; AEs, adverse events.

Supplementary Table 8:

Top 100 most frequent adverse events at the PT level for ustekinumab in patients aged under 18 from FAERS data

| PT | Case numbers | ROR (95%CI) | PRR (χ^2^) | EBGM(EBGM05) | IC(IC025) |
| --- | --- | --- | --- | --- | --- |
| Product use issue* | 189 | 3.33(2.84-3.89) | 3.12(250.71) | 2.89(2.54) | 1.53(1.3) |
| Product dose omission issue* | 107 | 31.62(23.86-41.89) | 30.07(1403.03) | 14.52(11.47) | 3.86(3.51) |
| Drug ineffective | 51 | 0.96(0.73-1.28) | 0.97(0.06) | 0.97(0.76) | -0.05(-0.46) |
| Infusion related reaction* | 47 | 1.58(1.17-2.12) | 1.56(9.12) | 1.53(1.19) | 0.61(0.18) |
| Inappropriate schedule of product administration* | 32 | 4.53(3.1-6.6) | 4.47(73.94) | 3.96(2.89) | 1.99(1.45) |
| Abdominal pain | 28 | 0.58(0.4-0.85) | 0.59(8.23) | 0.59(0.43) | -0.75(-1.3) |
| Needle issue* | 27 | 26.46(15.49-45.19) | 26.13(326.62) | 13.57(8.67) | 3.76(3.09) |
| Syringe issue* | 26 | 343.95(81.58-1450.14) | 339.73(627.52) | 25.19(7.56) | 4.66(3.88) |
| Accidental exposure to product* | 24 | 45.3(23.4-87.7) | 44.8(378.89) | 17.14(9.86) | 4.1(3.36) |
| Headache | 23 | 1.19(0.78-1.81) | 1.19(0.67) | 1.18(0.83) | 0.24(-0.36) |
| Device issue* | 22 | 1.62(1.05-2.49) | 1.61(4.83) | 1.58(1.1) | 0.66(0.03) |
| Fatigue | 18 | 0.86(0.54-1.38) | 0.86(0.38) | 0.87(0.58) | -0.21(-0.88) |
| Haematochezia | 17 | 0.37(0.23-0.6) | 0.37(17.91) | 0.38(0.26) | -1.38(-2.07) |
| Dyspnoea | 16 | 1.07(0.65-1.77) | 1.07(0.07) | 1.07(0.7) | 0.09(-0.62) |
| Underdose* | 15 | 26.31(12.85-53.9) | 26.13(181.38) | 13.57(7.45) | 3.76(2.87) |
| Clostridium difficile infection | 14 | 1.38(0.8-2.36) | 1.38(1.37) | 1.36(0.86) | 0.44(-0.33) |
| Anaphylactic reaction* | 14 | 2.11(1.22-3.64) | 2.1(7.52) | 2.02(1.28) | 1.01(0.24) |
| Product storage error* | 13 | 85.45(27.84-262.3) | 84.93(253.79) | 20.75(8.12) | 4.37(3.35) |
| Arthralgia | 13 | 0.93(0.53-1.62) | 0.93(0.07) | 0.93(0.59) | -0.1(-0.89) |
| Diarrhoea | 13 | 0.41(0.23-0.7) | 0.41(11.01) | 0.42(0.26) | -1.25(-2.03) |
| Vomiting | 12 | 0.51(0.29-0.91) | 0.51(5.46) | 0.52(0.32) | -0.93(-1.74) |
| Foetal exposure during pregnancy | 12 | 1.53(0.85-2.75) | 1.53(2.09) | 1.5(0.92) | 0.59(-0.24) |
| Device malfunction* | 11 | 4.66(2.45-8.85) | 4.64(26.68) | 4.09(2.39) | 2.03(1.13) |
| Nausea | 11 | 0.52(0.28-0.94) | 0.52(4.89) | 0.53(0.32) | -0.92(-1.77) |
| Device deployment issue* | 10 | 52.51(17.93-153.76) | 52.27(167.66) | 18.09(7.36) | 4.18(3.05) |
| Pyrexia | 10 | 0.39(0.21-0.72) | 0.39(9.61) | 0.4(0.24) | -1.33(-2.21) |
| Hypersensitivity* | 10 | 2.03(1.07-3.87) | 2.03(4.83) | 1.95(1.14) | 0.96(0.06) |
| Drug level decreased | 10 | 0.75(0.4-1.41) | 0.75(0.79) | 0.76(0.45) | -0.4(-1.28) |
| Injection site pain | 10 | 0.27(0.15-0.51) | 0.28(19.12) | 0.28(0.17) | -1.82(-2.7) |
| Therapeutic response decreased* | 10 | 2.28(1.19-4.35) | 2.27(6.57) | 2.17(1.26) | 1.12(0.21) |
| Device defective* | 10 | 52.51(17.93-153.76) | 52.27(167.66) | 18.09(7.36) | 4.18(3.05) |
| Therapeutic product effect decreased | 9 | 1.67(0.85-3.28) | 1.67(2.27) | 1.63(0.93) | 0.7(-0.24) |
| Surgery* | 9 | 4.92(2.41-10.03) | 4.9(23.55) | 4.28(2.36) | 2.1(1.11) |
| Flushing | 9 | 1.87(0.95-3.68) | 1.87(3.39) | 1.81(1.03) | 0.86(-0.09) |
| Hospitalisation | 8 | 0.98(0.48-1.98) | 0.98(0) | 0.98(0.54) | -0.03(-1.02) |
| Product leakage* | 8 | 209.86(26.23-1678.69) | 209.06(184.09) | 24.12(4.23) | 4.59(3.26) |
| Malaise | 8 | 0.62(0.31-1.25) | 0.62(1.85) | 0.63(0.35) | -0.67(-1.65) |
| Colectomy | 7 | 1.83(0.85-3.95) | 1.83(2.46) | 1.78(0.93) | 0.83(-0.23) |
| Abscess | 7 | 1.74(0.81-3.75) | 1.74(2.08) | 1.7(0.89) | 0.76(-0.3) |
| Erythema | 7 | 1.16(0.54-2.47) | 1.16(0.14) | 1.15(0.61) | 0.2(-0.84) |
| Intestinal resection | 7 | 1.71(0.8-3.68) | 1.71(1.94) | 1.67(0.88) | 0.74(-0.32) |
| Rash | 7 | 0.6(0.29-1.28) | 0.61(1.77) | 0.61(0.33) | -0.7(-1.74) |
| Condition aggravated | 7 | 0.64(0.3-1.35) | 0.64(1.42) | 0.65(0.34) | -0.63(-1.67) |
| Drug delivery system malfunction* | 7 | 183.54(22.57-1492.48) | 182.93(158.34) | 23.74(4.11) | 4.57(3.17) |
| Wrong technique in product usage process | 7 | 0.66(0.31-1.39) | 0.66(1.22) | 0.67(0.36) | -0.59(-1.62) |
| Chest discomfort | 7 | 1.64(0.76-3.51) | 1.63(1.62) | 1.6(0.84) | 0.67(-0.38) |
| Incorrect dose administered | 6 | 0.3(0.13-0.67) | 0.3(9.72) | 0.31(0.16) | -1.69(-2.79) |
| Weight decreased | 6 | 0.43(0.19-0.97) | 0.43(4.38) | 0.44(0.23) | -1.17(-2.28) |
| Anal abscess | 6 | 0.87(0.39-1.97) | 0.87(0.11) | 0.88(0.44) | -0.19(-1.3) |
| Infection | 6 | 1.57(0.69-3.58) | 1.57(1.17) | 1.54(0.77) | 0.62(-0.51) |
| Pruritus | 6 | 1.02(0.45-2.32) | 1.02(0) | 1.02(0.52) | 0.03(-1.08) |
| General physical health deterioration | 6 | 0.28(0.13-0.63) | 0.28(10.89) | 0.29(0.15) | -1.78(-2.88) |
| Psoriasis | 6 | 0.55(0.24-1.23) | 0.55(2.17) | 0.56(0.28) | -0.84(-1.94) |
| Product prescribing error* | 6 | 11.23(4.31-29.25) | 11.2(39.03) | 8.14(3.65) | 3.03(1.76) |
| Anxiety | 6 | 0.83(0.37-1.87) | 0.83(0.2) | 0.83(0.42) | -0.26(-1.37) |
| Cough | 6 | 0.64(0.28-1.43) | 0.64(1.21) | 0.65(0.33) | -0.63(-1.74) |
| Asthenia | 5 | 0.7(0.29-1.71) | 0.7(0.62) | 0.71(0.34) | -0.49(-1.69) |
| Muscle spasms | 5 | 1.36(0.55-3.35) | 1.36(0.46) | 1.34(0.63) | 0.43(-0.79) |
| Product use in unapproved indication | 5 | 1.05(0.43-2.58) | 1.05(0.01) | 1.05(0.5) | 0.07(-1.13) |
| Alopecia | 5 | 1.18(0.48-2.89) | 1.18(0.13) | 1.17(0.55) | 0.23(-0.98) |
| Rectal haemorrhage | 5 | 0.62(0.25-1.5) | 0.62(1.17) | 0.63(0.3) | -0.68(-1.87) |
| Nephrolithiasis* | 5 | 3.12(1.23-7.88) | 3.11(6.41) | 2.89(1.33) | 1.53(0.28) |
| Nasopharyngitis | 5 | 0.53(0.22-1.28) | 0.53(2.09) | 0.54(0.26) | -0.9(-2.09) |
| Treatment failure | 5 | 1.33(0.54-3.28) | 1.33(0.4) | 1.32(0.62) | 0.4(-0.82) |
| Oropharyngeal pain | 5 | 0.73(0.3-1.79) | 0.73(0.47) | 0.74(0.35) | -0.43(-1.63) |
| Dizziness | 5 | 0.62(0.26-1.52) | 0.63(1.1) | 0.63(0.3) | -0.66(-1.85) |
| Drug level below therapeutic | 5 | 1.02(0.42-2.5) | 1.02(0) | 1.02(0.48) | 0.03(-1.18) |
| Pain | 5 | 0.41(0.17-1) | 0.41(4.12) | 0.42(0.2) | -1.24(-2.43) |
| Ileostomy | 4 | 2.49(0.89-6.96) | 2.49(3.26) | 2.36(1) | 1.24(-0.12) |
| Myalgia | 4 | 1.31(0.48-3.57) | 1.31(0.27) | 1.29(0.56) | 0.37(-0.96) |
| Abscess intestinal* | 4 | 2.99(1.06-8.42) | 2.99(4.75) | 2.78(1.17) | 1.48(0.11) |
| Back pain | 4 | 0.95(0.35-2.58) | 0.95(0.01) | 0.95(0.41) | -0.07(-1.39) |
| Fistula | 4 | 0.63(0.23-1.71) | 0.63(0.83) | 0.64(0.28) | -0.64(-1.95) |
| Appendicitis* | 4 | 2.75(0.98-7.72) | 2.75(4.04) | 2.58(1.09) | 1.37(0.01) |
| Constipation | 4 | 0.59(0.22-1.6) | 0.59(1.09) | 0.6(0.26) | -0.73(-2.04) |
| Gastrointestinal disorder | 4 | 1.58(0.58-4.35) | 1.58(0.81) | 1.55(0.67) | 0.63(-0.7) |
| Injection site haemorrhage | 4 | 0.29(0.11-0.78) | 0.29(6.76) | 0.3(0.13) | -1.73(-3.03) |
| Therapeutic product effect incomplete | 4 | 1.19(0.44-3.24) | 1.19(0.11) | 1.18(0.51) | 0.24(-1.09) |
| Inflammation | 4 | 0.92(0.34-2.51) | 0.93(0.02) | 0.93(0.4) | -0.11(-1.43) |
| Pneumonia | 4 | 0.6(0.22-1.61) | 0.6(1.06) | 0.61(0.26) | -0.72(-2.03) |
| Feeling abnormal | 4 | 1.29(0.47-3.53) | 1.29(0.25) | 1.28(0.55) | 0.35(-0.98) |
| Product administered to patient of inappropriate age* | 4 | 2.91(1.03-8.18) | 2.9(4.5) | 2.71(1.14) | 1.44(0.07) |
| Adverse drug reaction* | 4 | 3.17(1.12-8.96) | 3.17(5.3) | 2.93(1.23) | 1.55(0.18) |
| Faecal calprotectin increased | 4 | 1.26(0.46-3.44) | 1.26(0.2) | 1.25(0.54) | 0.32(-1.01) |
| Paraesthesia | 3 | 0.97(0.31-3.07) | 0.97(0) | 0.97(0.37) | -0.05(-1.52) |
| Drug ineffective for unapproved indication | 3 | 1.87(0.58-6.03) | 1.87(1.13) | 1.81(0.68) | 0.86(-0.65) |
| Urinary tract infection | 3 | 1.24(0.39-3.97) | 1.24(0.14) | 1.23(0.47) | 0.3(-1.18) |
| Dysphagia* | 3 | 3.92(1.17-13.22) | 3.92(5.68) | 3.54(1.28) | 1.82(0.26) |
| Abdominal discomfort | 3 | 0.87(0.28-2.75) | 0.87(0.06) | 0.88(0.33) | -0.19(-1.67) |
| Hot flush* | 3 | 3.92(1.17-13.22) | 3.92(5.68) | 3.54(1.28) | 1.82(0.26) |
| Influenza | 3 | 0.55(0.17-1.72) | 0.55(1.1) | 0.56(0.21) | -0.84(-2.31) |
| Abdominal abscess | 3 | 1.51(0.47-4.83) | 1.51(0.49) | 1.48(0.56) | 0.57(-0.93) |
| Frequent bowel movements | 3 | 0.17(0.05-0.53) | 0.17(12.06) | 0.18(0.07) | -2.5(-3.95) |
| Weight increased | 3 | 0.45(0.14-1.4) | 0.45(2.01) | 0.46(0.18) | -1.13(-2.59) |
| Injection site bruising | 3 | 0.87(0.28-2.75) | 0.87(0.06) | 0.88(0.33) | -0.19(-1.67) |
| Arthritis | 3 | 1.78(0.55-5.75) | 1.78(0.96) | 1.73(0.65) | 0.79(-0.71) |
| Premature baby | 3 | 0.96(0.3-3.03) | 0.96(0.01) | 0.96(0.36) | -0.06(-1.54) |
| Urticaria | 3 | 0.52(0.16-1.62) | 0.52(1.34) | 0.53(0.2) | -0.93(-2.39) |
| Acne | 3 | 1.06(0.33-3.36) | 1.06(0.01) | 1.06(0.4) | 0.08(-1.4) |
| Disseminated tuberculosis* | 3 | 3.27(0.98-10.87) | 3.27(4.2) | 3.01(1.1) | 1.59(0.05) |

Abbreviation: Asterisks (*) indicate statistically significant signals in algorithm; ROR, reporting odds ratio; PRR, proportional reporting ratio; EBGM, empirical Bayesian geometric mean; EBGM05, the lower limit of the 95% CI of EBGM; IC, information component; IC025, the lower limit of the 95% CI of the IC; CI, confidence interval; PT, preferred term.

Supplementary Table 9:

Top 100 most frequent adverse events for ustekinumab at the PT level in patients aged 18 to 65 from FAERS data

| PT | Case numbers | ROR (95%CI) | PRR (χ^2^) | EBGM(EBGM05) | IC(IC025) |
| --- | --- | --- | --- | --- | --- |
| Product dose omission issue* | 1519 | 18.23(17.04-19.5) | 17.41(13584.41) | 10.44(9.87) | 3.38(3.3) |
| Product use issue* | 961 | 5.85(5.45-6.28) | 5.7(3020.94) | 4.79(4.51) | 2.26(2.16) |
| Drug ineffective | 706 | 1.05(0.97-1.13) | 1.05(1.54) | 1.05(0.98) | 0.07(-0.05) |
| Inappropriate schedule of product administration* | 590 | 2.64(2.43-2.88) | 2.61(532.56) | 2.45(2.28) | 1.29(1.17) |
| Abdominal pain | 510 | 0.96(0.88-1.05) | 0.96(0.68) | 0.97(0.9) | -0.05(-0.18) |
| Diarrhoea | 453 | 0.8(0.73-0.88) | 0.8(21.31) | 0.81(0.75) | -0.3(-0.44) |
| Syringe issue* | 414 | 202.02(150.21-271.69) | 199.42(8655.09) | 22(17.17) | 4.46(4.26) |
| Headache | 385 | 1.05(0.94-1.16) | 1.05(0.74) | 1.04(0.96) | 0.06(-0.09) |
| Fatigue | 366 | 0.73(0.65-0.81) | 0.73(36.53) | 0.74(0.67) | -0.44(-0.59) |
| Infusion related reaction* | 366 | 2.51(2.25-2.8) | 2.5(298.04) | 2.35(2.15) | 1.23(1.08) |
| Needle issue* | 355 | 32.96(28.06-38.71) | 32.6(4570.33) | 14.27(12.47) | 3.84(3.64) |
| Accidental exposure to product* | 328 | 31.15(26.42-36.73) | 30.84(4108.88) | 13.94(12.14) | 3.8(3.6) |
| Pneumonia* | 292 | 2.25(1.99-2.53) | 2.23(182.62) | 2.13(1.92) | 1.09(0.91) |
| Device issue* | 282 | 1.56(1.38-1.76) | 1.56(52.85) | 1.52(1.37) | 0.61(0.43) |
| Nausea | 275 | 0.65(0.58-0.73) | 0.65(49.51) | 0.66(0.6) | -0.59(-0.77) |
| Arthralgia | 269 | 0.63(0.56-0.72) | 0.64(54.49) | 0.65(0.58) | -0.63(-0.81) |
| Intestinal resection* | 248 | 3.45(3.02-3.94) | 3.43(373.53) | 3.12(2.79) | 1.64(1.45) |
| Product storage error* | 244 | 29.6(24.52-35.74) | 29.38(2981.65) | 13.64(11.65) | 3.77(3.54) |
| Lower respiratory tract infection* | 222 | 6.4(5.52-7.43) | 6.37(791.98) | 5.23(4.62) | 2.39(2.17) |
| Nasopharyngitis | 208 | 1.03(0.9-1.18) | 1.03(0.16) | 1.03(0.91) | 0.04(-0.17) |
| Exposure during pregnancy* | 202 | 2.86(2.47-3.31) | 2.84(216.17) | 2.65(2.34) | 1.4(1.19) |
| Haematochezia | 191 | 0.66(0.57-0.76) | 0.66(31.86) | 0.67(0.6) | -0.57(-0.78) |
| Dizziness | 186 | 0.93(0.8-1.08) | 0.93(0.97) | 0.93(0.82) | -0.1(-0.32) |
| General physical health deterioration* | 182 | 2.5(2.15-2.92) | 2.5(147.95) | 2.35(2.07) | 1.23(1.01) |
| Dyspnoea | 175 | 0.93(0.8-1.09) | 0.93(0.79) | 0.94(0.83) | -0.09(-0.32) |
| Intestinal obstruction | 172 | 1.01(0.87-1.18) | 1.01(0.04) | 1.01(0.89) | 0.02(-0.2) |
| Rash | 165 | 0.88(0.76-1.03) | 0.89(2.37) | 0.89(0.78) | -0.17(-0.4) |
| Vomiting | 160 | 0.58(0.5-0.68) | 0.59(46.15) | 0.6(0.52) | -0.75(-0.98) |
| Covid-19 | 159 | 0.96(0.82-1.13) | 0.96(0.25) | 0.96(0.84) | -0.06(-0.29) |
| Underdose* | 158 | 10.86(8.99-13.11) | 10.81(965.31) | 7.73(6.6) | 2.95(2.69) |
| Pain | 157 | 0.51(0.43-0.6) | 0.51(72.32) | 0.52(0.46) | -0.94(-1.17) |
| Pyrexia | 148 | 0.49(0.42-0.58) | 0.49(76.88) | 0.5(0.44) | -0.99(-1.23) |
| Sinusitis* | 147 | 1.28(1.08-1.51) | 1.28(8.36) | 1.26(1.1) | 0.34(0.09) |
| Infection* | 146 | 1.63(1.38-1.93) | 1.63(33.36) | 1.59(1.38) | 0.67(0.42) |
| Frequent bowel movements | 145 | 0.69(0.58-0.81) | 0.69(19.97) | 0.7(0.61) | -0.52(-0.76) |
| Abscess* | 143 | 2.04(1.72-2.42) | 2.03(69.41) | 1.95(1.69) | 0.97(0.71) |
| Pruritus | 143 | 1.05(0.89-1.24) | 1.05(0.35) | 1.05(0.91) | 0.07(-0.18) |
| Clostridium difficile infection* | 141 | 1.76(1.48-2.09) | 1.75(42.68) | 1.7(1.47) | 0.77(0.52) |
| Influenza* | 141 | 1.54(1.3-1.82) | 1.54(24.82) | 1.5(1.3) | 0.59(0.34) |
| Therapeutic response decreased* | 141 | 5.7(4.74-6.85) | 5.68(438.57) | 4.77(4.09) | 2.25(1.99) |
| Malaise | 139 | 0.46(0.39-0.55) | 0.47(84.6) | 0.48(0.41) | -1.07(-1.32) |
| Anal abscess* | 136 | 3.13(2.62-3.75) | 3.13(173.86) | 2.88(2.48) | 1.52(1.26) |
| Urinary tract infection* | 124 | 1.42(1.19-1.7) | 1.42(14.53) | 1.4(1.2) | 0.48(0.22) |
| Device malfunction* | 121 | 2.54(2.11-3.07) | 2.54(102.04) | 2.39(2.04) | 1.26(0.98) |
| Surgery* | 114 | 2.36(1.95-2.86) | 2.36(81.04) | 2.23(1.9) | 1.16(0.88) |
| Weight decreased | 112 | 0.43(0.35-0.52) | 0.43(84.03) | 0.44(0.38) | -1.19(-1.46) |
| Therapeutic product effect decreased* | 109 | 2.39(1.96-2.91) | 2.39(79.86) | 2.26(1.92) | 1.18(0.89) |
| Hospitalisation* | 107 | 1.84(1.51-2.25) | 1.84(38.19) | 1.78(1.51) | 0.83(0.54) |
| Wrong technique in product usage process | 107 | 1.02(0.84-1.24) | 1.02(0.05) | 1.02(0.87) | 0.03(-0.25) |
| Fistula | 102 | 1(0.82-1.22) | 1(0) | 1(0.84) | 0(-0.29) |
| Device deployment issue* | 101 | 140.67(84.14-235.18) | 140.23(2011.75) | 21.06(13.7) | 4.4(4.01) |
| Migraine* | 99 | 1.41(1.15-1.72) | 1.4(10.9) | 1.38(1.17) | 0.47(0.17) |
| Alopecia | 97 | 0.98(0.8-1.2) | 0.98(0.03) | 0.98(0.83) | -0.03(-0.32) |
| Incorrect dose administered | 95 | 0.35(0.29-0.43) | 0.35(112.1) | 0.36(0.31) | -1.46(-1.76) |
| Chest discomfort* | 94 | 1.4(1.14-1.72) | 1.4(10.12) | 1.38(1.16) | 0.46(0.16) |
| Back pain | 91 | 0.73(0.59-0.89) | 0.73(9.14) | 0.73(0.62) | -0.45(-0.75) |
| Colectomy* | 89 | 1.7(1.37-2.11) | 1.7(23.87) | 1.65(1.38) | 0.72(0.41) |
| Abdominal pain upper | 89 | 0.57(0.46-0.7) | 0.57(28.68) | 0.58(0.48) | -0.79(-1.1) |
| Urticaria | 89 | 1.06(0.86-1.31) | 1.06(0.27) | 1.06(0.88) | 0.08(-0.23) |
| Erythema | 86 | 0.91(0.74-1.14) | 0.92(0.65) | 0.92(0.77) | -0.12(-0.44) |
| Condition aggravated | 84 | 0.21(0.17-0.26) | 0.21(254.66) | 0.21(0.18) | -2.22(-2.53) |
| Product use in unapproved indication* | 83 | 1.59(1.27-1.99) | 1.59(16.94) | 1.55(1.29) | 0.63(0.31) |
| Cough | 82 | 0.49(0.39-0.61) | 0.49(43.25) | 0.5(0.42) | -1(-1.32) |
| Product leakage* | 82 | 88.2(55.08-141.22) | 87.97(1491.71) | 19.4(13.08) | 4.28(3.85) |
| Drug level decreased* | 82 | 1.32(1.06-1.65) | 1.32(6.04) | 1.3(1.08) | 0.38(0.06) |
| Weight increased | 80 | 0.41(0.33-0.51) | 0.41(67.85) | 0.42(0.35) | -1.26(-1.58) |
| Hypersensitivity | 80 | 1.03(0.82-1.29) | 1.03(0.06) | 1.03(0.85) | 0.04(-0.29) |
| Injection site pain | 78 | 0.16(0.13-0.2) | 0.16(333.86) | 0.17(0.14) | -2.56(-2.88) |
| Device defective* | 75 | 40.32(27.79-58.52) | 40.23(1061.07) | 15.51(11.35) | 3.95(3.53) |
| Asthenia | 74 | 0.43(0.34-0.54) | 0.43(55.48) | 0.44(0.36) | -1.19(-1.52) |
| Pain in extremity | 74 | 0.53(0.42-0.66) | 0.53(30.49) | 0.54(0.44) | -0.89(-1.23) |
| Anxiety | 73 | 0.64(0.51-0.81) | 0.64(14.05) | 0.65(0.54) | -0.61(-0.95) |
| Cellulitis* | 72 | 2.18(1.71-2.77) | 2.17(41.8) | 2.07(1.69) | 1.05(0.7) |
| Constipation | 72 | 0.59(0.47-0.74) | 0.59(20.16) | 0.6(0.49) | -0.74(-1.08) |
| Chest pain | 71 | 0.81(0.64-1.03) | 0.81(3) | 0.82(0.67) | -0.29(-0.64) |
| Nephrolithiasis | 69 | 1.09(0.85-1.38) | 1.09(0.44) | 1.08(0.88) | 0.11(-0.24) |
| Sepsis* | 68 | 1.39(1.09-1.78) | 1.39(7.14) | 1.37(1.12) | 0.46(0.1) |
| Liquid product physical issue* | 68 | 14.11(10.44-19.05) | 14.08(517.62) | 9.19(7.15) | 3.2(2.79) |
| Flushing* | 68 | 1.39(1.09-1.77) | 1.39(6.92) | 1.36(1.11) | 0.45(0.09) |
| Hypertension | 67 | 0.82(0.64-1.04) | 0.82(2.61) | 0.82(0.67) | -0.28(-0.63) |
| Loss of consciousness* | 67 | 1.31(1.03-1.68) | 1.31(4.7) | 1.29(1.05) | 0.37(0.01) |
| Myalgia | 66 | 0.81(0.64-1.04) | 0.82(2.68) | 0.82(0.67) | -0.28(-0.64) |
| Psoriasis | 65 | 0.98(0.77-1.26) | 0.98(0.02) | 0.98(0.8) | -0.03(-0.39) |
| Abdominal abscess* | 62 | 2.75(2.11-3.58) | 2.75(61.68) | 2.56(2.06) | 1.36(0.98) |
| Abdominal distension | 62 | 0.49(0.38-0.63) | 0.49(32.32) | 0.5(0.41) | -1(-1.37) |
| Paraesthesia | 62 | 0.71(0.55-0.91) | 0.71(7.13) | 0.72(0.58) | -0.48(-0.85) |
| Kidney infection* | 60 | 3.37(2.57-4.42) | 3.36(87.29) | 3.07(2.45) | 1.62(1.23) |
| Feeling abnormal | 59 | 0.67(0.52-0.87) | 0.67(9.34) | 0.68(0.55) | -0.56(-0.94) |
| Hepatic enzyme increased* | 57 | 1.4(1.07-1.83) | 1.4(6.16) | 1.38(1.1) | 0.46(0.07) |
| Bronchitis | 56 | 1.1(0.84-1.44) | 1.1(0.49) | 1.1(0.88) | 0.13(-0.26) |
| Influenza like illness | 56 | 0.87(0.67-1.14) | 0.87(0.99) | 0.88(0.7) | -0.19(-0.58) |
| Oropharyngeal pain | 55 | 0.45(0.34-0.58) | 0.45(37.26) | 0.46(0.37) | -1.13(-1.52) |
| Stress | 54 | 0.63(0.48-0.83) | 0.63(11.23) | 0.64(0.51) | -0.64(-1.03) |
| Dehydration | 54 | 0.57(0.43-0.74) | 0.57(17.26) | 0.58(0.46) | -0.79(-1.18) |
| Fall | 54 | 0.71(0.54-0.93) | 0.71(6.3) | 0.72(0.57) | -0.48(-0.88) |
| Drug delivery system malfunction* | 54 | 29.69(19.89-44.32) | 29.64(662.56) | 13.7(9.79) | 3.78(3.29) |
| Arthritis | 53 | 0.74(0.56-0.97) | 0.74(4.62) | 0.75(0.6) | -0.42(-0.81) |
| Rectal haemorrhage | 53 | 0.43(0.33-0.56) | 0.43(39.71) | 0.44(0.35) | -1.19(-1.58) |
| Postoperative wound infection* | 53 | 3.53(2.64-4.71) | 3.52(83.4) | 3.2(2.51) | 1.68(1.26) |
| Muscle spasms | 53 | 0.56(0.43-0.74) | 0.56(17.9) | 0.57(0.45) | -0.81(-1.21) |

Abbreviation: Asterisks (*) indicate statistically significant signals in algorithm; ROR, reporting odds ratio; PRR, proportional reporting ratio; EBGM, empirical Bayesian geometric mean; EBGM05, the lower limit of the 95% CI of EBGM; IC, information component; IC025, the lower limit of the 95% CI of the IC; CI, confidence interval; PT, preferred term.

Supplementary Table 10:

Top 100 most frequent adverse events for ustekinumab at the PT level in patients aged over 65 from FAERS data

| PT | Case numbers | ROR (95%CI) | PRR (χ^2^) | EBGM(EBGM05) | IC(IC025) |
| --- | --- | --- | --- | --- | --- |
| Product dose omission issue* | 274 | 21.1(18.01-24.72) | 20.17(2883.08) | 12.03(10.54) | 3.59(3.38) |
| Product use issue* | 133 | 6.07(5.02-7.33) | 5.96(452.26) | 5.07(4.33) | 2.34(2.07) |
| Drug ineffective | 124 | 1.15(0.96-1.38) | 1.15(2.34) | 1.14(0.98) | 0.19(-0.07) |
| Diarrhoea | 117 | 0.91(0.76-1.1) | 0.91(0.97) | 0.92(0.78) | -0.13(-0.4) |
| Inappropriate schedule of product administration* | 110 | 3.31(2.71-4.04) | 3.27(155.72) | 3.03(2.56) | 1.6(1.31) |
| Pneumonia* | 80 | 1.61(1.28-2.01) | 1.6(17.03) | 1.56(1.29) | 0.65(0.31) |
| Abdominal pain | 73 | 0.95(0.75-1.2) | 0.95(0.21) | 0.95(0.78) | -0.08(-0.42) |
| Needle issue* | 67 | 28.46(20.22-40.08) | 28.16(864.8) | 14.37(10.79) | 3.85(3.41) |
| Syringe issue* | 62 | 171.13(87.7-333.9) | 169.36(1441.8) | 24.38(13.94) | 4.61(4.11) |
| Lower respiratory tract infection* | 61 | 10.58(7.86-14.23) | 10.48(378.53) | 7.85(6.13) | 2.97(2.56) |
| Fall | 58 | 1.06(0.81-1.37) | 1.05(0.16) | 1.05(0.84) | 0.07(-0.31) |
| Fatigue | 56 | 0.69(0.53-0.91) | 0.7(7.32) | 0.7(0.56) | -0.51(-0.89) |
| Accidental exposure to product | 56 | 39.58(26.28-59.62) | 39.22(856.79) | 16.69(11.85) | 4.06(3.58) |
| Headache | 52 | 1.08(0.82-1.42) | 1.08(0.27) | 1.07(0.85) | 0.1(-0.3) |
| Arthralgia | 49 | 0.74(0.56-0.98) | 0.74(4.31) | 0.75(0.59) | -0.42(-0.83) |
| Urinary tract infection* | 47 | 1.44(1.08-1.94) | 1.44(6.06) | 1.42(1.11) | 0.5(0.08) |
| Device issue* | 45 | 1.72(1.27-2.33) | 1.72(12.73) | 1.67(1.3) | 0.74(0.3) |
| Infusion related reaction* | 44 | 2.15(1.58-2.93) | 2.14(24.94) | 2.06(1.59) | 1.04(0.59) |
| Product storage error* | 41 | 28.91(18.63-44.85) | 28.72(534.94) | 14.51(10.05) | 3.86(3.31) |
| Nausea | 40 | 0.66(0.48-0.9) | 0.66(6.84) | 0.67(0.51) | -0.58(-1.04) |
| Rash* | 39 | 1.45(1.05-2) | 1.45(5.15) | 1.42(1.09) | 0.51(0.04) |
| Dizziness | 38 | 0.98(0.71-1.36) | 0.98(0.01) | 0.98(0.75) | -0.03(-0.5) |
| Intestinal obstruction | 36 | 1.01(0.72-1.4) | 1.01(0) | 1.01(0.76) | 0.01(-0.48) |
| Underdose* | 35 | 19.23(12.47-29.64) | 19.12(353.75) | 11.66(8.12) | 3.54(2.97) |
| Death | 34 | 0.61(0.43-0.85) | 0.61(8.45) | 0.62(0.46) | -0.7(-1.19) |
| Clostridium difficile infection* | 33 | 1.82(1.28-2.59) | 1.81(11.33) | 1.76(1.31) | 0.82(0.31) |
| Dyspnoea | 32 | 0.83(0.58-1.18) | 0.83(1.09) | 0.84(0.62) | -0.26(-0.77) |
| Pruritus | 29 | 1.22(0.84-1.77) | 1.21(1.06) | 1.21(0.88) | 0.27(-0.27) |
| Pain | 28 | 0.51(0.35-0.74) | 0.51(12.99) | 0.52(0.38) | -0.94(-1.49) |
| Infection* | 28 | 1.93(1.32-2.84) | 1.93(11.68) | 1.87(1.35) | 0.9(0.34) |
| Nasopharyngitis | 26 | 1.05(0.71-1.55) | 1.05(0.06) | 1.05(0.75) | 0.07(-0.5) |
| Cellulitis* | 25 | 3.1(2.05-4.69) | 3.09(31.8) | 2.88(2.03) | 1.52(0.93) |
| Covid-19 | 25 | 0.74(0.5-1.1) | 0.74(2.21) | 0.75(0.54) | -0.42(-0.99) |
| Hypertension | 24 | 0.98(0.65-1.48) | 0.98(0.01) | 0.98(0.7) | -0.03(-0.61) |
| Frequent bowel movements | 23 | 0.57(0.37-0.86) | 0.57(7.48) | 0.58(0.41) | -0.8(-1.39) |
| Haematochezia | 23 | 0.51(0.34-0.78) | 0.52(10.34) | 0.52(0.37) | -0.93(-1.53) |
| Cerebrovascular accident | 23 | 1.5(0.99-2.29) | 1.5(3.68) | 1.48(1.04) | 0.56(-0.04) |
| Malaise | 23 | 0.39(0.26-0.59) | 0.4(21.1) | 0.4(0.29) | -1.31(-1.9) |
| Kidney infection* | 23 | 5.84(3.72-9.16) | 5.82(75.7) | 4.97(3.41) | 2.31(1.67) |
| Therapeutic product effect decreased* | 23 | 3.46(2.24-5.35) | 3.45(35.61) | 3.18(2.21) | 1.67(1.04) |
| Back pain | 22 | 0.75(0.49-1.14) | 0.75(1.8) | 0.76(0.53) | -0.4(-1.01) |
| Diverticulitis* | 22 | 2.64(1.7-4.1) | 2.64(20.4) | 2.49(1.73) | 1.32(0.69) |
| Influenza | 22 | 1.52(0.99-2.34) | 1.52(3.73) | 1.49(1.04) | 0.58(-0.04) |
| Weight decreased | 22 | 0.39(0.25-0.59) | 0.39(20.77) | 0.4(0.28) | -1.32(-1.93) |
| Intestinal resection* | 22 | 3.33(2.14-5.19) | 3.32(31.84) | 3.07(2.12) | 1.62(0.98) |
| Pyrexia | 21 | 0.51(0.33-0.79) | 0.51(9.62) | 0.52(0.36) | -0.94(-1.56) |
| Sepsis | 21 | 1.53(0.98-2.37) | 1.53(3.61) | 1.5(1.04) | 0.58(-0.05) |
| Asthenia | 21 | 0.39(0.25-0.6) | 0.39(19.78) | 0.4(0.28) | -1.32(-1.94) |
| Nephrolithiasis | 21 | 1.52(0.98-2.37) | 1.52(3.56) | 1.49(1.03) | 0.58(-0.05) |
| Anaemia | 21 | 0.93(0.6-1.44) | 0.93(0.11) | 0.93(0.65) | -0.1(-0.73) |
| Wrong technique in product usage process | 21 | 1.07(0.69-1.65) | 1.07(0.09) | 1.07(0.74) | 0.09(-0.54) |
| Confusional state* | 20 | 1.77(1.13-2.79) | 1.77(6.28) | 1.72(1.18) | 0.78(0.13) |
| Incorrect dose administered | 20 | 0.55(0.35-0.86) | 0.55(7.23) | 0.56(0.39) | -0.84(-1.47) |
| Constipation | 20 | 0.57(0.36-0.88) | 0.57(6.53) | 0.58(0.4) | -0.8(-1.43) |
| Product label issue* | 20 | 109.63(41.13-292.2) | 109.26(429.15) | 22.65(9.97) | 4.5(3.65) |
| General physical health deterioration* | 20 | 2.01(1.28-3.17) | 2.01(9.45) | 1.94(1.33) | 0.96(0.3) |
| Vomiting | 20 | 0.53(0.34-0.82) | 0.53(8.26) | 0.54(0.37) | -0.89(-1.53) |
| Sinusitis | 20 | 1.45(0.92-2.27) | 1.45(2.61) | 1.42(0.98) | 0.51(-0.14) |
| Pain in extremity | 20 | 0.67(0.43-1.05) | 0.68(3.06) | 0.68(0.47) | -0.55(-1.19) |
| Herpes zoster | 19 | 1.29(0.81-2.05) | 1.29(1.19) | 1.28(0.87) | 0.35(-0.31) |
| Device defective | 19 | 86.77(34.64-217.35) | 86.5(385.43) | 21.52(9.98) | 4.43(3.57) |
| Device malfunction* | 18 | 4.18(2.54-6.86) | 4.17(37.62) | 3.75(2.47) | 1.91(1.2) |
| Hospitalisation | 18 | 1.05(0.66-1.69) | 1.05(0.05) | 1.05(0.71) | 0.07(-0.6) |
| Neoplasm malignant* | 18 | 2.68(1.65-4.35) | 2.67(17.18) | 2.52(1.68) | 1.34(0.64) |
| Skin cancer* | 17 | 1.77(1.08-2.89) | 1.77(5.31) | 1.72(1.14) | 0.78(0.08) |
| Cataract | 17 | 0.94(0.58-1.53) | 0.94(0.06) | 0.94(0.63) | -0.08(-0.78) |
| Surgery* | 17 | 2.42(1.47-3.98) | 2.42(13.01) | 2.3(1.52) | 1.2(0.49) |
| Therapeutic response decreased* | 17 | 4.75(2.84-7.95) | 4.74(42.76) | 4.19(2.72) | 2.07(1.33) |
| Bronchitis | 16 | 1.5(0.91-2.48) | 1.5(2.51) | 1.47(0.96) | 0.56(-0.16) |
| Localised infection* | 16 | 3.68(2.18-6.21) | 3.67(27.45) | 3.36(2.17) | 1.75(1) |
| Gastrointestinal inflammation | 16 | 1.64(0.99-2.73) | 1.64(3.8) | 1.61(1.05) | 0.68(-0.04) |
| Device deployment issue* | 16 | 73.04(28.57-186.71) | 72.84(309.23) | 20.59(9.39) | 4.36(3.44) |
| Visual impairment* | 15 | 2.25(1.33-3.82) | 2.25(9.65) | 2.16(1.39) | 1.11(0.36) |
| Transient ischaemic attack* | 14 | 2.74(1.58-4.74) | 2.73(13.98) | 2.57(1.62) | 1.36(0.58) |
| Contusion | 14 | 1.26(0.74-2.15) | 1.26(0.71) | 1.25(0.8) | 0.32(-0.45) |
| Abscess* | 14 | 3.42(1.96-5.96) | 3.41(21.26) | 3.15(1.98) | 1.65(0.87) |
| Urticaria* | 14 | 1.92(1.12-3.31) | 1.92(5.79) | 1.86(1.18) | 0.9(0.12) |
| Cough | 14 | 0.46(0.27-0.78) | 0.46(8.82) | 0.47(0.3) | -1.1(-1.85) |
| Loss of consciousness | 13 | 0.93(0.54-1.62) | 0.93(0.06) | 0.93(0.59) | -0.1(-0.88) |
| Product leakage* | 13 | 71.17(25.37-199.71) | 71.02(249.32) | 20.45(8.63) | 4.35(3.34) |
| Lung neoplasm malignant* | 13 | 1.91(1.09-3.36) | 1.91(5.27) | 1.85(1.15) | 0.89(0.09) |
| Condition aggravated | 13 | 0.22(0.13-0.37) | 0.22(36.43) | 0.22(0.14) | -2.15(-2.93) |
| Alopecia | 13 | 1.06(0.61-1.85) | 1.06(0.04) | 1.06(0.67) | 0.08(-0.71) |
| Abdominal pain upper | 13 | 0.49(0.28-0.84) | 0.49(6.96) | 0.5(0.31) | -1.01(-1.79) |
| Muscle spasms | 13 | 0.8(0.46-1.39) | 0.8(0.63) | 0.81(0.51) | -0.31(-1.1) |
| Device leakage* | 13 | 14.83(7.55-29.13) | 14.8(108.49) | 9.95(5.65) | 3.31(2.41) |
| Dehydration | 12 | 0.49(0.28-0.86) | 0.49(6.31) | 0.5(0.31) | -1.01(-1.81) |
| Liquid product physical issue* | 12 | 10.26(5.28-19.93) | 10.24(72.81) | 7.72(4.43) | 2.95(2.04) |
| Drug level decreased | 12 | 1.05(0.59-1.88) | 1.05(0.03) | 1.05(0.65) | 0.07(-0.74) |
| Atrial fibrillation | 12 | 0.91(0.51-1.62) | 0.91(0.1) | 0.91(0.56) | -0.13(-0.95) |
| Arthritis | 11 | 0.66(0.36-1.21) | 0.66(1.82) | 0.67(0.41) | -0.57(-1.42) |
| Decreased appetite | 11 | 0.45(0.25-0.82) | 0.45(7.23) | 0.46(0.28) | -1.12(-1.96) |
| Hypotension | 11 | 0.77(0.42-1.4) | 0.77(0.76) | 0.77(0.47) | -0.37(-1.22) |
| Abdominal distension | 11 | 0.54(0.3-0.98) | 0.54(4.18) | 0.55(0.33) | -0.86(-1.7) |
| Cholelithiasis* | 11 | 2.35(1.27-4.35) | 2.35(7.84) | 2.24(1.34) | 1.16(0.3) |
| Weight increased | 11 | 0.42(0.23-0.76) | 0.42(8.7) | 0.43(0.26) | -1.22(-2.06) |
| Balance disorder | 11 | 1.35(0.74-2.48) | 1.35(0.97) | 1.34(0.81) | 0.42(-0.44) |
| Product complaint* | 11 | 150.51(33.35-679.21) | 150.24(250.89) | 23.96(6.79) | 4.58(3.45) |
| Anxiety | 10 | 0.74(0.39-1.38) | 0.74(0.91) | 0.75(0.44) | -0.42(-1.31) |
| Rash pruritic | 10 | 1.18(0.63-2.23) | 1.18(0.27) | 1.17(0.69) | 0.23(-0.66) |

Abbreviation: Asterisks (*) indicate statistically significant signals in algorithm; ROR, reporting odds ratio; PRR, proportional reporting ratio; EBGM, empirical Bayesian geometric mean; EBGM05, the lower limit of the 95% CI of EBGM; IC, information component; IC025, the lower limit of the 95% CI of the IC; CI, confidence interval; PT, preferred term.

Supplementary Table 11:

Top 100 most frequent adverse events for ustekinumab at the PT level in patients with non-serious outcomes from FAERS data

| PT | Case numbers | ROR (95%CI) | PRR (χ^2^) | EBGM(EBGM05) | IC(IC025) |
| --- | --- | --- | --- | --- | --- |
| Product dose omission issue* | 2772 | 30.72(29.35-32.16) | 26.54(51402.67) | 20.13(19.24) | 4.33(4.26) |
| Drug ineffective* | 992 | 2.27(2.12-2.42) | 2.2(648.38) | 2.17(2.03) | 1.12(1.02) |
| Inappropriate schedule of product administration* | 655 | 3.56(3.29-3.86) | 3.48(1119.06) | 3.37(3.12) | 1.75(1.63) |
| Syringe issue* | 597 | 266.83(225.97-315.09) | 258.77(35983.02) | 61.48(52.06) | 5.94(5.64) |
| Needle issue* | 572 | 84.78(75.35-95.38) | 82.34(22568.38) | 40.91(36.36) | 5.35(5.11) |
| Accidental exposure to product* | 446 | 70.81(62.32-80.47) | 69.23(16024.17) | 37.43(32.94) | 5.23(4.95) |
| Device issue* | 408 | 5.26(4.75-5.82) | 5.17(1294) | 4.92(4.44) | 2.3(2.13) |
| Product storage error* | 357 | 29.59(26.19-33.44) | 29.07(7087.86) | 21.54(19.07) | 4.43(4.17) |
| Product use issue* | 321 | 2.61(2.33-2.92) | 2.58(303.05) | 2.53(2.26) | 1.34(1.17) |
| Underdose* | 271 | 29.52(25.67-33.95) | 29.13(5387.32) | 21.57(18.76) | 4.43(4.13) |
| Fatigue | 249 | 0.88(0.78-1) | 0.88(3.97) | 0.88(0.78) | -0.18(-0.36) |
| Diarrhoea | 227 | 0.7(0.61-0.8) | 0.7(28.5) | 0.71(0.62) | -0.5(-0.69) |
| Headache | 218 | 1.1(0.96-1.26) | 1.1(2) | 1.1(0.96) | 0.14(-0.06) |
| Device deployment issue* | 202 | 218.76(167.55-285.63) | 216.53(11620.93) | 58.79(45.02) | 5.88(5.24) |
| Rash* | 183 | 1.55(1.34-1.8) | 1.55(34.74) | 1.53(1.33) | 0.62(0.4) |
| Wrong technique in product usage process* | 179 | 2.68(2.31-3.11) | 2.67(180.84) | 2.61(2.25) | 1.38(1.15) |
| Arthralgia | 172 | 0.67(0.58-0.78) | 0.67(27.65) | 0.67(0.58) | -0.57(-0.79) |
| Device malfunction* | 160 | 7.6(6.46-8.95) | 7.55(831.14) | 6.98(5.93) | 2.8(2.51) |
| Device defective* | 150 | 90.82(71.85-114.81) | 90.14(6190.32) | 42.72(33.8) | 5.42(4.77) |
| Abdominal pain | 147 | 0.52(0.44-0.61) | 0.52(64.6) | 0.53(0.45) | -0.93(-1.16) |
| Therapeutic response decreased* | 142 | 6.72(5.66-7.98) | 6.68(632.89) | 6.24(5.25) | 2.64(2.34) |
| Pruritus* | 141 | 1.62(1.37-1.91) | 1.61(32.14) | 1.6(1.35) | 0.68(0.42) |
| Nausea | 131 | 0.6(0.51-0.71) | 0.6(34.18) | 0.61(0.51) | -0.72(-0.97) |
| Exposure during pregnancy* | 131 | 3.31(2.78-3.94) | 3.29(201.42) | 3.2(2.69) | 1.68(1.4) |
| Infusion related reaction* | 127 | 1.4(1.18-1.68) | 1.4(14.46) | 1.4(1.17) | 0.48(0.22) |
| Pain | 115 | 0.63(0.52-0.75) | 0.63(25.43) | 0.63(0.52) | -0.67(-0.93) |
| Product leakage* | 114 | 126.32(94.01-169.73) | 125.59(5455.02) | 49.23(36.64) | 5.62(4.77) |
| Therapeutic product effect decreased* | 113 | 1.81(1.5-2.18) | 1.8(39.51) | 1.78(1.48) | 0.83(0.55) |
| Drug level decreased* | 98 | 2.02(1.66-2.47) | 2.02(49.24) | 1.99(1.63) | 1(0.69) |
| Urticaria* | 96 | 2.09(1.71-2.56) | 2.09(52.95) | 2.06(1.68) | 1.04(0.73) |
| Injection site pain | 95 | 0.43(0.35-0.52) | 0.43(72.7) | 0.43(0.35) | -1.21(-1.5) |
| Incorrect dose administered | 95 | 0.74(0.6-0.91) | 0.74(8.52) | 0.74(0.61) | -0.43(-0.72) |
| Dizziness | 90 | 0.82(0.66-1.01) | 0.82(3.62) | 0.82(0.67) | -0.29(-0.59) |
| Alopecia* | 89 | 1.48(1.2-1.82) | 1.48(13.5) | 1.47(1.19) | 0.55(0.24) |
| Malaise | 86 | 0.46(0.37-0.57) | 0.47(52.9) | 0.47(0.38) | -1.09(-1.4) |
| Liquid product physical issue* | 85 | 33.85(26.26-43.65) | 33.71(1893.69) | 23.96(18.58) | 4.58(3.88) |
| Dyspnoea | 84 | 0.75(0.61-0.93) | 0.75(6.73) | 0.76(0.61) | -0.4(-0.71) |
| Drug delivery system malfunction* | 84 | 79.66(58.85-107.82) | 79.32(3248.39) | 40.16(29.67) | 5.33(4.39) |
| Covid-19 | 81 | 0.79(0.63-0.98) | 0.79(4.62) | 0.79(0.63) | -0.34(-0.66) |
| Condition aggravated | 80 | 0.31(0.25-0.39) | 0.32(119.78) | 0.32(0.26) | -1.65(-1.96) |
| Therapy non-responder* | 80 | 3.58(2.86-4.48) | 3.57(141.87) | 3.46(2.76) | 1.79(1.42) |
| Weight increased | 78 | 0.68(0.55-0.85) | 0.68(11.28) | 0.69(0.55) | -0.54(-0.86) |
| Device leakage* | 76 | 22(17.06-28.38) | 21.92(1189.2) | 17.39(13.49) | 4.12(3.47) |
| Abdominal pain upper | 65 | 0.73(0.57-0.93) | 0.73(6.65) | 0.73(0.57) | -0.46(-0.81) |
| Product quality issue* | 64 | 10.43(8.04-13.54) | 10.4(481.04) | 9.31(7.17) | 3.22(2.66) |
| Product packaging issue* | 63 | 64.27(46.1-89.6) | 64.07(2163.86) | 35.89(25.74) | 5.17(4.1) |
| Chest discomfort* | 62 | 1.72(1.34-2.22) | 1.72(18.35) | 1.71(1.33) | 0.77(0.39) |
| Nasopharyngitis | 61 | 0.53(0.42-0.69) | 0.54(24.53) | 0.54(0.42) | -0.89(-1.25) |
| Vomiting | 58 | 0.4(0.31-0.52) | 0.4(50.87) | 0.41(0.31) | -1.29(-1.66) |
| Product label issue* | 58 | 90.47(62.09-131.84) | 90.21(2394.25) | 42.74(29.33) | 5.42(4.17) |
| Erythema | 57 | 1.01(0.77-1.31) | 1.01(0) | 1.01(0.77) | 0.01(-0.37) |
| Weight decreased | 57 | 0.36(0.27-0.46) | 0.36(65.85) | 0.36(0.28) | -1.47(-1.83) |
| Back pain | 55 | 0.74(0.57-0.96) | 0.74(5.06) | 0.74(0.57) | -0.43(-0.81) |
| Myalgia | 54 | 1.21(0.93-1.59) | 1.21(1.95) | 1.21(0.92) | 0.27(-0.13) |
| Drug level below therapeutic* | 54 | 1.81(1.38-2.37) | 1.81(19.15) | 1.79(1.37) | 0.84(0.43) |
| Anxiety | 53 | 0.84(0.64-1.1) | 0.84(1.7) | 0.84(0.64) | -0.26(-0.65) |
| Hepatic enzyme increased* | 53 | 2.06(1.57-2.7) | 2.05(27.97) | 2.03(1.54) | 1.02(0.59) |
| Pyrexia | 52 | 0.33(0.25-0.43) | 0.33(72.13) | 0.33(0.25) | -1.6(-1.98) |
| Psoriasis | 50 | 1.07(0.81-1.42) | 1.07(0.24) | 1.07(0.81) | 0.1(-0.31) |
| Product complaint* | 50 | 104.63(68.61-159.58) | 104.37(2210.61) | 45.64(29.92) | 5.51(4.09) |
| Injection site reaction | 47 | 2.01(1.5-2.68) | 2(23.06) | 1.98(1.48) | 0.98(0.53) |
| Migraine* | 45 | 1.24(0.93-1.67) | 1.24(2.13) | 1.24(0.92) | 0.31(-0.13) |
| Cough | 45 | 0.48(0.36-0.65) | 0.49(24.57) | 0.49(0.36) | -1.03(-1.45) |
| Injection site haemorrhage | 44 | 0.59(0.44-0.79) | 0.59(12.76) | 0.59(0.44) | -0.76(-1.18) |
| Flushing* | 43 | 1.63(1.21-2.21) | 1.63(10.33) | 1.62(1.2) | 0.7(0.23) |
| Abdominal discomfort | 43 | 0.81(0.6-1.1) | 0.81(1.85) | 0.81(0.6) | -0.3(-0.73) |
| Muscle spasms | 42 | 0.78(0.57-1.05) | 0.78(2.7) | 0.78(0.57) | -0.36(-0.8) |
| Gastrointestinal disorder | 42 | 0.8(0.59-1.08) | 0.8(2.19) | 0.8(0.59) | -0.33(-0.76) |
| Incorrect route of product administration* | 42 | 8.04(5.85-11.05) | 8.03(234.72) | 7.38(5.37) | 2.88(2.22) |
| Asthenia | 41 | 0.38(0.28-0.51) | 0.38(41.52) | 0.38(0.28) | -1.39(-1.81) |
| Inflammation | 41 | 0.94(0.69-1.28) | 0.94(0.16) | 0.94(0.69) | -0.09(-0.54) |
| Feeling abnormal | 41 | 0.81(0.59-1.1) | 0.81(1.84) | 0.81(0.6) | -0.3(-0.74) |
| Therapy cessation* | 41 | 6.06(4.41-8.32) | 6.04(160.46) | 5.69(4.14) | 2.51(1.89) |
| Poor quality product administered* | 41 | 11.85(8.53-16.46) | 11.83(353.69) | 10.42(7.5) | 3.38(2.61) |
| Injection site erythema | 39 | 0.6(0.44-0.83) | 0.6(10.11) | 0.61(0.44) | -0.72(-1.17) |
| Frequent bowel movements | 37 | 0.32(0.23-0.44) | 0.32(54.78) | 0.32(0.23) | -1.65(-2.09) |
| Sinusitis | 36 | 0.58(0.42-0.81) | 0.58(10.63) | 0.59(0.42) | -0.77(-1.23) |
| Constipation | 36 | 0.46(0.33-0.64) | 0.46(22.77) | 0.46(0.33) | -1.11(-1.57) |
| Visual impairment* | 36 | 1.63(1.17-2.27) | 1.63(8.66) | 1.62(1.16) | 0.7(0.19) |
| Pain in extremity | 36 | 0.44(0.32-0.62) | 0.45(24.75) | 0.45(0.32) | -1.16(-1.61) |
| Hypersensitivity | 35 | 0.73(0.52-1.02) | 0.73(3.44) | 0.73(0.53) | -0.45(-0.92) |
| Adverse event* | 34 | 1.92(1.36-2.69) | 1.91(14.51) | 1.89(1.35) | 0.92(0.39) |
| Herpes zoster | 34 | 0.92(0.65-1.28) | 0.92(0.26) | 0.92(0.65) | -0.13(-0.61) |
| Abdominal distension | 34 | 0.49(0.35-0.69) | 0.49(17.86) | 0.49(0.35) | -1.02(-1.49) |
| Acne | 33 | 1.22(0.86-1.72) | 1.22(1.26) | 1.21(0.86) | 0.28(-0.23) |
| Urinary tract infection | 32 | 0.55(0.39-0.78) | 0.55(11.84) | 0.55(0.39) | -0.86(-1.34) |
| Chest pain | 31 | 0.64(0.45-0.91) | 0.64(6.17) | 0.64(0.45) | -0.63(-1.13) |
| Paraesthesia | 30 | 0.68(0.47-0.97) | 0.68(4.64) | 0.68(0.47) | -0.56(-1.07) |
| Therapeutic product effect incomplete | 30 | 0.52(0.36-0.74) | 0.52(13.34) | 0.52(0.36) | -0.94(-1.44) |
| Rash pruritic | 29 | 1.32(0.91-1.91) | 1.32(2.21) | 1.31(0.91) | 0.39(-0.15) |
| Product prescribing error* | 29 | 3.04(2.1-4.41) | 3.04(38.22) | 2.96(2.04) | 1.57(0.94) |
| Vision blurred | 28 | 1.16(0.8-1.69) | 1.16(0.63) | 1.16(0.8) | 0.21(-0.33) |
| Treatment failure | 27 | 0.86(0.59-1.25) | 0.86(0.63) | 0.86(0.59) | -0.22(-0.76) |
| Injection site bruising | 27 | 0.52(0.36-0.76) | 0.52(11.74) | 0.53(0.36) | -0.93(-1.45) |
| Product administration error* | 27 | 3.82(2.6-5.62) | 3.82(53.59) | 3.69(2.51) | 1.88(1.19) |
| Contusion | 26 | 0.87(0.59-1.29) | 0.87(0.47) | 0.88(0.59) | -0.19(-0.74) |
| Infection | 26 | 0.41(0.28-0.61) | 0.41(21.4) | 0.42(0.28) | -1.26(-1.78) |
| Depression | 25 | 0.6(0.4-0.89) | 0.6(6.65) | 0.6(0.41) | -0.73(-1.28) |
| Medication error* | 25 | 5.5(3.67-8.25) | 5.49(85.95) | 5.2(3.47) | 2.38(1.57) |
| Oropharyngeal pain | 25 | 0.4(0.27-0.59) | 0.4(22.86) | 0.4(0.27) | -1.32(-1.86) |

Abbreviation: Asterisks (*) indicate statistically significant signals in algorithm; ROR, reporting odds ratio; PRR, proportional reporting ratio; EBGM, empirical Bayesian geometric mean; EBGM05, the lower limit of the 95% CI of EBGM; IC, information component; IC025, the lower limit of the 95% CI of the IC; CI, confidence interval; PT, preferred term.

Supplementary Table 12:

Top 100 most frequent adverse events for ustekinumab at the PT level in patients with serious outcomes from FAERS data

| PT | Case numbers | ROR (95%CI) | PRR (χ^2^) | EBGM(EBGM05) | IC(IC025) |
| --- | --- | --- | --- | --- | --- |
| Product use issue* | 1409 | 10.78(10.22-11.37) | 10.45(11979.05) | 10.37(9.83) | 3.37(3.29) |
| Drug ineffective* | 1028 | 1.09(1.03-1.16) | 1.09(7.71) | 1.09(1.02) | 0.12(0.03) |
| Abdominal pain* | 656 | 4.28(3.96-4.63) | 4.23(1619.41) | 4.22(3.91) | 2.08(1.96) |
| Pneumonia* | 629 | 2.7(2.5-2.93) | 2.68(664) | 2.68(2.47) | 1.42(1.3) |
| Diarrhoea* | 624 | 1.42(1.31-1.54) | 1.41(76.06) | 1.41(1.31) | 0.5(0.38) |
| Product dose omission issue* | 596 | 1.66(1.53-1.8) | 1.65(152.68) | 1.65(1.52) | 0.72(0.6) |
| Inappropriate schedule of product administration* | 534 | 3.13(2.87-3.41) | 3.1(761.15) | 3.1(2.84) | 1.63(1.5) |
| Headache* | 517 | 1.19(1.09-1.3) | 1.19(15.96) | 1.19(1.09) | 0.25(0.12) |
| Lower respiratory tract infection* | 500 | 16.82(15.4-18.39) | 16.64(7252.11) | 16.42(15.03) | 4.04(3.86) |
| Infusion related reaction* | 465 | 11(10.04-12.06) | 10.89(4144.58) | 10.8(9.86) | 3.43(3.27) |
| Intestinal resection* | 417 | 125.35(113.27-138.72) | 124.13(46083.31) | 112.4(101.56) | 6.81(6.32) |
| Arthralgia* | 414 | 1.45(1.32-1.6) | 1.45(57.97) | 1.45(1.31) | 0.53(0.39) |
| Fatigue | 383 | 0.7(0.63-0.77) | 0.7(49.66) | 0.7(0.63) | -0.51(-0.66) |
| Nausea | 331 | 0.62(0.56-0.69) | 0.62(77.06) | 0.62(0.56) | -0.68(-0.84) |
| Infection* | 318 | 3.23(2.89-3.6) | 3.21(483.37) | 3.2(2.87) | 1.68(1.51) |
| Clostridium difficile infection* | 308 | 19.96(17.83-22.35) | 19.82(5416.13) | 19.51(17.43) | 4.29(4.04) |
| Nasopharyngitis* | 306 | 2.37(2.12-2.65) | 2.36(239.97) | 2.36(2.11) | 1.24(1.07) |
| Intestinal obstruction* | 292 | 11.88(10.58-13.33) | 11.8(2860.23) | 11.7(10.42) | 3.55(3.33) |
| Haematochezia* | 286 | 7.81(6.95-8.77) | 7.76(1675.11) | 7.72(6.87) | 2.95(2.74) |
| Abscess* | 285 | 27.36(24.32-30.79) | 27.19(7028.6) | 26.6(23.64) | 4.73(4.44) |
| Urinary tract infection* | 274 | 2.33(2.07-2.62) | 2.32(205.87) | 2.32(2.06) | 1.21(1.03) |
| Surgery* | 258 | 6.91(6.11-7.81) | 6.87(1287.97) | 6.84(6.05) | 2.77(2.56) |
| Dizziness | 230 | 0.69(0.6-0.78) | 0.69(33.1) | 0.69(0.6) | -0.54(-0.73) |
| General physical health deterioration* | 229 | 3.04(2.67-3.46) | 3.03(310.88) | 3.02(2.65) | 1.6(1.39) |
| Exposure during pregnancy* | 228 | 4.22(3.7-4.81) | 4.2(555.23) | 4.19(3.68) | 2.07(1.86) |
| Dyspnoea | 227 | 0.58(0.51-0.67) | 0.59(66.92) | 0.59(0.51) | -0.77(-0.96) |
| Hospitalisation* | 221 | 2.05(1.79-2.34) | 2.04(117.33) | 2.04(1.79) | 1.03(0.83) |
| Influenza* | 215 | 2.86(2.5-3.27) | 2.85(258.81) | 2.85(2.49) | 1.51(1.3) |
| Therapeutic response decreased* | 213 | 5.18(4.52-5.92) | 5.15(710.89) | 5.14(4.49) | 2.36(2.14) |
| Vomiting | 209 | 0.67(0.59-0.77) | 0.67(33.42) | 0.67(0.59) | -0.57(-0.77) |
| Anal abscess* | 207 | 57.83(50.28-66.51) | 57.55(10967.87) | 54.92(47.75) | 5.78(5.24) |
| Pain | 206 | 0.46(0.4-0.53) | 0.46(131.3) | 0.46(0.4) | -1.12(-1.31) |
| Fistula* | 197 | 26.54(23.04-30.58) | 26.42(4713.76) | 25.86(22.45) | 4.69(4.31) |
| Sinusitis* | 191 | 2.66(2.31-3.07) | 2.65(196.77) | 2.65(2.3) | 1.41(1.18) |
| Therapeutic product effect decreased* | 190 | 2.72(2.36-3.13) | 2.71(204.72) | 2.71(2.35) | 1.44(1.21) |
| Pyrexia | 186 | 0.8(0.69-0.92) | 0.8(9.24) | 0.8(0.69) | -0.32(-0.53) |
| Frequent bowel movements* | 179 | 10.33(8.92-11.98) | 10.3(1489.86) | 10.21(8.81) | 3.35(3.06) |
| Covid-19* | 178 | 1.34(1.16-1.55) | 1.34(15.42) | 1.34(1.16) | 0.42(0.2) |
| Malaise | 176 | 0.55(0.48-0.64) | 0.55(63.31) | 0.56(0.48) | -0.85(-1.06) |
| Colectomy* | 176 | 46.18(39.71-53.7) | 45.99(7456.12) | 44.3(38.1) | 5.47(4.93) |
| Rash | 172 | 0.55(0.48-0.64) | 0.55(62.24) | 0.55(0.48) | -0.85(-1.07) |
| Sepsis* | 155 | 2.06(1.76-2.41) | 2.05(83.55) | 2.05(1.75) | 1.04(0.79) |
| Weight decreased | 152 | 0.82(0.7-0.96) | 0.82(6.29) | 0.82(0.7) | -0.29(-0.52) |
| Cellulitis* | 147 | 4.21(3.58-4.95) | 4.2(357.12) | 4.19(3.56) | 2.07(1.8) |
| Pruritus | 140 | 0.53(0.45-0.63) | 0.53(57.27) | 0.53(0.45) | -0.9(-1.14) |
| Fall | 132 | 0.57(0.48-0.68) | 0.58(41.63) | 0.58(0.48) | -0.8(-1.04) |
| Nephrolithiasis* | 127 | 4.1(3.45-4.88) | 4.09(296.07) | 4.08(3.43) | 2.03(1.74) |
| Kidney infection* | 126 | 9.24(7.75-11.02) | 9.22(916.18) | 9.15(7.68) | 3.19(2.85) |
| Back pain | 120 | 0.74(0.62-0.89) | 0.74(10.86) | 0.74(0.62) | -0.43(-0.69) |
| Product use in unapproved indication | 120 | 0.71(0.59-0.85) | 0.71(14.13) | 0.71(0.59) | -0.49(-0.75) |
| Death | 116 | 0.18(0.15-0.22) | 0.19(418.99) | 0.19(0.16) | -2.42(-2.68) |
| Migraine* | 115 | 1.77(1.47-2.13) | 1.77(38.32) | 1.77(1.47) | 0.82(0.54) |
| Loss of consciousness* | 115 | 1.41(1.17-1.69) | 1.41(13.7) | 1.41(1.17) | 0.49(0.22) |
| Asthenia | 114 | 0.45(0.37-0.54) | 0.45(78.53) | 0.45(0.37) | -1.16(-1.42) |
| Constipation | 113 | 0.78(0.65-0.94) | 0.78(6.88) | 0.78(0.65) | -0.35(-0.62) |
| Hypersensitivity | 112 | 0.87(0.72-1.05) | 0.87(2.18) | 0.87(0.72) | -0.2(-0.47) |
| Condition aggravated | 111 | 0.57(0.47-0.68) | 0.57(36.95) | 0.57(0.47) | -0.82(-1.09) |
| Cerebrovascular accident | 111 | 1.03(0.85-1.24) | 1.03(0.07) | 1.03(0.85) | 0.04(-0.24) |
| Hypertension | 106 | 0.75(0.62-0.9) | 0.75(9.01) | 0.75(0.62) | -0.42(-0.69) |
| Abdominal pain upper | 105 | 0.75(0.62-0.91) | 0.75(8.81) | 0.75(0.62) | -0.42(-0.69) |
| Intestinal stenosis* | 100 | 34.2(28.02-41.73) | 34.12(3124.56) | 33.19(27.2) | 5.05(4.36) |
| Herpes zoster* | 99 | 2.41(1.98-2.93) | 2.41(81.22) | 2.4(1.97) | 1.26(0.96) |
| Seizure | 96 | 0.87(0.71-1.06) | 0.87(1.96) | 0.87(0.71) | -0.21(-0.5) |
| Bronchitis* | 96 | 1.82(1.49-2.23) | 1.82(35.47) | 1.82(1.49) | 0.86(0.56) |
| Cough | 94 | 0.49(0.4-0.59) | 0.49(51.21) | 0.49(0.4) | -1.04(-1.33) |
| Abdominal abscess* | 93 | 31.99(26.03-39.32) | 31.92(2712.59) | 31.11(25.31) | 4.96(4.26) |
| Dehydration | 92 | 1.05(0.85-1.29) | 1.05(0.21) | 1.05(0.85) | 0.07(-0.23) |
| Arthritis* | 89 | 1.6(1.3-1.97) | 1.6(19.9) | 1.6(1.3) | 0.68(0.36) |
| Drug level decreased* | 89 | 13.5(10.95-16.64) | 13.47(1016.38) | 13.33(10.82) | 3.74(3.25) |
| Pain in extremity | 89 | 0.42(0.35-0.52) | 0.43(69.11) | 0.43(0.35) | -1.23(-1.53) |
| Anaemia | 88 | 0.67(0.54-0.83) | 0.67(14.27) | 0.67(0.54) | -0.58(-0.88) |
| Anaphylactic reaction* | 88 | 2.5(2.03-3.09) | 2.5(79.12) | 2.5(2.03) | 1.32(0.99) |
| Rectal haemorrhage* | 88 | 3.04(2.47-3.75) | 3.04(119.99) | 3.03(2.46) | 1.6(1.26) |
| Anxiety | 87 | 0.44(0.35-0.54) | 0.44(63.3) | 0.44(0.35) | -1.19(-1.49) |
| Haemorrhage | 84 | 1.19(0.96-1.47) | 1.19(2.45) | 1.19(0.96) | 0.25(-0.07) |
| Tooth abscess* | 84 | 11.87(9.57-14.72) | 11.85(826.44) | 11.74(9.47) | 3.55(3.07) |
| Syncope | 83 | 1.24(1-1.54) | 1.24(3.85) | 1.24(1) | 0.31(-0.01) |
| Chest pain | 83 | 0.69(0.56-0.85) | 0.69(11.63) | 0.69(0.56) | -0.54(-0.85) |
| Alopecia | 82 | 0.57(0.46-0.71) | 0.57(26.01) | 0.57(0.46) | -0.8(-1.11) |
| Skin cancer* | 82 | 5.3(4.27-6.59) | 5.3(284.57) | 5.28(4.25) | 2.4(2.01) |
| Insomnia | 81 | 0.44(0.36-0.55) | 0.44(56.57) | 0.44(0.36) | -1.17(-1.48) |
| Psoriasis | 81 | 0.85(0.68-1.05) | 0.85(2.23) | 0.85(0.68) | -0.24(-0.55) |
| Syringe issue* | 81 | 6.39(5.14-7.95) | 6.38(365.67) | 6.35(5.1) | 2.67(2.26) |
| Gastrointestinal infection* | 79 | 13.2(10.57-16.48) | 13.18(879.45) | 13.04(10.45) | 3.71(3.18) |
| Postoperative wound infection* | 77 | 14.58(11.64-18.26) | 14.56(960.49) | 14.39(11.49) | 3.85(3.29) |
| Rectal abscess* | 76 | 67.28(53.38-84.79) | 67.16(4686.25) | 63.59(50.46) | 5.99(4.79) |
| Respiratory tract infection* | 76 | 4.37(3.48-5.47) | 4.36(196.1) | 4.35(3.47) | 2.12(1.73) |
| Anal fistula* | 75 | 20.2(16.07-25.38) | 20.17(1343.33) | 19.84(15.79) | 4.31(3.66) |
| Post procedural infection* | 75 | 13.35(10.63-16.76) | 13.33(845.68) | 13.19(10.5) | 3.72(3.17) |
| Oropharyngeal pain | 73 | 1.08(0.86-1.36) | 1.08(0.46) | 1.08(0.86) | 0.11(-0.22) |
| Abscess intestinal* | 73 | 51.43(40.68-65.02) | 51.34(3453.21) | 49.24(38.95) | 5.62(4.55) |
| Paraesthesia | 72 | 0.66(0.53-0.84) | 0.67(12.15) | 0.67(0.53) | -0.59(-0.92) |
| Incorrect dose administered | 72 | 0.5(0.39-0.63) | 0.5(36.59) | 0.5(0.4) | -1.01(-1.33) |
| Abdominal distension | 71 | 1.03(0.81-1.3) | 1.03(0.05) | 1.03(0.81) | 0.04(-0.3) |
| Neoplasm malignant* | 71 | 1.45(1.15-1.83) | 1.45(10.01) | 1.45(1.15) | 0.54(0.19) |
| Erythema | 70 | 0.47(0.37-0.6) | 0.47(40.83) | 0.47(0.38) | -1.07(-1.41) |
| Ear infection* | 70 | 3.86(3.05-4.88) | 3.85(147.31) | 3.84(3.04) | 1.94(1.54) |
| Chest discomfort | 70 | 1.02(0.81-1.29) | 1.02(0.04) | 1.02(0.81) | 0.03(-0.31) |
| Ileostomy* | 70 | 44.55(35.09-56.57) | 44.48(2867.27) | 42.9(33.79) | 5.42(4.4) |
| Weight increased | 69 | 0.47(0.37-0.59) | 0.47(41.75) | 0.47(0.37) | -1.09(-1.43) |

Abbreviation: Asterisks (*) indicate statistically significant signals in algorithm; ROR, reporting odds ratio; PRR, proportional reporting ratio; EBGM, empirical Bayesian geometric mean; EBGM05, the lower limit of the 95% CI of EBGM; IC, information component; IC025, the lower limit of the 95% CI of the IC; CI, confidence interval; PT, preferred term.

Supplementary Table 13:

Top 100 most frequent adverse events for ustekinumab at the PT level in pregnant patients from FAERS data

| PT | Case numbers | ROR (95%CI) | PRR (χ^2^) | EBGM(EBGM05) | IC(IC025) |
| --- | --- | --- | --- | --- | --- |
| Abortion spontaneous* | 69 | 2.07(1.62-2.64) | 2.02(34.94) | 1.98(1.55) | 0.99(0.61) |
| Product use issue* | 28 | 2.14(1.46-3.14) | 2.12(16.01) | 2.07(1.41) | 1.05(0.44) |
| Drug ineffective | 26 | 1.46(0.99-2.17) | 1.46(3.64) | 1.44(0.97) | 0.53(-0.07) |
| Premature baby* | 25 | 2.84(1.89-4.26) | 2.81(27.63) | 2.71(1.8) | 1.44(0.75) |
| Caesarean section* | 22 | 2.37(1.54-3.64) | 2.35(16.28) | 2.28(1.48) | 1.19(0.49) |
| Product dose omission issue* | 21 | 8.7(5.45-13.9) | 8.61(119.23) | 7.41(4.64) | 2.89(1.85) |
| Premature labor* | 14 | 2.26(1.32-3.88) | 2.25(9.32) | 2.19(1.28) | 1.13(0.25) |
| Maternal exposure during pregnancy | 13 | 0.21(0.12-0.37) | 0.22(37.3) | 0.22(0.13) | -2.17(-2.86) |
| Inappropriate schedule of product administration | 12 | 1.16(0.65-2.05) | 1.16(0.25) | 1.15(0.65) | 0.2(-0.63) |
| Arthralgia | 10 | 0.66(0.35-1.23) | 0.66(1.72) | 0.67(0.36) | -0.59(-1.42) |
| Pneumonia | 10 | 1.51(0.8-2.84) | 1.51(1.67) | 1.49(0.79) | 0.58(-0.38) |
| Headache | 9 | 0.63(0.33-1.23) | 0.64(1.86) | 0.64(0.33) | -0.64(-1.51) |
| Sinusitis | 9 | 1.59(0.82-3.1) | 1.59(1.91) | 1.57(0.81) | 0.65(-0.37) |
| Urinary tract infection* | 8 | 2.4(1.18-4.89) | 2.39(6.16) | 2.32(1.14) | 1.22(0.02) |
| Premature delivery | 8 | 1.54(0.76-3.12) | 1.54(1.46) | 1.52(0.75) | 0.6(-0.46) |
| Exposure via breast milk* | 8 | 2.97(1.45-6.09) | 2.96(9.8) | 2.85(1.39) | 1.51(0.24) |
| Infusion related reaction | 8 | 0.7(0.35-1.4) | 0.7(1.04) | 0.7(0.35) | -0.51(-1.44) |
| Fatigue | 7 | 0.44(0.21-0.93) | 0.44(4.92) | 0.45(0.21) | -1.16(-2.09) |
| Abdominal pain | 7 | 0.65(0.31-1.38) | 0.65(1.26) | 0.66(0.31) | -0.6(-1.57) |
| Pregnancy | 7 | 0.23(0.11-0.49) | 0.24(17.56) | 0.24(0.11) | -2.06(-2.94) |
| Nasopharyngitis | 6 | 0.66(0.29-1.47) | 0.66(1.07) | 0.66(0.3) | -0.6(-1.63) |
| Therapeutic product effect decreased | 6 | 0.75(0.33-1.68) | 0.75(0.5) | 0.75(0.34) | -0.41(-1.46) |
| Product use in unapproved indication | 6 | 1.02(0.45-2.3) | 1.02(0) | 1.02(0.45) | 0.03(-1.09) |
| Clostridium difficile infection* | 6 | 4.42(1.91-10.23) | 4.41(14.46) | 4.11(1.78) | 2.04(0.35) |
| Therapeutic response decreased* | 6 | 4.89(2.1-11.35) | 4.87(16.73) | 4.51(1.94) | 2.17(0.42) |
| Nausea | 5 | 0.37(0.16-0.9) | 0.38(5.16) | 0.38(0.16) | -1.39(-2.42) |
| Abscess* | 5 | 4.74(1.88-11.9) | 4.72(13.33) | 4.38(1.74) | 2.13(0.23) |
| Pain | 5 | 0.36(0.15-0.88) | 0.37(5.52) | 0.37(0.15) | -1.43(-2.46) |
| Pruritus | 5 | 0.76(0.31-1.83) | 0.76(0.39) | 0.76(0.31) | -0.39(-1.53) |
| Vomiting | 5 | 0.63(0.26-1.54) | 0.64(1.03) | 0.64(0.26) | -0.64(-1.75) |
| Needle issue* | 5 | 77.4(18.48-324.15) | 77.17(140.99) | 29.57(7.06) | 4.89(0.81) |
| Hospitalisation* | 5 | 3.14(1.27-7.76) | 3.13(6.79) | 2.99(1.21) | 1.58(-0.07) |
| Diarrhoea | 5 | 0.36(0.15-0.87) | 0.36(5.67) | 0.37(0.15) | -1.45(-2.48) |
| Chest discomfort* | 5 | 7.25(2.82-18.64) | 7.24(23.25) | 6.39(2.49) | 2.68(0.46) |
| Foetal death | 5 | 0.71(0.29-1.72) | 0.71(0.57) | 0.72(0.3) | -0.48(-1.61) |
| Gastroenteritis* | 5 | 11.05(4.16-29.35) | 11.02(36.82) | 9.1(3.43) | 3.19(0.63) |
| Syringe issue* | 5 | 232.21(27.11-1988.73) | 231.52(191.29) | 39.42(4.6) | 5.3(0.78) |
| Infection | 5 | 0.6(0.25-1.45) | 0.6(1.32) | 0.6(0.25) | -0.73(-1.82) |
| Gestational diabetes | 5 | 1.26(0.52-3.06) | 1.26(0.26) | 1.25(0.51) | 0.32(-0.94) |
| Ectopic pregnancy | 5 | 1.93(0.79-4.73) | 1.93(2.15) | 1.89(0.77) | 0.92(-0.5) |
| Dyspnoea | 4 | 0.61(0.23-1.65) | 0.62(0.95) | 0.62(0.23) | -0.69(-1.88) |
| Anaemia | 4 | 1.43(0.53-3.86) | 1.42(0.49) | 1.41(0.52) | 0.5(-0.94) |
| Rash | 4 | 0.35(0.13-0.94) | 0.35(4.74) | 0.36(0.13) | -1.48(-2.59) |
| Asthenia | 4 | 0.94(0.35-2.53) | 0.94(0.01) | 0.94(0.35) | -0.09(-1.38) |
| Abortion | 4 | 1.99(0.73-5.43) | 1.99(1.9) | 1.95(0.72) | 0.96(-0.62) |
| Abortion induced | 4 | 1.08(0.4-2.91) | 1.08(0.02) | 1.08(0.4) | 0.1(-1.23) |
| Pyelonephritis* | 4 | 37.13(9.96-138.39) | 37.04(77.94) | 21.02(5.64) | 4.39(0.46) |
| Pre-eclampsia | 4 | 0.91(0.34-2.46) | 0.91(0.03) | 0.91(0.34) | -0.13(-1.42) |
| Hyperemesis gravidarum* | 4 | 14.28(4.65-43.84) | 14.25(37.69) | 11.13(3.63) | 3.48(0.4) |
| Low birth weight baby | 4 | 2.38(0.87-6.5) | 2.37(3.03) | 2.31(0.84) | 1.21(-0.47) |
| Atrial septal defect* | 4 | 4.64(1.66-12.98) | 4.63(10.36) | 4.3(1.54) | 2.1(0) |
| Pregnancy of partner | 4 | 2.69(0.98-7.37) | 2.68(4) | 2.59(0.94) | 1.37(-0.37) |
| Frequent bowel movements | 4 | 0.69(0.26-1.85) | 0.69(0.56) | 0.69(0.26) | -0.53(-1.75) |
| Escherichia infection* | 4 | 26.52(7.76-90.68) | 26.46(62.37) | 17.2(5.03) | 4.1(0.46) |
| Congenital anomaly* | 4 | 8.44(2.9-24.51) | 8.42(22.13) | 7.28(2.51) | 2.86(0.26) |
| Upper respiratory tract infection* | 4 | 2.9(1.05-7.97) | 2.89(4.67) | 2.78(1.01) | 1.48(-0.31) |
| Lower respiratory tract infection | 4 | 0.86(0.32-2.31) | 0.86(0.09) | 0.86(0.32) | -0.22(-1.49) |
| General physical health deterioration | 4 | 0.43(0.16-1.14) | 0.43(3.07) | 0.43(0.16) | -1.21(-2.34) |
| Mastitis | 3 | 2.73(0.85-8.75) | 2.72(3.09) | 2.63(0.82) | 1.39(-0.61) |
| Alopecia | 3 | 0.3(0.1-0.93) | 0.3(4.88) | 0.31(0.1) | -1.71(-2.89) |
| Malaise | 3 | 0.22(0.07-0.69) | 0.22(8.23) | 0.23(0.07) | -2.15(-3.29) |
| Anxiety | 3 | 0.64(0.21-2.01) | 0.64(0.59) | 0.65(0.21) | -0.63(-1.96) |
| Hypersensitivity | 3 | 0.31(0.1-0.98) | 0.32(4.44) | 0.32(0.1) | -1.64(-2.83) |
| Anal fistula | 3 | 1.99(0.62-6.31) | 1.98(1.41) | 1.94(0.61) | 0.96(-0.84) |
| Bronchitis | 3 | 1.74(0.55-5.51) | 1.74(0.9) | 1.71(0.54) | 0.77(-0.95) |
| Wound infection | 3 | 1.29(0.41-4.06) | 1.29(0.19) | 1.28(0.41) | 0.35(-1.22) |
| Ear infection | 3 | 0.6(0.19-1.88) | 0.6(0.78) | 0.61(0.19) | -0.72(-2.03) |
| Haemangioma* | 3 | 69.58(11.62-416.69) | 69.46(80.97) | 28.38(4.74) | 4.83(-0.04) |
| Anal abscess | 3 | 2.67(0.83-8.57) | 2.67(2.97) | 2.58(0.8) | 1.37(-0.62) |
| Dizziness | 3 | 0.41(0.13-1.27) | 0.41(2.54) | 0.41(0.13) | -1.27(-2.5) |
| Bacterial vaginosis* | 3 | 13.91(3.83-50.61) | 13.89(27.61) | 10.92(3) | 3.45(-0.01) |
| Therapy cessation* | 3 | 7.32(2.16-24.77) | 7.31(14.12) | 6.45(1.91) | 2.69(-0.14) |
| Peripheral swelling | 3 | 0.64(0.2-2) | 0.64(0.6) | 0.65(0.21) | -0.63(-1.96) |
| Breech presentation | 3 | 5.15(1.56-17) | 5.14(9.02) | 4.73(1.43) | 2.24(-0.26) |
| Fallot's tetralogy | 3 | 46.39(9.36-229.99) | 46.3(66.49) | 23.65(4.77) | 4.56(-0.01) |
| Condition aggravated | 3 | 0.17(0.05-0.52) | 0.17(12.32) | 0.17(0.06) | -2.54(-3.65) |
| Abdominal abscess* | 3 | 8.18(2.4-27.95) | 8.17(16.05) | 7.1(2.08) | 2.83(-0.11) |
| Foetal growth restriction | 3 | 1.64(0.52-5.18) | 1.63(0.71) | 1.61(0.51) | 0.69(-1) |
| Premature rupture of membranes | 3 | 2.73(0.85-8.75) | 2.72(3.09) | 2.63(0.82) | 1.39(-0.61) |
| Ventricular septal defect* | 3 | 6.05(1.81-20.17) | 6.04(11.16) | 5.46(1.64) | 2.45(-0.2) |
| Gastrointestinal disorder | 3 | 0.6(0.19-1.86) | 0.6(0.81) | 0.6(0.19) | -0.73(-2.04) |
| Beta haemolytic streptococcal infection* | 3 | 7.73(2.27-26.27) | 7.72(15.04) | 6.76(1.99) | 2.76(-0.12) |
| Incorrect dose administered | 3 | 1.08(0.34-3.39) | 1.08(0.02) | 1.08(0.34) | 0.1(-1.4) |
| Premature separation of placenta | 3 | 2.78(0.87-8.93) | 2.78(3.22) | 2.68(0.83) | 1.42(-0.6) |
| Reversible cerebral vasoconstriction syndrome* | 3 | 69.58(11.62-416.69) | 69.46(80.97) | 28.38(4.74) | 4.83(-0.04) |
| Respiratory tract infection* | 3 | 3.66(1.13-11.87) | 3.66(5.37) | 3.46(1.07) | 1.79(-0.43) |
| Sepsis | 2 | 2.9(0.69-12.1) | 2.89(2.33) | 2.78(0.67) | 1.48(-0.95) |
| Pyrexia | 2 | 0.22(0.05-0.88) | 0.22(5.58) | 0.22(0.06) | -2.17(-3.41) |
| Talipes | 2 | 8.43(1.87-38.05) | 8.42(11.07) | 7.28(1.61) | 2.86(-0.63) |
| Jaundice | 2 | 7.73(1.73-34.55) | 7.72(10.03) | 6.76(1.51) | 2.76(-0.64) |
| Cyanosis | 2 | 46.36(6.53-329.31) | 46.3(44.33) | 23.65(3.33) | 4.56(-0.67) |
| Covid-19 | 2 | 0.43(0.11-1.72) | 0.43(1.51) | 0.43(0.11) | -1.2(-2.59) |
| Swelling | 2 | 0.27(0.07-1.08) | 0.27(3.92) | 0.28(0.07) | -1.86(-3.14) |
| Fistula | 2 | 1.22(0.3-4.97) | 1.22(0.08) | 1.21(0.3) | 0.28(-1.53) |
| Acne | 2 | 2.21(0.53-9.12) | 2.2(1.26) | 2.15(0.52) | 1.1(-1.1) |
| Myalgia | 2 | 1.85(0.45-7.62) | 1.85(0.75) | 1.82(0.44) | 0.86(-1.21) |
| Fall | 2 | 2.44(0.59-10.12) | 2.44(1.61) | 2.37(0.57) | 1.24(-1.04) |
| Cough | 2 | 0.64(0.16-2.6) | 0.64(0.39) | 0.65(0.16) | -0.63(-2.14) |
| Urticaria | 2 | 0.45(0.11-1.8) | 0.45(1.36) | 0.45(0.11) | -1.14(-2.54) |
| Constipation | 2 | 0.52(0.13-2.08) | 0.52(0.89) | 0.52(0.13) | -0.94(-2.37) |

Abbreviation: Asterisks (*) indicate statistically significant signals in algorithm; ROR, reporting odds ratio; PRR, proportional reporting ratio; EBGM, empirical Bayesian geometric mean; EBGM05, the lower limit of the 95% CI of EBGM; IC, information component; IC025, the lower limit of the 95% CI of the IC; CI, confidence interval; PT, preferred term.

Supplementary Table 14:

Top 100 most frequent adverse events for ustekinumab excluding common medication co-usage at the PT level from FAERS data

| PT | Case numbers | ROR (95%CI) | PRR (χ^2^) | EBGM(EBGM05) | IC(IC025) |
| --- | --- | --- | --- | --- | --- |
| Product dose omission issue* | 2505 | 18.2(17.3-19.15) | 17.4(23853.01) | 11.06(10.6) | 3.47(3.4) |
| Drug ineffective* | 1808 | 1.46(1.39-1.53) | 1.44(237.24) | 1.42(1.36) | 0.5(0.43) |
| Product use issue* | 1559 | 5.12(4.85-5.41) | 5(4254.71) | 4.39(4.19) | 2.13(2.05) |
| Inappropriate schedule of product administration* | 1043 | 2.55(2.39-2.72) | 2.52(881.69) | 2.39(2.27) | 1.26(1.16) |
| Diarrhoea | 735 | 0.82(0.76-0.88) | 0.82(29.37) | 0.82(0.77) | -0.28(-0.39) |
| Abdominal pain | 708 | 0.9(0.84-0.97) | 0.9(7.04) | 0.91(0.85) | -0.14(-0.25) |
| Headache* | 641 | 1.17(1.08-1.26) | 1.16(14.38) | 1.16(1.08) | 0.21(0.09) |
| Syringe issue* | 633 | 129.08(107.29-155.3) | 127.57(14150.71) | 23.52(20.15) | 4.56(4.4) |
| Needle issue* | 613 | 35.81(31.76-40.37) | 35.41(8986.7) | 16.08(14.54) | 4.01(3.86) |
| Fatigue | 553 | 0.69(0.64-0.76) | 0.7(71.87) | 0.7(0.66) | -0.5(-0.63) |
| Pneumonia* | 546 | 2.27(2.08-2.48) | 2.25(354.01) | 2.16(2.01) | 1.11(0.98) |
| Infusion related reaction* | 514 | 2.14(1.96-2.35) | 2.13(288.68) | 2.05(1.9) | 1.04(0.91) |
| Arthralgia | 511 | 0.71(0.65-0.78) | 0.71(58.52) | 0.72(0.67) | -0.47(-0.6) |
| Lower respiratory tract infection* | 469 | 7.91(7.13-8.77) | 7.85(2185.27) | 6.33(5.81) | 2.66(2.52) |
| Accidental exposure to product* | 463 | 28.08(24.68-31.96) | 27.85(5970.5) | 14.37(12.89) | 3.84(3.68) |
| Device issue* | 451 | 2.03(1.85-2.24) | 2.03(218.98) | 1.96(1.8) | 0.97(0.83) |
| Intestinal resection* | 403 | 3.86(3.48-4.29) | 3.84(744.19) | 3.49(3.2) | 1.8(1.65) |
| Nausea | 398 | 0.65(0.59-0.72) | 0.65(72.08) | 0.66(0.61) | -0.6(-0.74) |
| Product storage error* | 381 | 20.02(17.55-22.85) | 19.89(3974.94) | 11.98(10.73) | 3.58(3.41) |
| Nasopharyngitis | 335 | 1.07(0.96-1.19) | 1.07(1.5) | 1.07(0.97) | 0.09(-0.07) |
| Therapeutic response decreased* | 327 | 6.3(5.58-7.1) | 6.27(1180.76) | 5.29(4.78) | 2.4(2.23) |
| Rash | 313 | 0.99(0.89-1.11) | 0.99(0.01) | 0.99(0.9) | -0.01(-0.17) |
| Exposure during pregnancy* | 309 | 3.17(2.82-3.57) | 3.16(410.71) | 2.94(2.66) | 1.56(1.38) |
| Infection* | 304 | 1.85(1.65-2.08) | 1.85(111.23) | 1.79(1.63) | 0.84(0.67) |
| Dizziness | 287 | 0.94(0.83-1.05) | 0.94(1.19) | 0.94(0.85) | -0.09(-0.26) |
| Underdose* | 286 | 11.1(9.67-12.73) | 11.04(1867.34) | 8.17(7.29) | 3.03(2.84) |
| Urinary tract infection* | 280 | 1.78(1.58-2.01) | 1.77(89.19) | 1.73(1.56) | 0.79(0.61) |
| Pain | 280 | 0.54(0.48-0.61) | 0.54(105.54) | 0.55(0.5) | -0.85(-1.03) |
| Clostridium difficile infection* | 274 | 1.96(1.73-2.21) | 1.95(119.02) | 1.89(1.7) | 0.92(0.74) |
| Abscess* | 269 | 2.35(2.08-2.67) | 2.35(192.18) | 2.24(2.02) | 1.16(0.98) |
| Intestinal obstruction | 267 | 0.88(0.78-1) | 0.88(4.07) | 0.89(0.8) | -0.17(-0.35) |
| Dyspnoea | 266 | 0.86(0.76-0.97) | 0.86(5.7) | 0.87(0.78) | -0.21(-0.39) |
| Haematochezia | 261 | 0.57(0.5-0.64) | 0.57(82.86) | 0.58(0.52) | -0.79(-0.97) |
| Surgery* | 251 | 2.5(2.2-2.85) | 2.5(206.9) | 2.37(2.13) | 1.25(1.06) |
| Pruritus | 241 | 1.05(0.92-1.19) | 1.05(0.49) | 1.05(0.94) | 0.06(-0.13) |
| Vomiting | 237 | 0.59(0.52-0.67) | 0.59(65.34) | 0.6(0.54) | -0.73(-0.92) |
| Malaise | 232 | 0.44(0.39-0.51) | 0.45(158.25) | 0.46(0.41) | -1.14(-1.33) |
| Covid-19 | 231 | 0.79(0.69-0.9) | 0.79(12.37) | 0.8(0.71) | -0.33(-0.52) |
| Therapeutic product effect decreased* | 222 | 2.18(1.9-2.5) | 2.18(131.28) | 2.09(1.87) | 1.06(0.86) |
| Device deployment issue* | 217 | 120.31(88.46-163.64) | 119.83(4789.43) | 23.25(17.98) | 4.54(4.28) |
| General physical health deterioration* | 214 | 1.73(1.51-1.99) | 1.73(61.61) | 1.68(1.5) | 0.75(0.55) |
| Influenza* | 204 | 1.37(1.19-1.58) | 1.37(19.73) | 1.36(1.2) | 0.44(0.23) |
| Hospitalisation* | 203 | 1.59(1.38-1.84) | 1.59(42.18) | 1.56(1.38) | 0.64(0.43) |
| Pyrexia | 202 | 0.45(0.39-0.52) | 0.45(132.46) | 0.46(0.41) | -1.12(-1.32) |
| Sinusitis* | 195 | 1.16(1.01-1.34) | 1.16(4.28) | 1.16(1.03) | 0.21(0) |
| Fistula | 192 | 1.06(0.92-1.23) | 1.06(0.72) | 1.06(0.94) | 0.09(-0.13) |
| Frequent bowel movements | 190 | 0.58(0.5-0.67) | 0.58(56.63) | 0.59(0.52) | -0.76(-0.97) |
| Wrong technique in product usage process* | 189 | 1.36(1.17-1.57) | 1.35(16.72) | 1.34(1.18) | 0.42(0.21) |
| Weight decreased | 186 | 0.41(0.36-0.48) | 0.42(151.58) | 0.42(0.38) | -1.24(-1.45) |
| Drug level decreased* | 171 | 1.26(1.08-1.46) | 1.26(8.5) | 1.24(1.09) | 0.31(0.09) |
| Anal abscess* | 167 | 2.38(2.03-2.79) | 2.38(122.8) | 2.27(1.99) | 1.18(0.95) |
| Device defective* | 167 | 53.78(41.46-69.77) | 53.62(2931.64) | 18.89(15.19) | 4.24(3.95) |
| Colectomy* | 165 | 1.76(1.51-2.06) | 1.76(51.1) | 1.72(1.5) | 0.78(0.55) |
| Device malfunction* | 163 | 2.75(2.34-3.23) | 2.75(164.98) | 2.59(2.26) | 1.37(1.14) |
| Incorrect dose administered | 153 | 0.42(0.36-0.49) | 0.42(122.73) | 0.43(0.37) | -1.23(-1.46) |
| Condition aggravated | 151 | 0.2(0.17-0.24) | 0.2(469.1) | 0.21(0.18) | -2.25(-2.48) |
| Back pain | 149 | 0.71(0.61-0.84) | 0.71(16.65) | 0.72(0.63) | -0.47(-0.71) |
| Abdominal pain upper | 148 | 0.59(0.5-0.7) | 0.59(40.91) | 0.6(0.52) | -0.74(-0.97) |
| Urticaria | 147 | 1.16(0.98-1.37) | 1.16(3.07) | 1.15(1) | 0.2(-0.04) |
| Alopecia | 147 | 0.88(0.75-1.04) | 0.88(2.36) | 0.88(0.77) | -0.18(-0.42) |
| Migraine* | 146 | 1.5(1.27-1.77) | 1.5(23.14) | 1.47(1.28) | 0.56(0.32) |
| Injection site pain | 141 | 0.22(0.19-0.26) | 0.22(384) | 0.23(0.2) | -2.13(-2.37) |
| Cellulitis* | 139 | 2.44(2.05-2.91) | 2.44(108.48) | 2.32(2.01) | 1.22(0.96) |
| Asthenia | 138 | 0.46(0.39-0.54) | 0.46(87.97) | 0.47(0.4) | -1.1(-1.35) |
| Constipation | 136 | 0.62(0.52-0.73) | 0.62(31.45) | 0.63(0.54) | -0.67(-0.92) |
| Sepsis* | 132 | 1.55(1.3-1.84) | 1.54(23.98) | 1.51(1.31) | 0.6(0.34) |
| Kidney infection* | 130 | 4.61(3.83-5.55) | 4.6(314.32) | 4.09(3.5) | 2.03(1.76) |
| Fall | 128 | 0.74(0.62-0.88) | 0.74(11.15) | 0.75(0.65) | -0.42(-0.67) |
| Weight increased | 126 | 0.39(0.33-0.46) | 0.39(118.83) | 0.4(0.34) | -1.33(-1.58) |
| Cough | 126 | 0.48(0.4-0.57) | 0.48(70.75) | 0.49(0.42) | -1.04(-1.3) |
| Anxiety | 124 | 0.7(0.59-0.84) | 0.7(15.29) | 0.71(0.61) | -0.49(-0.76) |
| Herpes zoster | 121 | 1.19(0.99-1.42) | 1.18(3.35) | 1.18(1.01) | 0.24(-0.03) |
| Product leakage* | 121 | 60.88(44.26-83.74) | 60.74(2222.13) | 19.67(15.06) | 4.3(3.96) |
| Hypersensitivity | 119 | 0.91(0.76-1.09) | 0.91(1.01) | 0.91(0.78) | -0.13(-0.4) |
| Psoriasis | 118 | 0.91(0.76-1.1) | 0.91(0.91) | 0.92(0.79) | -0.12(-0.39) |
| Nephrolithiasis | 117 | 1.09(0.91-1.32) | 1.09(0.91) | 1.09(0.93) | 0.12(-0.15) |
| Chest discomfort | 113 | 1.12(0.93-1.35) | 1.12(1.34) | 1.11(0.95) | 0.15(-0.12) |
| Erythema | 113 | 0.71(0.59-0.86) | 0.71(12.92) | 0.72(0.61) | -0.48(-0.75) |
| Myalgia | 112 | 0.9(0.74-1.09) | 0.9(1.23) | 0.9(0.77) | -0.15(-0.42) |
| Hypertension | 110 | 0.74(0.62-0.9) | 0.74(9.43) | 0.75(0.64) | -0.41(-0.69) |
| Pain in extremity | 109 | 0.48(0.4-0.58) | 0.48(59.2) | 0.49(0.42) | -1.02(-1.3) |
| Death | 109 | 0.67(0.55-0.81) | 0.67(17.71) | 0.68(0.58) | -0.57(-0.85) |
| Loss of consciousness* | 107 | 1.26(1.03-1.52) | 1.26(5.31) | 1.24(1.06) | 0.31(0.03) |
| Cerebrovascular accident* | 100 | 1.97(1.61-2.41) | 1.97(44.3) | 1.9(1.6) | 0.93(0.63) |
| Chest pain | 99 | 0.75(0.61-0.91) | 0.75(8.24) | 0.75(0.64) | -0.41(-0.7) |
| Bronchitis* | 99 | 1.24(1.02-1.52) | 1.24(4.55) | 1.23(1.04) | 0.3(0.01) |
| Abdominal distension | 98 | 0.5(0.41-0.61) | 0.5(48.52) | 0.51(0.43) | -0.98(-1.27) |
| Therapy non-responder* | 95 | 1.7(1.38-2.09) | 1.7(25.57) | 1.66(1.39) | 0.73(0.42) |
| Seizure* | 94 | 2.16(1.75-2.67) | 2.16(54.43) | 2.08(1.74) | 1.05(0.75) |
| Liquid product physical issue* | 93 | 13.47(10.51-17.26) | 13.44(720.56) | 9.37(7.61) | 3.23(2.88) |
| Drug level below therapeutic | 93 | 1.1(0.89-1.35) | 1.1(0.81) | 1.1(0.92) | 0.13(-0.17) |
| Arthritis | 91 | 0.72(0.58-0.89) | 0.72(9.65) | 0.73(0.61) | -0.46(-0.76) |
| Drug delivery system malfunction* | 91 | 32.68(24.13-44.28) | 32.63(1278.98) | 15.5(12.02) | 3.95(3.58) |
| Paraesthesia | 90 | 0.73(0.59-0.9) | 0.73(8.74) | 0.74(0.62) | -0.44(-0.75) |
| Oropharyngeal pain | 89 | 0.5(0.4-0.61) | 0.5(44.02) | 0.51(0.43) | -0.98(-1.28) |
| Flushing | 89 | 1.22(0.98-1.51) | 1.22(3.31) | 1.21(1.01) | 0.27(-0.04) |
| Insomnia | 89 | 0.59(0.48-0.73) | 0.59(24.36) | 0.6(0.5) | -0.73(-1.04) |
| Intestinal stenosis | 88 | 0.85(0.68-1.05) | 0.85(2.41) | 0.85(0.71) | -0.23(-0.54) |
| Adverse event* | 87 | 1.83(1.47-2.27) | 1.83(30.48) | 1.77(1.48) | 0.83(0.51) |
| Hepatic enzyme increased | 85 | 1.23(0.99-1.53) | 1.23(3.51) | 1.22(1.02) | 0.29(-0.03) |

Abbreviation: Asterisks (*) indicate statistically significant signals in algorithm; ROR, reporting odds ratio; PRR, proportional reporting ratio; EBGM, empirical Bayesian geometric mean; EBGM05, the lower limit of the 95% CI of EBGM; IC, information component; IC025, the lower limit of the 95% CI of the IC; CI, confidence interval; PT, preferred term.
